# Synthesis and Evaluation of Antiproliferative Activity, Topoisomerase IIα Inhibition, DNA Binding and Non-Clinical Toxicity of New Acridine–Thiosemicarbazone Derivatives

**DOI:** 10.3390/ph15091098

**Published:** 2022-09-02

**Authors:** Gleyton Sousa, Maria C. F. de Almeida, Lucas L. Lócio, Vanda L. dos Santos, Daniel P. Bezerra, Valdenizia R. Silva, Sinara M. V. de Almeida, Alice Simon, Thiago da S. Honório, Lucio M. Cabral, Rosane N. Castro, Ricardo O. de Moura, Arthur E. Kümmerle

**Affiliations:** 1 Institute of Chemistry, Federal Rural University of Rio de Janeiro, Seropédica 23897-000, Brazil; 2 Department of Chemistry, State University of Paraíba, Campina Grande 58429-500, Brazil; 3 Department of Biological Sciences, State University of Paraíba, Campina Grande 58429-500, Brazil; 4 Laboratory of Tissue Engineering and Immunopharmacology (LETI), Institute Gonçalo Moniz (IGM), Foundation Oswaldo Cruz (Fiocruz), Salvador 40296-710, Brazil; 5 Molecular Biology Laboratory, University of Pernambuco (UPE), Garanhuns 55290-000, Brazil; 6 Department of Drugs and Pharmaceutics, Faculty of Pharmacy, Universidade Federal do Rio de Janeiro, Av. Carlos Chagas Filho, 373, CCS, Bss, Rio de Janeiro 21941-902, Brazil

**Keywords:** acridine–thiosemicarbazone, antiproliferative, topoisomerase IIα

## Abstract

In this study, we report the synthesis of twenty new acridine–thiosemicarbazone derivatives and their antiproliferative activities. Mechanisms of action such as the inhibition of topoisomerase IIα and the interaction with DNA have been studied for some of the most active derivatives by means of both in silico and in vitro methods, and evaluations of the non-clinical toxicities (in vivo) in mice. In general, the compounds showed greater cytotoxicity against B16-F10 cells, with the highest potency for DL-08 (IC_50_ = 14.79 µM). Derivatives DL-01 (77%), DL-07 (74%) and DL-08 (79%) showed interesting inhibition of topoisomerase IIα when compared to amsacrine, at 100 µM. In silico studies proposed the way of bonding of these compounds and a possible stereoelectronic reason for the absence of enzymatic activity for CL-07 and DL-06. Interactions with DNA presented different spectroscopic effects and indicate that the compound CL-07 has higher affinity for DNA (Kb = 4.75 × 10^4^ M^−1^; Ksv = 2.6 × 10^3^ M^−1^). In addition, compounds selected for non-clinical toxicity testing did not show serious signs of toxicity at the dose of 2000 mg/kg in mice; cytotoxic tests performed on leukemic cells (K-562) and its resistant form (K-562 Lucena 1) identified moderate potency for DL-01 and DL-08, with IC_50_ between 11.45 and 17.32 µM.

## 1. Introduction

Cancer is a term used for a set of more than 100 different types of malignant neoplasms [1], treated as a serious public health problem and characterized by the disordered growth of cells with high potential for metastasis [2]. In addition to factors such as aging and the growth of the population, numerous initiating and promoting agents of neoplasms are present in daily human life, such as radiation, organic solvents, contraceptives, alcoholic beverages, tobacco and others [2,3]. Cancer is characterized by a high rate of proliferation of defective cells, due to changes in the genetic material (deoxyribonucleic acid, DNA) of one or more cells. The imbalance in the regulatory mechanisms of cell division supports the development of this pathology [4,5,6]. Among the existing treatment options, chemotherapy is still considered one of the most efficient; however, associated with treatment, several adverse effects are reported [3,7,8]. Given these effects, there is a need for new therapeutic options for cancer, aiming at a possible reduction in toxicity and greater efficiency.

DNA topoisomerases (Topo) are a group of enzymes responsible for maintaining the topology of DNA during vital cellular processes, including replication, recombination, transcription, and repair systems [9,10]. Two types of topoisomerases are present in physiological functions in living beings: Topo I, which acts with cuts in one of the DNA strands, and Topo II, with cuts in the double strand of DNA. In mammals, two functionally distinct isoforms for Topo II are found: Topo IIα and Topo IIβ [9,11]. Topo IIα is mainly expressed in tissues with a high rate of cell proliferation and cancer cells, making it an interesting target for anticancer drugs [12,13,14,15,16]. Depending on the mode of interaction with the enzyme, topoisomerase inhibitors were classified into “topoisomerase poisons” and catalytic inhibitors [10,17]. The former inhibitors increase levels of topoisomerase II–DNA cleavage complexes, generating toxic intermediates to cellular metabolism, inducing mutagenic and lethal events [17,18]; the latter inhibitors decrease the activity of the enzyme, preventing it from forming the enzyme–DNA complex, either by obstructing DNA binding or by preventing topoisomerase from cleaving DNA [19]. Numerous topoisomerase II inhibitors were introduced in the oncology clinic as important drugs for cancer treatment, such as doxorubicin, etoposide, vosaroxine, mitoxantrone, daunorubicin and amsacrine [11,18,20]. It is important to highlight that most of these inhibitors are bifunctional, which potentiates the levels of DNA scission; the association of directional fragments to DNA and topoisomerase IIα may justify the anticancer activity found in these drugs [17,20,21,22,23].

Many works associating heterocyclic rings as potential bioactive fragments have been reported in recent years [24,25,26,27,28]. Among them, the acridine core is highlighted, an important scaffold for the development of chemotherapeutics. These derivatives also present a wide spectrum of biological activities, such as antibacterial [29,30,31,32], antimalarial [33,34,35], antitumor [4,21,36,37,38,39,40], among others. Its efficiency in intercalating DNA bases, mainly attributed to its planarity, and its capability of inhibiting topoisomerase enzymes, telomerase, and the proteasome complex of cells, would justify its potential against tumor cells [5,6,29,38]. Thiosemicarbazones are also widely explored in medicinal chemistry as linkers between pharmacophoric groups due to their electronic properties and reactivity [41,42,43,44,45]. Many biological activities have been reported for this group, such as antibacterial [46], antimalarial [47], antiviral [48], anticancer [38,42,45,49,50,51] and others. Their antiproliferative activity is often associated with the ability to bind and cleave DNA, induce apoptosis, and inhibit cellular enzymes, as well as their chelating ability against metal ions [41,42,43,45].

**Figure 1:**
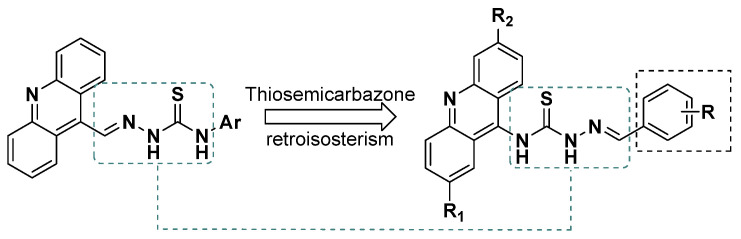
Structural design of new acridine–thiosemicarbazone derivatives.

Some studies have evaluated the biological antiproliferative profile of acridine and quinoline derivatives condensed with substituted aromatic thiosemicarbazone fragments [50,51,52,53,54]. Da Silva Filho indicated important inhibitions of Topo IIα in his studies, the acridine fragment was able to intercalate to the DNA; however, given the scaffold (Figure 1), no polar interaction with the residues of enzyme was identified in molecular docking. From these results, new acridine–thiosemicarbazone derivatives are proposed herein, designed by means of a retroisosterism strategy, envisioning additional interactions to the enzyme, mainly to the Arg^487^ residue, intrinsic to amsacrine docking [52,55]. Different electronic and/or electrostatic groups, proposed from the Topliss decision tree, were explored in R trying to understand their interferences in the antiproliferative activity, and in order to compare to the previously reported mechanism of action of the former class of compounds [56,57]. Additionally, given the biological reports of disubstituted acridines in drugs such as quinacrine [58] and pyronaridine [22], both with a mechanism involving DNA and Topo II, the new compounds were also proposed bearing the acridine core substitution (R_1_ and R_2_) in a similar way to the mentioned drugs. Besides the synthesis, also planned for these acridine–thiosemicarbazones was the evaluation of their antiproliferative activities, as well as a study of the interaction profile with DNA and Topo IIα inhibition for the most active compounds. Last but not least, as it is a recurring problem among available chemotherapeutics [7,59,60] and acridine derivatives [61,62], the acute and subchronic toxicity evaluation of the main compounds in mice were also examined.

## 2. Results and Discussion

### 2.1. Chemistry

**Scheme 1:**
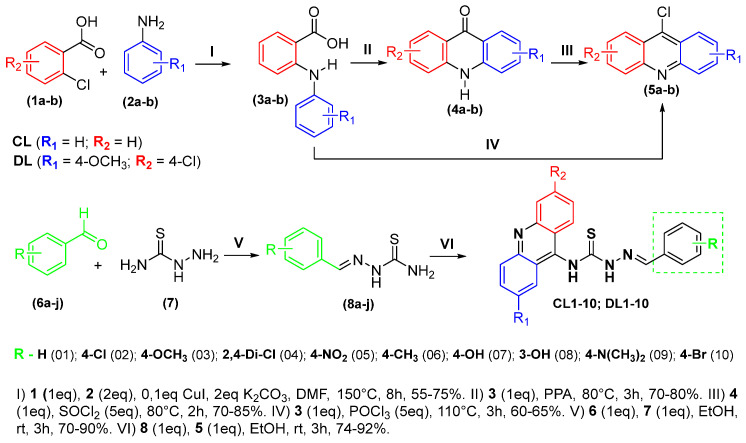
Synthesis of acridine–thiosemicarbazone derivatives.

The new acridine–thiosemicarbazone derivatives (**CL-01** to **CL-10** and **DL-01** to **DL-10**) were synthesized in four or five steps, depending on the chosen route to the acridine core (Scheme 1). First, compounds **3a-b** were obtained using 2-chlorobenzoic acid (**1a-b**) reacted with phenylamine derivatives **2a-b** via Ullmann reactions. Then, the Friedel–Crafts acylation reactions were conducted by PPA, in the case of the route through the isolated acridone intermediate (**4a-b**) or by phosphoryl chloride to produce the corresponding acridine intermediates **5a-b** [39,40,63]. Then, the synthesized thiosemicarbazides reacted with different aldehydes in the presence of a catalytic amount of AcOH, via a condensation reaction, forming thiosemicarbazones **8a-j** [64]. In the last step, the thiosemicarbazones obtained in the previous step underwent a new condensation, this time with acridine cores synthesized through an S_N_Ar-type nucleophilic substitution generating the final products [50,51,52]. The yields of the compounds obtained ranged from 74% to 92%.

To elucidate the chemical structure of the newly synthesized compounds, mass spectrometry and one- and two-dimensional ^1^H and ^13^C nuclear magnetic resonance (NMR) analyses were performed (ESI, Figures S1–S49). NMR spectroscopy confirmed all the expected hydrogens and carbons of the structure of the compounds, as well as their two-dimensional correlations. The purity and molecular mass of the new derivatives were confirmed by liquid chromatography coupled to mass spectrometry, LCMS.

The ^1^H NMR spectra showed singlets ranging from δ 9.53 to 8.93 ppm associated with thiosemicarbazone hydrogens, which presented variations in their displacements according to the type and electronic strength of the substituents in R of the benzylidene fragment, as well as with the changes in electron density in the acridine cores due to the substitutions in R_1_ and R_2_. Signals between δ 8.72 and 6.62 ppm were correlated to the aromatic hydrogens of the acridine and benzylidene cores, following the expected coupling characteristics for the substitution pattern. In addition, a characteristic singlet in the spectra between δ 7.09 and 6.71 ppm was associated with the methylidene hydrogen of the iminic bond. The possibility of configurational isomers in this region was ruled out by the observation of a unique singlet signal for each hydrogen from compounds in the ^1^H NMR spectra. The literature indicates numerous reports of preference for *E* isomerism, as well as semi-empirical calculations performed for the synthesized derivatives, showing a large energetic difference between the isomers, indicating *E* isomerism as exclusive [53,54,65]. Finally, the characteristic signals to the proposed aromatic substituents were found and signaled in the respective spectra contained in the Supplementary Material.

The ^13^C NMR spectrum for the new derivatives showed signals related to the thiocarbonyl group carbons (C=S) in the region between δ 181.1 and 180.0 ppm and imine carbons (HC=N) in the region between δ 144.9 and 137.6 ppm; both signals confirm the presence of the thiosemicarbazone linker in the derivatives. A peculiar characteristic of substituted acridines (DL), the presence of an electron-donating substituent group (EDG) affected the characteristic signals in the spectra of this series; alpha carbon (C-1) for methoxyl showed shifts between 99.4 and 98.4 ppm, and the downfield effect for C-2 carbon showed variations between 159.0 and 157.9 ppm. The expected signals for the benzylidene group after the proposed substitutions were found and are shown in the chemical descriptions of this work. The compounds showed purities above 98% in HPLC, and *m/z* [+1] (LC-MS) within that expected by the theoretical calculation for the new derivatives.

### 2.2. Cytotoxicity Assay

The in vitro cytotoxic activity of the compounds (Table 1) was evaluated against cancer cell lines HCT116 (human colon carcinoma), HepG2 (human hepatocellular carcinoma), B16F10 (murine melanoma carcinoma) and non-cancerous cell line MRC-5 (human lung fibroblast) using the Alamar blue assay after 72 h of incubation; doxorubicin (DOX) was used as a positive control. The unsubstituted derivatives on the acridine cores were encoded as CL, and the disubstituted derivatives as DL.

Among the evaluated compounds, the most promising cytotoxicity results were verified for those presenting non-halogenated monovalent substituents in R, highlighting hydroxyl as the best substituent. The introduction of this radical at *meta* and *para* positions of benzylidene moiety provided activities, mainly against B16-F10, with IC_50_ values between 14.79 (DL-08) and 21.32 (CL-08) µM. DL-08 was the most cytotoxic against the tumor and non-tumor strains studied in this work, indicating no selectivity for its activity. Notably, derivatives with bulky substituents at R (OCH_3_, NO_2_ and N(CH_3_)_2_) caused a drastic decrease in the antiproliferative activity, showing that the steric profile of the substituents at this position directly impacts the antiproliferative profile of this scaffold [50].

Structure–activity relationships (SAR) described for substituents in acridine cores indicated a considerable increase in antiproliferative activity with the presence of strong electron-donating and weak electron-withdrawing groups at positions 2 and 6, respectively [66]. In this work, derivatives DL-07 and DL-08 significantly increased their potency in strains HCT116 and HepG2, showing moderate antiproliferative activities with IC_50_ between 21.28 (DL-08, HepG2) and 28.46 µM (DL-07, HCT116) compared to their unsubstituted cores (CL) with IC_50_ between 39.16 (CL-08, HCT116) and 50.66 µM (CL-07, HepG2). In addition, halogenated compounds with bromo (DL-10, IC_50_ = 20.88 µM) and without substitution at R (DL-01, IC_50_ = 21.87 µM) showed some activity against HepG2.

### 2.3. Human Topoisomerase IIα Inhibition Assay

**Figure 2:**
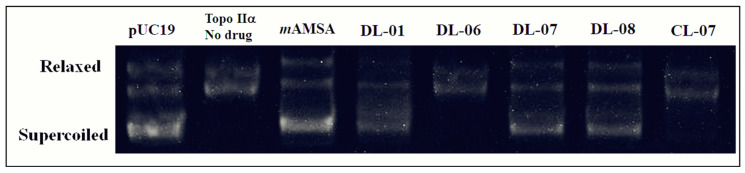
Effects of *m*AMSA and its acridine derivatives on topoisomerase IIα activity. Effects of derivatives and positive control on the inhibition of human topoisomerase IIα. Native supercoiled pUC19 was incubated for 30 min at 37 °C with 2 units of human Topo IIα in the absence (lane 2) or presence of ligands at a concentration 100 μM. Inhibition of Topo IIα-induced DNA relaxation by *m*AMSA (lane 3) was used as a positive control. DNA samples were run on an agarose gel followed by ethidium bromide staining.

Several studies have shown the ability of acridines to inhibit topoisomerases, being considered the main mechanism responsible for the antiproliferative activities of this scaffold [18,38,68,69]. The possibility of the new acridine derivatives to inhibit Topo IIα in its biological function of DNA double-strand relaxation was investigated in this work. This enzymatic inhibition can be accompanied by the presence of a supercoiled or relaxed DNA band, performed with the pUC19 plasmid, as shown in Figure 2. In this study, *m*AMSA was used as a positive inhibition control.

The derivatives DL-01, DL-07 and DL-08 were able to inhibit the enzymatic activity at a concentration of 100 µM. The mean intensity values obtained by densitometry, in direct correlation with *m*AMSA, were used to calculate the percentage of enzymatic inhibition to better analyze the effect, and these values are presented in Table 2. The absence of substitution on the acridine cores (CL-07), as well as sterically hindered substituents in the para position of the phenyl ring of thiosemicarbazone (DL-06), were characteristics associated with the non-inhibitory profile in Topo IIα for this class of compounds [51,52]. The compound DL-08 showed the highest inhibitory profile with 79%, with DL-07 having a percentage of 74% against the enzymatic activity.

### 2.4. Molecular Docking

In order to understand the observed in vitro results for Topo IIα inhibition, in silico studies were proposed to simulate a docking of the new derivatives from a crystallographic model of human topoisomerase IIα in complex with DNA available in the PDB (ID: 5gwk) [70]. This study can add important knowledge about the binding mode of the new derivatives in the enzyme, as well as stereo-electronic delimitations of its active site, which can help in understanding the absence of activities for compounds CL-07 and DL-06 [71]. Docking assays were based on the complexation site of Topo IIα with etoposide. The validation of the study was carried out both by re-docking studies (RMSD = 0.48) and by using amsacrine as a ligand, a positive control for this in vitro study with already described interactions with the enzyme, thus validating the parameters used in the Gold program for the calculations [72,73,74].

**Figure 3:**
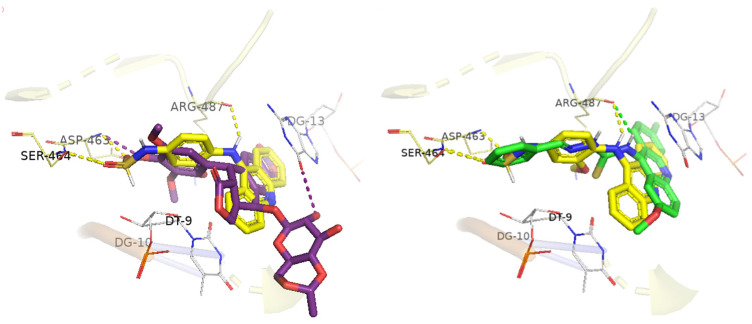
Final pose of molecular docking for some drugs and DL-01 with DNA–Topo IIα. On the left, final conformers of amsacrine (yellow) and etoposide (purple) complexed with human DNA–Topo IIα (PDB ID: 5gwk); on the right, a detailed illustration of the intermolecular interactions of DL-01 (green) compared to amsacrine. The dashes indicate the polar interactions made by each structure with the receptor and follow their respective colors.

Amsacrine and etoposide present the same behavior in docking with the DNA–Topo IIα binding site: they interact by means of polar interactions with amino acid residues Ser^464^, Asp^463^ and Arg^487^. In addition, electron π–π stacking interactions in the DNA region with the substituted aromatic group of the structures potentiate the stabilization of the docking poses, favoring the affinity for the studied binding site [72]. This information about the docking of the reference compounds is confirmed in Figure 3, where the docking of amsacrine is shown in comparison to etoposide and DL-01. The docking score for each compound and the types of interactions between ligands and the DNA–Topo IIα complex are described in Table 3.

Docking analysis revealed that hydrophobic interactions with the nucleotide base G^13^ on the F fragment of DNA are essential for docking and common to all compounds. The inhibitory capacity of Topo IIα compounds depends on the possibility of interacting in both the specific region of the enzyme and of DNA [75]. The DL-01, DL-07 and DL-08 derivatives showed, in addition to interactions with DNA, polar hydrogen bond interactions with the Arg^487^ residue, consistent with the reference compounds studied, at distances ranging from 2.4 Å (amsacrine) to 3.0 Å (DL-07). The compound DL-08 still showed polar interactions with the amino acids of the dyad (Ser^464^-Asp^463^), and for this reason, also considering the hydrophobic interactions described in Table 3, it presented the highest fit score of the study, 98.06 [72,73].

**Figure 4:**
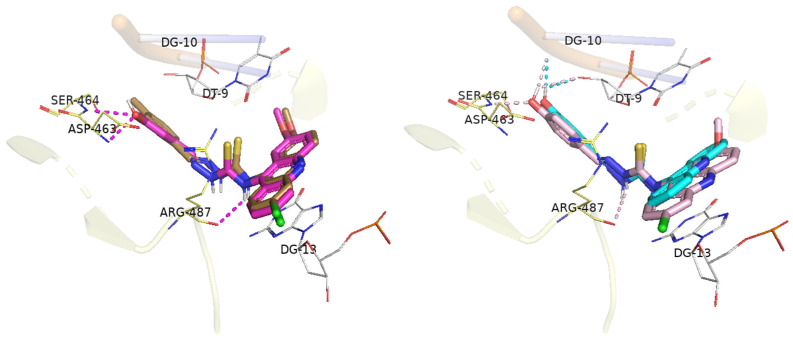
Final pose of molecular docking for acridine–thiosemicarbazone derivatives with DNA–Topo IIα. On the left, final conformers of DL-06 (bronze-yellow) and DL-08 (magenta) complexed with human DNA–Topo IIα (PDB ID: 5gwk); on the right, a detailed illustration of the intermolecular interactions of DL-07 (light pink) compared to CL-07 (cyan). The dashes indicate the polar interactions made by each structure with the receptor and follow their respective colors.

The final poses found in the coupling of acridine derivatives with DNA–Topo IIα are shown in Figure 3 and Figure 4. Docking results found for the compounds CL-07 and DL-06 raised two observations to be discussed, which may help in the understanding of their low scores in theoretical studies and the absence of enzymatic activities (in vitro). The first observation is the interference of the substituents inserted in R_1_ and R_2_ in the strength and mode of interaction with the DNA; changes in the angles and distances involved mainly in the stacking of π–π electrons with the base G^13^ of the DNA were noticeable when comparing the compounds CL-07 and DL-07. The non-substitution reduced the intensity of the interaction with the bases, causing an unfavorable change to the interactions with the residues Arg^487^ and Ser^464^, found for the compound DL-07 [76]. Additionally, substitution in the *para* portion of benzylidene can exceed the active site limit shown by crystallography; in this context, the compound DL-06 lost the ability to interact with the dyad, failing to carry out the polar interactions found, for example, in DL-08 with the enzyme (Ser^464^ and Asp^463^) in this region [51]. Finally, the compounds for hydroxy still showed additional polar interactions with the DNA strand fragments, T^9^ and G^10^.

### 2.5. DNA Interaction Studies

From the cytotoxicity results presented, some of the most active compounds (CL-07, DL-01 and DL-08) were selected for spectroscopic studies of interaction with DNA. We aimed to understand the influence of DNA intercalating on the activity found, as well as the correlations between the proposed structural modifications and their affinity with this anticancer mechanism, which is widely studied for this class of compounds [51,54]. Ethidium bromide is a DNA intercalating agent, and in this study, it was used as a positive control in all spectroscopic analyses.

#### 2.5.1. CT DNA Binding by UV–Vis Absorption Spectroscopy

Absorption spectroscopy is a convenient and simple method for probing the binding of small molecules to DNA. The interaction of selected acridine–thiosemicarbazone derivatives with CT DNA was studied in the 220–500 nm region. Binding constants (Kb) were calculated from [DNA]/(Ɛa − Ɛf) versus [DNA], in which Kb is given by the ratio of slope to the intercept [77,78]. Table 4 shows the intrinsic binding constants (Kb) exhibited by the compounds, as well as the characteristics linked to the calculation and the spectral changes identified. For this, the regions chosen were the ones with the highest absorption intensity above 300 nm, due to the lower interference in DNA absorption in this region (ESI, Figures S50 and S53).

In the presence of increasing concentrations of CT DNA (0 to 60 µM), hypochromic effects in the absorption of compounds (40 µM) were observed, indicating interactions with the CT DNA double helix. The intercalation mode is characterized by hypochromism and bathochromism due to π–π stacking interactions between the aromatic fragments and DNA base pairs [78,79,80,81]. The greatest hypochromic effects observed among the compounds studied by UV–Vis were for the hydroxylated compounds, CL-07 (25.52%) and DL-08 (14.79%), demonstrating that the *para*-hydroxyl group can potentialize the interactions between molecule and DNA [81].

The addition of DNA to acridine derivatives caused slight bathochromic (DL-01 and DL-08) and hypsochromic (CL-07) shifts. The binding constants of compounds with CT DNA (Kb) were calculated, with higher values indicating greater affinity for DNA [78]. The magnitudes of intrinsic binding constants (Kb) were 4.51 × 10^4^ (CL-07), 0.75 × 10^4^ (DL-01), 1.73 × 10^4^ (DL-08) and 5.56 × 10^4^ M^−1^ (EtBr). The results obtained for the positive control, as well as data from the literature, suggest that the compounds studied bind to DNA double helix with moderate affinities and have a preference for the intercalation mode [77,82,83].

#### 2.5.2. Circular Dichroism

Based on the chiral characteristic of DNA, the optical activity of the secondary structure of DNA was evaluated by circular dichroism (CD) in the presence of new acridine–thiosemicarbazone derivatives, as a continuation of the understanding of the potency and form in the interactions of these compounds with DNA [82,83,84]. The derivatives studied did not show considerable spectral changes in CD in Tris-HCl buffer solution (pH 7.4), being considered CD-inactive. The right helical conformation of CT DNA in the B form showed a characteristic CD spectrum with a positive band in the region of h-280 nm, related to the π–π stacking between the bases, and a negative band around 245 nm due to the right helix of this macromolecule [51,85].

**Figure 5:**
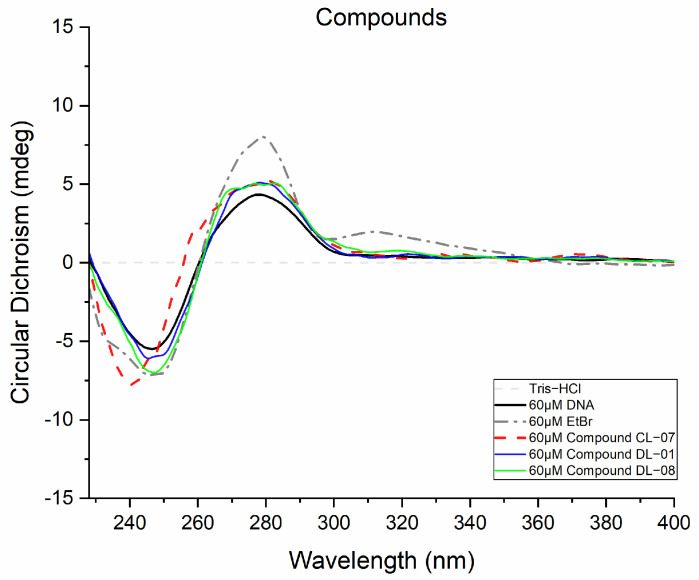
Circular dichroism of compounds and EtBr in the presence of CT DNA. Circular dichroism of DNA in the presence of acridine–thiosemicarbazone derivatives (60 µmol L^−1^), in Tris−HCl buffer (pH 7.4).

When an achiral compound and a chromophore bind to DNA, an induced circular dichroism (ICD) can appear within the adsorption band of the asymmetrically disturbed chromophore [82]. The DNA CD in the presence of EtBr (60 μM, Figure 5) generated a signal on the CD above 300 nm, associated with the hyperchromic effect in both the positive and negative characteristic bands. This result was used as a standard of potentiality and mode for the study [84]. Figure 5 shows the CD spectra of CT DNA (60 μM) in the absence and presence of up to 60 μM of compounds. In general, at the concentrations evaluated (20, 40 and 60 µM; ESI, Figures S54–S57), the CD spectra showed hyperchromic effects, with a predominance of the bathochromic shift for the DL series in both positive and negative bands, with a greater DNA sensitivity to the increase in [DL-08]. For compound CL-07, besides the common hyperchromic effect observed in this study, a characteristic hypsochromic profile emerged as the [CL-07]/[DNA] ratio was increased. These results confirm the greater affinity of compounds DL-08 and CL-07 for CT DNA when compared to DL-01. Regarding the interaction mode, the greatest hyperchromic effects found in the negative region are associated with intercalation [77,82,85].

#### 2.5.3. Fluorescent Studies with EtBr

**Figure 6:**
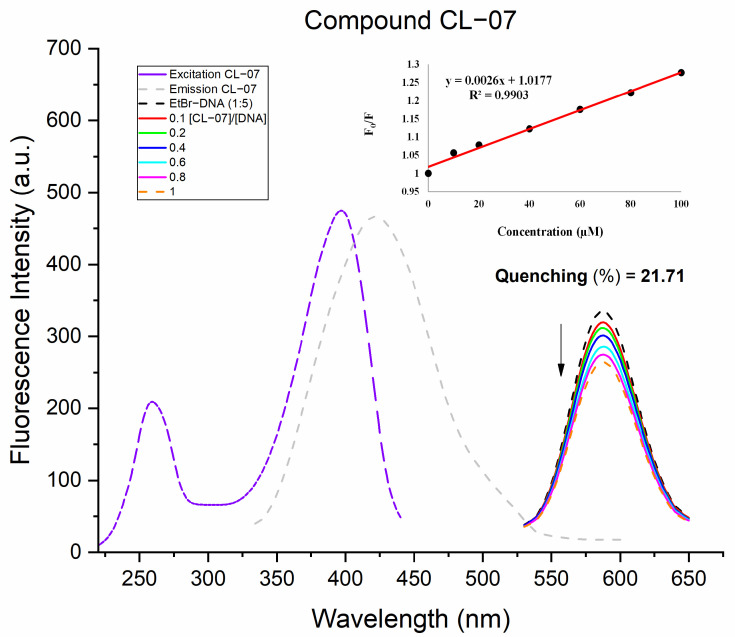
Fluorescence quenching spectra of CT DNA–EtBr upon the addition of compound CL−07. Emission spectrum of CL−07: excitation at 320 nm with an observation range from 330 to 600 nm. Excitation spectrum of CL−07: emission at 450 nm and measure range from 440 to 220 nm. Emission quenching curves of EtBr–DNA by CL−07: [EtBr] = 20 μmol L^−1^, [DNA] = 100 μmol L^−1^ (ratio 1:5), [CL−07] = 0–100 μmol L^−1^ (λex = 520 nm).

**Figure 7:**
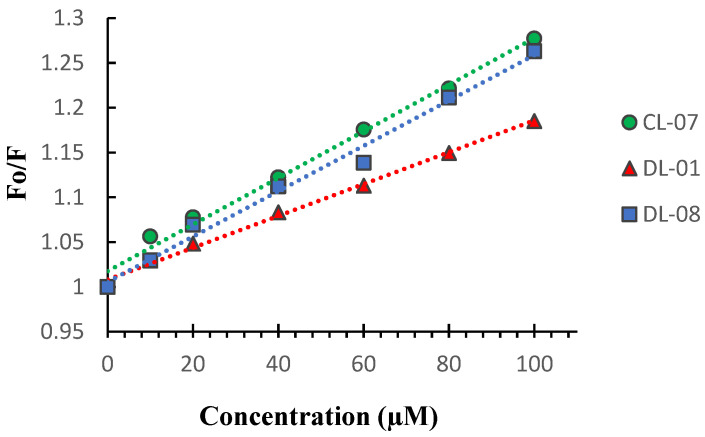
Steady-state fluorescence intensities of CT DNA–EtBr in the absence and presence of compounds.

EtBr is a weak fluorophore in an aqueous solvent, which has its fluorescence enhanced when it binds to DNA by the intercalative mode. The study of fluorescence quenching with EtBr is commonly used to assess the ability of compounds to interact with DNA by interfering with the fluorescence of the EtBr–DNA interaction (ESI, Figures S58 and S59) [78,80]. The EtBr–DNA complex in the presence and absence of CL-07 is shown in Figure 6, and the comparison of the quenching extent between studied compounds in terms of F/F_0_ vs. [compound] is illustrated in Figure 7. The results corroborate the order of magnitude presented in previous experiments, indicating CL-07 with the highest affinity for DNA with a quenching of 21.71% in the maximum studied [CL-07]/[DNA] ratio [86].

The calculated Stern–Volmer extinction constants (Ksv) (equation F_0_/F = 1 + Ksv[Q]) for the compounds, obtained from competitive emission studies with EtBr, are described in Table 5, showing Ksv values between 1.8 and 2.6 × 10^3^ mol L^−1^. These results indicate that the substitution profile in the benzylidene moiety interferes more with the intercalation capacity of the compounds than the increase in electron density in the acridine cores. However, these values indicate moderate potency to the target studied compared to other intercalators described in the literature [80,87].

### 2.6. Evaluation of the Non-Clinical Toxicity

Based on the low selectivity found in the in vitro cytotoxicity, we decided to perform a preclinical in vivo toxicological analysis on mice to understand the pharmacological profiles of the derivatives described herein. After oral administration of acridine–thiosemicarbazone derivatives to female mice, possible adverse clinical signs and mortality in the groups of animals submitted to the acute toxicity study were recorded (Table 6), as well as absolute body mass, food and water consumption, and the average masses of the organs of the animals treated with the compounds and the control group. These data are presented in the Supplementary Material (ESI, Tables S1 and S2).

According to the experiments, there was no death after oral administration of the compounds studied at a dose of 2000 mg/kg in female mice over the evaluated period of 14 days. There were also no significant macroscopic changes in the weight of the lungs, liver, kidneys, heart and spleen between the animals in the control group and those exposed to derivatives. However, changes in the water consumption of animals treated with DL-08 and in the food consumption of those treated with DL-01 were underlined by the study statistics; such conditions can be explained by the possibility of xenobiotics acting in pathways that involve the induction of satiety in the body. In general, the compounds presented LD_50_ > 2.000 mg/kg and were included in category V (LD_50_, median lethal dose, between 2.000 and 5.000 mg/kg) in the GHS classification (Globally Harmonized System) according to OECD guideline 423. This classification points to relative pharmacological safety for the new derivatives in the studies performed [88,89,90,91].

### 2.7. Cytotoxicity Assay on Leukemic Normal and Resistant Cells

Like amsacrine, acridine and thiosemicarbazone derivatives have shown potency against leukemic cancer cell lines [50,53,54,92]. Derivatives CL-07, DL-01 and DL-08 were evaluated in this work regarding their cytotoxic profile against K-562 cells and their resistant isoform, K562-Lucena 1. Interestingly, the evaluated derivatives of the DL series (6-chloro-2-methoxyacridin substituted) showed the highest activity profile against leukemic cells, including its resistant isoform. DL-01 is the most potent derivative against K-562 (IC_50_ = 11.45 µM) and K562-Lucena 1 (IC_50_ = 16.46 µM, 2 times more selective than MRC-5), followed by DL-08 (K-562, IC_50_ = 17.32 µM; K562-Lucena 1, IC_50_ = 17.12 µM). CL-07 showed a lower cytotoxic profile, with IC_50_ of 45.45 and 43.39 µM in K-562 and K562-Lucena 1, respectively [93,94]. Curiously, DL-01 and DL-08 derivatives inhibited Topo IIα and bound DNA, while CL-07 could not inhibit Topo IIα, and this discrepancy could explain the potency differences between **D** and **C** series.

## 3. Materials and Methods

### 3.1. Chemicals, Reagents, and Equipment

The reagents used to obtain the compounds were 2-chlorobenzoic acid, 2,4-dichlorobenzoic acid, aniline, 4-methoxyaniline benzaldehyde, 4-chlorobenzaldehyde, 4-methoxybenzaldehyde, 2,4-dichlorobenzaldehyde, 4-nitrobenzaldehyde, 4-methylbenzaldehyde, 4-hydroxybenzaldehyde, 3-hydroxybenzaldehyde, 4-(dimethylamino) benzaldehyde and 4-bromobenzaldehyde. The solvents absolute ethanol, 1,4-dioxane, acetonitrile, methanol, glacial acetic acid, *n*-hexane, dichloromethane and ethyl acetate were used. All reagents and solvents were supplied by Sigma-Aldrich (Saint Louis, MO, USA).

To structurally characterize the chemical compounds, nuclear magnetic resonance spectra of hydrogen (^1^H NMR) and carbon (^13^C NMR) were acquired by a 400 MHz or 500 MHz Bruker Advance spectrometer, with DMSO-*d_6_* or CDCl_3_ as solvents and TMS as an internal standard. High-performance liquid chromatography (HPLC) analysis was carried out using a Prominence-Shimadzu with a loop of 10 μL and column Phenomenex C18 (100 mm × 4.6 mm × 3 μm) in MeOH (70%): 30% H_2_O (0.1% HCO_2_H). Mass spectrometry was performed on the CG-MS 2020 (Shimadzu, Kyoto, Japan).

The melting points were recorded with a Fisatom model 431D fusiometer and are uncorrected. Thin-layer chromatography (TLC) with 0.25 mm thick Merk 60 F254 silica gel plates were used to follow reactions.

Calf thymus DNA (CT DNA), pUC19 DNA plasmid, recombinant human topoisomerase IIα (p170), amsacrine and ethidium bromide (EtBr) used in the interaction analyses were purchased from Sigma-Aldrich. Stock solutions of the compounds for interaction with DNA were prepared with methanol as a solvent. Subsequent dilutions were made in Tris-HCl buffer (0.1 M; pH 7.6) and the UV–Vis, fluorescence emission and CD spectra were acquired in JASCO Spectrophotometer J-818.

### 3.2. Synthesis of the Novel Derivatives

#### 3.2.1. Synthesis of 9-Chloroacridine Cores

Route A:

Synthesis of the substituted or unsubstituted acridine cores passes through an Ullman coupling between the 2-chlorobenzoic acid and amines, forming *N*-phenylanthranilinic acids, which undergo an intramolecular cyclization with polyphosphoric acid (PPA), yielding acridones, and finally the chlorination process, forming 9-chloro acridines. A solution of *o*-chlorobenzoic acid (6.0 mmol), aniline derivative (12.0 mmol), copper powder (0.6 mmol) and potassium carbonate (12.0 mmol) in DMF (20 mL) was heated under reflux for 8 h. After completion of the reaction (monitored by TLC), the reaction mixture was cooled down to room temperature, poured into hot water and boiled in the presence of activated charcoal for 15 min. Then, it was filtered through celite, and the filtrate was acidified with HCl to obtain a precipitated product that was purified using recrystallization from ethanol to obtain pure products (55–75%). After, a mixture of appropriate *N*-phenylanthranilinic acids (2 mmol) and polyphosphoric acid (20 mmol) was heated at 100 °C for 3 h. Upon completion, the reaction mixture was poured into hot water (100 mL). After cooling to room temperature, the mixture was made alkaline by ammonia solution. The obtained yellow precipitates (acridones) were filtered off, washed with hot water and purified using recrystallization from acetic acid to obtain the pure products (70–80%) [95]. Lastly, a suspension of acridone (5 mmol) in SOCl_2_ (6 mL) containing DMF (two drops) was stirred at reflux for 2 h. The solution was evaporated in vacuo, and residual traces of SOCl_2_ were removed by the addition of dry CH_2_Cl_2_ and complete evaporation of all solvents to give the crude product as a yellow powder, with no need for purification (70–85%) [63].

Route B:

Friedel–Crafts acylation reactions were also conducted using phosphoryl chloride (POCl_3_) for the synthesis of the corresponding acridine cores. The *N*-phenylanthranilinic acid derivatives (5 mmol) were cyclized intramolecularly to 9-chloroacridine derivatives by refluxing them in 5 mL of POCl_3_ for 4 h and the reaction was monitored by TLC. After completion, the excess POCl_3_ was removed by a rotary evaporator under reduced pressure. To the crude reaction mass, crushed ice was added, and pH was adjusted to ~7 by adding saturated bicarbonate solution. The separated solid was filtered, dried and purified by flash column chromatography with silica gel (60–120 mesh size) using 5–10% hexane:ethyl acetate as eluent to obtain the corresponding 9-chloroacridine derivatives (60–65%) [39,40].

#### 3.2.2. Obtaining Thiosemicarbazone Intermediates

Thiosemicarbazone intermediate compounds were obtained by a condensation reaction. In a round-bottom flask, equimolar amounts of thiosemicarbazide (5 mmol) and aldehydes (5 mmol) were mixed in absolute ethanol (20 mL) with a catalytic amount of glacial acetic acid (10 drops). The reaction was initially stirred under gentle heating (40 °C) (10 min), then at room temperature for about 3 h. After reaction completion, the resultant whiteish precipitate was filtered out and then crystallized in EtOH/H_2_O [64].

#### 3.2.3. Obtaining Acridine–Thiosemicarbazone Derivatives (CL 01-10 and DL 01-10)

The synthesis of the new acridine–thiosemicarbazone derivatives was carried out through a condensation reaction with equimolar proportions of thiosemicarbazones and acridines cores as reagents obtained previously. A solution of acridines (1.0 mmol) and thiosemicarbazone intermediates (1.0 mmol) in ethanol (20 mL) was heated under reflux for 1–4 h. Some compounds showed better yields in dioxane, as in the case of hydroxylated compounds. After completion of the reaction, monitored by TLC, the reaction mixture was cooled down to room temperature, filtered, and an amorphous solid (CL1-10 and DL1-10) was obtained, which was crystallized in an EtOH/H_2_O system [50,51,52].

#### 3.2.4. (E)-N-(acridin-9-yl)-2-benzylidenehydrazine-1-carbothioamide (**CL-01**)

Orange solid; yield: 86%. MP: 162–163 °C. R_f_ 0.50 (*n*-hexane/ethyl acetate, 6:4). **^1^H NMR (500 MHz, DMSO):** δ ppm 9.39 (s, 1H, NH), 9.28 (s, 1H, NH), 8.58 (d, J = 8.8 Hz, 2H, H-11, H-4), 8.20–8.14 (m, 2H, H-3, H-12), 7.84 (d, J = 4.1 Hz, 4H, H-1, H-2, H-13, H-14), 7.78 (d, J = 7.7 Hz, 2H, H-22, H-26), 7.40–7.28 (m, 3H, H-23, H-24, H-25), 6.98 (s, 1H, H-20). **^13^C NMR (125 MHz, DMSO-*d_6_*):** δ ppm: 180.74 (Cq), 144.87 (Cq), 144.42 (CH), 135.22 (CH) 133.80 (Cq), 131.08 (CH), 129.33 (CH), 128.94 (CH), 128.56 (CH), 124.99 (Cq), 124.54 (CH). **HPLC purity:** 98%. **LRMS E*xact Mass*** calcd for C_21_H_16_N_4_S: 356.110; *m*/*z* [M]^+^ found: 357 (100%); 358 (50%); 359 (15%).

#### 3.2.5. (E)-N-(acridin-9-yl)-2-(4-chlorobenzylidene)hydrazine-1-carbothioamide (**CL-02**)

Orange solid; yield: 81%. MP: 176–177 °C. R_f_ 0.57 (*n*-hexane/ethyl acetate, 6:4). **^1^H NMR (400 MHz, DMSO-*d_6_*):** δ ppm 9.43 (s, 1H, NH), 9.37 (s, 1H, NH), 8.59 (d, J = 8.8 Hz, 2H, H-11, H-4), 8.18 (dt, J = 8.5, 4.0 Hz, 2H, H-1, H-14), 7.90–7.81 (m, 6H, H-2, H-3, H-12, H-13, H-23, H-25), 7.44–7.36 (m, 2H, H-22, H-26), 7.00 (s, 1H, H-20). **^13^C** **NMR** **(100 MHz, DMSO-*d_6_*):** δ ppm 180.75 (Cq), 143.18 (CH), 135.59 (Cq), 132.83 (Cq), 130.27 (CH), 129.38 (CH), 129.04 (CH), 124.94 (Cq), 124.52 (CH). **HPLC purity:** 99%. **LRMS E*xact Mass*** calcd for C_21_H_15_ClN_4_S: 390.071; *m*/*z* [M]^+^ found: 391 (100%); 392 (38%); 393 (60%).

#### 3.2.6. (E)-N-(acridin-9-yl)-2-(4-methoxybenzylidene)hydrazine-1-carbothioamide (**CL-03**)

Orange solid; yield: 91%. MP: 169–170 °C. Rf 0.46 (*n*-hexane/ethyl acetate, 6:4). **^1^H NMR (400 MHz, DMSO-*d_6_*):** δ ppm: 9.33 (s, 1H, NH), 9.26 (s, 1H, NH), 8.61 (d, J = 8.9 Hz, 2H, H-11, H-4), 8.19 (ddd, J = 8.6, 4.7, 3.4 Hz, 2H, H-3, H-12), 7.86 (d, J = 3.6 Hz, 4H, H-1, H-4, H-13, H-14), 7.74 (d, J = 8.9 Hz, 2H, H-22, H-26), 6.95 (s, 1H, H-20), 6.88 (d, J = 9.0 Hz, 2H, H-23, H-25). **^13^C** **NMR** **(100 MHz, DMSO-*d_6_*):** δ ppm 180.46 (Cq), 161.72 (Cq), 144.47 (CH), 135.45 (Cq), 130.38 (CH), 129.35 (CH), 126.39 (Cq), 125.15 (Cq), 124.70 (CH), 114.42 (CH), 55.79 (OCH_3_). **HPLC purity:** 99%. **LRMS E*xact Mass*** calcd for C_22_H_18_N_4_OS: 386.120; *m*/*z* [M]^+^ found: 387 (100%); 388 (40%); 389 (14%).

#### 3.2.7. (E)-N-(acridin-9-yl)-2-(2,4-dichlorobenzylidene)hydrazine-1-carbothioamide (**CL-04**)

Mustard yellow solid; yield: 77%. MP: 172–173 °C. R_f_ 0.46 (*n*-hexane/ethyl acetate, 7:3). **^1^H** **NMR (400 MHz, DMSO-*d_6_*):** δ ppm 9.49 (s, 1H, NH), 9.37 (s, 1H, NH), 8.72 (d, J = 8.72, 1H, H-26), 8.46 (d, J = 8.8 Hz, 2H, H-11, H-4), 8.08 (ddd, J = 8.72, 5.5, 2.5 Hz, 2H, H-3, H-12), 7.82–7.79 (m, 4H, H-1, H-4, H-13, H-14), 7.51 (d, J = 8.2 Hz, 2H, H-23, H-25), 6.89 (s, 1H, H-20). **^13^C NMR (100 MHz, DMSO-*d_6_*):** δ ppm 181.08 (Cq), 147.02 (Cq), 138.19 (CH), 136.18 (Cq), 134.55 (Cq), 133.67 (CH), 130.15 (CH), 129.84 (Cq), 129.59 (CH), 129.14 (CH), 128.27 (CH), 124.13 (Cq), 124.01 (CH). **HPLC purity:** 99%. **LRMS E*xact Mass*** calcd for C_21_H_14_Cl_2_N_4_S: 424.032; *m*/*z* [M]^+^ found: 425 (100%); 426 (34%); 427 (83%); 428 (20%).

#### 3.2.8. (E)-N-(acridin-9-yl)-2-(4-nitrobenzylidene)hydrazine-1-carbothioamide (**CL-05**)

Mustard yellow solid; yield: 78%. MP: 189 °C. R_f_ 0.50 (*n*-hexane/ethyl acetate, 6:4). **^1^H** **NMR (500 MHz, DMSO-*d_6_*):** δ ppm 9.53 (s, 1H, NH), 9.46 (s, 1H, NH), 8.53 (d, J = 8.9 Hz, 2H, H-11, H-4), 8.17–8.11 (m, 4H, H-3, H-12, H-23, H-25), 8.09 (d, J = 8.7 Hz, 2H, H-22, H-26), 7.81 (d, J = 4.0 Hz, 4H, H-1, H-2, H-13, H-14), 7.09 (s, 1H, H-20). **^13^C** **NMR** **(125 MHz, DMSO-*d_6_*):** δ ppm 181.11 (Cq), 148.49 (Cq), 141.79 (CH), 140.20 (Cq), 129.52 (CH), 129.21 (CH), 124.63 (Cq), 124.23 (CH), 124.04 (CH). **HPLC purity:** 99%. **LRMS E*xact Mass*** calcd for C_21_H_15_Cl_2_N_5_O_2_S: 401.095; *m*/*z* [M]^+^ found: 402 (100%); 403 (46%).

#### 3.2.9. (E)-N-(acridin-9-yl)-2-(4-methylbenzylidene)hydrazine-1-carbothioamide (**CL-06**)

Orange solid; yield: 90%. MP: 170–171 °C. R_f_ 0.45 (*n*-hexane/ethyl acetate, 6:4). **^1^H** **NMR (400 MHz, DMSO-*d_6_*):** δ ppm 9.36 (s, 1H, NH), 9.26 (s, 1H, NH), 8.57 (d, J = 8.8 Hz, 2H, H-11, H-4), 8.17 (dt, J = 8.5, 4.0 Hz, 2H, H-3, H-12), 7.85 (d, J = 4.0 Hz, 4H, H-1, H-2, H-13, H-14), 7.68 (d, J = 8.2 Hz, 2H, H-22, H-26), 7.14 (d, J = 8.0 Hz, 2H, H-23, H-25), 6.94 (s, 1H, H-20), 2.28 (s, 3H, CH_3_). **^13^C NMR (100 MHz, DMSO-*d_6_*):** δ ppm 180.63 (Cq), 144.51 (CH), 141.06 (Cq), 135.21 (CH), 131.12 (Cq), 129.56 (CH), 129.31 (CH), 128.58 (CH), 125.04 (Cq), 124.58 (CH), 21.51 (CH_3_). **HPLC purity:** 99%. **LRMS E*xact Mass*** calcd for C_21_H_15_Cl_2_N_5_O_2_S: 370.125; *m*/*z* [M]^+^ found: 371 (100%); 372 (60%); 373 (15%).

#### 3.2.10. (E)-N-(acridin-9-yl)-2-(4-hydroxybenzylidene)hydrazine-1-carbothioamide (**CL-07**)

Orange solid; yield: 91%. MP: 183–184 °C. R_f_ 0.43 (*n*-hexane/ethyl acetate, 5:5). **^1^H** **NMR (500 MHz, DMSO-*d_6_*):** δ ppm 9.26 (s, 1H, NH), 9.16 (s, 1H, NH), 8.56 (d, J = 8.8 Hz, 2H, H-11, H-4), 8.21–8.12 (m, 2H, H-1, H-14), 7.84 (d, J = 3.8 Hz, 4H, H-2, H-3, H-12, H-13), 7.60 (d, J = 8.8 Hz, 2H, H-22, H-26), 6.87 (s, 1H, H-20), 6.69 (d, J = 8.6 Hz, 2H, H-23, H-25). **^13^C** **NMR** **(125 MHz, DMSO-*d_6_*):** δ ppm 180.45 (Cq), 160.42 (Cq), 144.66 (CH), 134.93 (CH), 130.48 (CH), 129.16 (CH), 125.09 (Cq), 124.73 (Cq), 124.62 (CH), 115.81 (CH). **HPLC purity:** 98%. **E*xact Mass*** calcd for C_21_H_16_N_4_OS: 372.104; *m*/*z* [M]^+^ found: 373 (100%); 374 (26%).

#### 3.2.11. (E)-N-(acridin-9-yl)-2-(3-hydroxybenzylidene)hydrazine-1-carbothioamide (**CL-08**)

Rust orange solid; yield: 92%. MP: 145 °C. R_f_ 0.54 (*n*-hexane/ethyl acetate, 5:5). **^1^H** **NMR (500 MHz, DMSO-*d_6_*):** δ ppm 9.30 (s, 1H, NH), 9.14 (s, 1H, NH), 8.47 (d, J = 8.8 Hz, 2H, H-11, H-4), 8.09 (d, J = 8.8 Hz, 2H, H-3, H-12), 7.81–7.77 (m, 4H, H-1, H-2, H-13, H-14), 7.16 (d, J = 9.3 Hz, 2H, H-22, H-26), 7.10 (t, J = 7.9 Hz, 1H, H-25), 6.82 (s, 1H, H-20), 6.79 (d, J = 8.2 Hz, 1H, H-24). **^13^C** **NMR** **(125 MHz, DMSO-*d_6_*):** δ ppm 180.94 (Cq), 157.95 (Cq), 144.30 (CH), 135.07 (Cq), 133.89 (CH), 129.91 (CH), 128.90 (CH), 124.77 (Cq), 124.31 (CH), 119.68 (CH), 118.20 (CH), 114.95 (CH). **HPLC purity:** 98%. **LRMS E*xact Mass*** calcd for C_21_H_16_N_4_OS: 372.104; *m*/*z* [M]^+^ found: 373 (100%); 374 (30%).

#### 3.2.12. (E)-N-(acridin-9-yl)-2-(4-(dimethylamino)benzylidene)hydrazine-1-carbothioamide (**CL-09**)

Rust orange solid; yield: 88%. MP: 158–159 °C. R_f_ 0.43 (*n*-hexane/ethyl acetate, 6:4). **^1^H** **NMR (500 MHz, DMSO-*d_6_*):** δ ppm 9.18 (s, 1H, NH), 9.10 (s, 1H, NH), 8.50 (d, J = 8.8 Hz, 2H, H-11, H-4), 8.11 (d, J = 7.8 Hz, 2H, H-3, H-12), 7.85–7.77 (m, 4H, H-1, H-2, H-13, H-14), 7.57 (d, J = 8.5 Hz, 2H, H-22, H-26), 6.80 (s, 1H, H-20), 6.63 (d, J = 8.5 Hz, 2H, H-23, H-25), 2.92 (s, 6H, N(CH_3_)_2_). **^13^C NMR (125 MHz, DMSO-*d_6_*):** δ ppm 180.25 (Cq), 144.95 (CH), 134.36 (CH), 130.03 (CH), 128.92 (CH), 125.07 (Cq), 124.61 (CH), 112.03(CH). **HPLC purity:** 98%. **LRMS E*xact Mass*** calcd for C_23_H_21_Cl_2_N_5_S: 399.152; *m*/*z* [M]^+^ found: 400 (100%); 401 (30%).

#### 3.2.13. (E)-N-(acridin-9-yl)-2-(4-bromobenzylidene)hydrazine-1-carbothioamide (**CL-10**)

Orange solid; yield: 78%. MP: 178–179 °C. R_f_ 0.43 (*n*-hexane/ethyl acetate, 7:3). **^1^H NMR (500 MHz, DMSO-*d_6_*):** δ ppm 9.43 (s, 1H, NH), 9.36 (s, 1H, NH), 8.59 (d, J = 8.8 Hz, 2H, H-11, H-4), 8.18 (dt, J = 8.6, 4.0 Hz, 2H, H-3, H-12), 7.85 (d, J = 4.0 Hz, 4H, H-1, H-2, H-13, H-14), 7.78 (d, J = 8.6 Hz, 2H, H-22, H-26), 7.53 (d, J = 8.7 Hz, 2H, H-23, H-25), 6.98 (s, 1H, H-20). **^13^C NMR (125 MHz, DMSO-*d_6_*):** δ ppm 180.76 (Cq), 143.29 (CH), 135.19 (CH), 133.17 (Cq), 131.95 (CH), 130.47 (CH), 129.37 (CH), 124.93 (Cq), 124.52 (CH). **HPLC purity:** 99%. **LRMS E*xact Mass*** calcd for C_21_H_15_Cl_2_BrN_4_S: 434.020; *m*/*z* [M]^+^ found: 435 (100%); 437 (100%).

#### 3.2.14. (E)-2-benzylidene-N-(6-chloro-2-methoxyacridin-9-yl)hydrazine-1-carbothioamide (**DL-01**)

Orange solid; yield: 74%. MP: 154–155 °C. R_f_ 0.54 (*n*-hexane/ethyl acetate, 6:4). **^1^H NMR (300 MHz, DMSO-*d_6_*):** δ ppm 9.16 (s, 1H, NH), 9.07 (s, 1H, NH), 8.24 (d, J = 1.7 Hz, 1H, H-11), 8.13 (d, J = 9.4 Hz, 1H, H-4), 7.86–7.80 (m, 2H, H-13, H-14), 7.67–7.54 (m, 3H, H-3, H-22, H-26), 7.33 (dd, J = 9.2, 6.9 Hz, 3H, H-23, H-24, H-25), 6.91 (s, 1H, H-20), 6.78 (d, J = 2.7 Hz, 1H, H-1), 3.82 (s, 3H, OCH_3_). **^13^C NMR (75 MHz, DMSO-*d_6_*):** δ ppm 180.43 (Cq), 158.30 (Cq), 147.37 (Cq), 147.33 (Cq), 142.85 (CH), 138.76, 134.22, 133.44, 131.45, 130.35, 128.42 (CH), 128.11 (CH), 127.75, 126.27, 125.26, 124.98, 122.51, 122.51, 98.57 (CH), 55.67 (OCH_3_). **HPLC purity:** 98%. **LRMS E*xact Mass*** calcd for C_22_H_17_ClN_4_OS: 420.081; *m*/*z* [M]^+^ found: 421 (100%); 422 (30%); 423 (40%).

#### 3.2.15. (E)-N-(6-chloro-2-methoxyacridin-9-yl)-2-(4-chlorobenzylidene)hydrazine-1-carbothioamide (**DL-02**)

Orange solid; yield: 80%. MP: 144–145 °C. R_f_ 0.58 (*n*-hexane/ethyl acetate, 6:4). **^1^H NMR (300 MHz, DMSO-*d_6_*):** δ ppm 9.21 (s, 1H, NH), 9.16 (s, 1H, NH), 8.32 (d, J = 1.8 Hz, 1H, H-11), 8.21 (d, J = 9.6 Hz, 1H, H-4), 7.87 (d, J = 8.3 Hz, 2H, H-22, H-26), 7.72–7.56 (m, 3H, H-3, H-13, H-14), 7.37 (d, J = 8.2 Hz, 2H, H-23, H-25), 6.93 (s, 1H, H-20), 6.78 (d, J = 2.8 Hz, 1H, H-1), 3.82 (s, 3H, OCH_3_). **^13^C NMR (75 MHz, DMSO-*d_6_*):** δ ppm 180.38 (Cq), 158.44 (Cq), 146.42 (Cq), 146.36 (Cq), 141.89 (CH), 139.78, 134.91, 132.44, 130.49, 129.82 (CH), 128.71, 128.47 (CH), 126.96, 126.81, 125.42, 125.12, 122.63, 98.72 (CH), 55.77 (OCH_3_). **HPLC purity:** 98%. **LRMS E*xact Mass*** calcd for C_22_H_16_Cl_2_N_4_OS: 454.042; *m*/*z* [M]^+^ found: 455 (100%); 456 (26%); 457 (54%).

#### 3.2.16. (E)-N-(6-chloro-2-methoxyacridin-9-yl)-2-(4-methoxybenzylidene)hydrazine-1-carbothioamide (**DL-03**)

Orange solid; yield: 88%. MP: 148 °C. R_f_ 0.46 (*n*-hexane/ethyl acetate, 6:4). **^1^H** NMR **(300 MHz, DMSO-*d_6_*):** δ ppm 9.16 (s, 1H, NH), 9.08 (s, 1H, NH), 8.39 (d, J = 1.9 Hz, 1H, H-11), 8.28 (d, J = 9.5 Hz, 1H, H-4), 7.79–7.69 (m, 4H, H-3, H-13, H-22, H-26), 7.67 (d, J = 9.1 Hz, 1H, H-14) 6.92 (s, 1H, H-20), 6.89–6.82 (m, 3H, H-1, H-23, H-25), 3.83 (s, 3H, OCH_3_), 3.73 (s, 3H, OCH_3_). **^13^C** NMR **(75 MHz, DMSO-*d_6_*):** δ ppm 180.00 (Cq), 161.15 (Cq), 158.55 (Cq), 145.11, 144.94, 143.39 (CH), 141.68, 135.86, 129.90 (CH), 128.97, 127.89, 125.96, 125.82, 125.43, 122.98, 113.91 (CH), 99.08 (CH), 55.86 (OCH_3_), 55.28 (OCH_3_). **HPLC purity:** 99%. **LRMS E*xact Mass*** calcd for C_23_H_19_ClN_4_O_2_S: 450.092; *m*/*z* [M]^+^ found: 451 (100%); 452 (35%); 453 (45%).

#### 3.2.17. (E)-N-(6-chloro-2-methoxyacridin-9-yl)-2-(2,4-dichlorobenzylidene)hydrazine-1-carbothioamide (**DL-04**)

Orange solid; yield: 80%. MP: 192–193 °C. R_f_ 0.62 (*n*-hexane/ethyl acetate, 6:4). **^1^H NMR (300 MHz, DMSO-*d_6_*): 7.54–7.46 (m, 2H), 6.84 (s, 1H), 6.76 (d, J = 2.8 Hz, 1H), 3.84 (s, 3H).** δ ppm 9.35 (s, 1H, NH), 9.24 (s, 1H, NH), 8.60 (d, J = 8.6 Hz, 1H, H-26), 8.34 (d, J = 2.2 Hz, 1H, H-11), 8.22 (d, J = 9.5 Hz, 1H, H-4), 7.72–7.62 (m, 2H, H-3, H-13), 7.61 (d, J = 9.3 Hz, 1H, H-14), 7.54–7.46 (m, 2H, H-23, H-25), 6.84 (s, 1H, H-20), 6.76 (d, J = 2.8 Hz, 1H, H-1), 3.84 (s, 3H, OCH_3_). **^13^C NMR (75 MHz, DMSO-*d_6_*):** δ ppm 181.07 (Cq), 159.06 (Cq), 147.89 (Cq), 138.42 (Cq), 137.64 (CH), 136.09 (Cq), 134.85 (Cq), 134.57 (Cq), 132.27, 130.02, 129.82 (Cq), 129.56, 129.29, 128.56, 128.25, 126.99, 125.27, 125.23 (Cq), 122.47 (CH), 98.87 (CH), 56.27 (OCH_3_). **HPLC purity:** 99%. **LRMS E*xact Mass*** calcd for C_22_H_15_Cl_3_N_4_OS: 488.003; *m*/*z* [M]^+^ found: 489 (100%); 491 (100%); 492 (20%).

#### 3.2.18. (E)-N-(6-chloro-2-methoxyacridin-9-yl)-2-(4-nitrobenzylidene)hydrazine-1-carbothioamide (**DL-05**)

Orange solid; yield: 86%. MP: 164–165 °C. R_f_ 0.61 (*n*-hexane/ethyl acetate, 7:3). ^1^H NMR **(300 MHz, DMSO-*d_6_*):** δ ppm 9.32 (s, 1H, NH), 9.26 (s, 1H, NH), 8.29 (d, J = 1.9 Hz, 1H, H-11), 8.18 (d, J = 9.6 Hz, 1H, H-4), 8.13 (s, 4H, H-22, H-23, H-25, H-26), 7.69–7.54 (m, 3H, H-3, H-13, H-14), 7.04 (s, 1H, H-20), 6.75 (d, J = 2.7 Hz, 1H, H-1), 3.82 (s, 3H, OCH_3_). **^13^C NMR (75 MHz, DMSO-*d_6_*):** δ ppm 180.67 (Cq), 158.40 (Cq), 147.88 (Cq), 147.36 (Cq), 140.60, 139.87 (CH), 138.45, 138.11, 134.27, 131.52, 129.07 (CH), 128.58, 127.80, 126.36, 125.10, 124.85, 123.47 (CH), 122.35, 98.45 (CH), 55.72 (OCH_3_). **HPLC purity:** 98%. **LRMS E*xact Mass*** calcd for C_22_H_16_ClN_5_O_3_S: 465.066; *m*/*z* [M]^+^ found: 466 (100%); 467 (25%); 468 (42%).

#### 3.2.19. (E)-N-(6-chloro-2-methoxyacridin-9-yl)-2-(4-methylbenzylidene)hydrazine-1-carbothioamide (**DL-06**)

Orange solid; yield: 90%. MP: 157–158 °C. R_f_ 0.60 (*n*-hexane/ethyl acetate, 7:3). **^1^H NMR (300 MHz, DMSO-*d_6_*):** δ ppm 9.22 (s, 1H, NH), 9.11 (s, 1H, NH), 8.40 (d, J = 1.9 Hz, 1H, H-11), 8.30 (d, J = 9.5 Hz, 1H, H-4), 7.69 (m, 3H, H-3, H-13, H-14), 7.66 (d, J = 8.9 Hz, 2H, H-22, H-26), 7.12 (d, J = 8.0 Hz, 2H, H-23, H-25), 6.92 (s, 1H, H-20), 6.83 (d, J = 2.7 Hz, 1H, H-1), 3.83 (s, 3H, OCH_3_), 2.26 (s, 3H, CH_3_). **^13^C NMR (75 MHz, DMSO-*d_6_*):** δ ppm 13C NMR (75 MHz, DMSO) δ 180.14 (Cq), 158.59 (Cq), 145.14 (Cq), 144.99 (CH), 143.52 (Cq), 141.53 (Cq), 140.44 (Cq) 135.86 (Cq), 130.70 (CH), 129.05 (CH), 129.00, 128.14 (CH), 127.91, 125.77 (CH), 125.40, 122.94, 99.01 (CH), 55.88 (OCH_3_), 21.01 (CH_3_). **HPLC purity:** 98%. **LRMS E*xact Mass*** calcd for C_23_H_19_ClN_4_OS: 434.097; *m*/*z* [M]^+^ found: 435 (100%); 436 (26%); 437 (34%).

#### 3.2.20. (E)-N-(6-chloro-2-methoxyacridin-9-yl)-2-(4-hydroxybenzylidene)hydrazine-1-carbothioamide (**DL-07**)

Orange solid; yield: 82%. MP: 163–164 °C. R_f_ 0.40 (*n*-hexane/ethyl acetate, 6:4). **^1^H** **NMR (500 MHz, DMSO-*d_6_*):** δ ppm 9.92 (s, 1H, OH), 9.04 (s, 1H, NH), 8.96 (s, 1H, NH), 8.30 (d, J = 2.3 Hz, 1H, H-11), 8.19 (d, J = 9.5 Hz, 1H, H-4), 7.65–7.60 (m, 4H, H-3, H-13, H-22, H-26), 7.58 (d, J = 9.1 Hz, 1H, H-14), 6.76 (d, J = 2.7 Hz, 1H, H-1), 6.71 (s, 1H, H-20), 6.67 (d, J = 8.3 Hz, 2H, H-23, H-25), 3.82 (s, 3H, OCH_3_). **^13^C NMR (75 MHz, DMSO-*d_6_*):** δ ppm 180.54 (Cq), 160.17 (Cq), 158.70 (Cq), 150.14, 148.05 (Cq), 148.00 (Cq), 143.55 (CH), 139.32 (Cq), 134.58 (Cq), 132.20 (CH), 130.52 (CH), 128.85 (CH), 128.48 (CH), 126.65 (CH), 125.85 (Cq), 125.59 (CH), 124.85 (Cq), 123.10 (Cq), 115.75 (CH), 99.14 (CH), 56.15 (OCH_3_). **HPLC purity:** 99%. **LRMS E*xact Mass*** calcd for C_22_H_17_ClN_4_O_2_S: 436.076; *m*/*z* [M]^+^ found: 437 (100%); 438 (30%); 439 (45%).

#### 3.2.21. (E)-N-(6-chloro-2-methoxyacridin-9-yl)-2-(3-hydroxybenzylidene)hydrazine-1-carbothioamide (**DL-08**)

Rust orange solid; yield: 81%. MP: 155 °C. R_f_ 0.44 (*n*-hexane/ethyl acetate, 6:4). **^1^H NMR (500 MHz, DMSO-*d_6_*):** δ ppm 9.21 (s, 1H, NH), 9.04 (s, 1H, NH), 8.38 (d, J = 2.0 Hz, 1H, H-11), 8.28 (d, J = 9.4 Hz, 1H, H-4), 7.72 (dd, J = 9.0, 2.2 Hz, 2H, H-3, H-13), 7.65 (d, J = 9.2 Hz, 1H, H-14), 7.21–7.16 (m, 2H, H-22, H-26), 7.10 (t, J = 8.0 Hz, 1H, H-25), 6.85 (s, 1H, H-20), 6.84–6.77 (m, 2H, H-1, H-24), 3.84 (s, 3H, OCH_3_). **^13^C NMR (125 MHz, DMSO-*d_6_*):** 180.82 (Cq), 159.03 (Cq), 157.96 (Cq), 146.16 (Cq), 144.09 (CH), 135.90 (Cq), 135.12 (Cq), 130.29 (CH), 129.89 (CH), 129.34 (CH), 127.94 (CH), 126.64 (CH), 126.11 (Cq), 125.79 (CH), 123.32 (Cq), 119.75 (CH), 118.16 (CH), 114.98 (CH), 99.43 (CH), 56.33 (OCH_3_). **HPLC purity:** 98%. **LRMS E*xact Mass*** calcd for C_22_H_17_ClN_4_O_2_S: 436.076; *m*/*z* [M]^+^ found: 437 (100%); 438 (32%); 439 (42%).

#### 3.2.22. (E)-N-(6-chloro-2-methoxyacridin-9-yl)-2-(4-(dimethylamino)benzylidene)hydrazine-1-carbothioamide (**DL-09**)

Rust orange solid; yield: 89%. MP: 149–150 °C. R_f_ 0.52 (*n*-hexane/ethyl acetate, 7:3). **^1^H** **NMR (500 MHz, DMSO-*d_6_*):** δ ppm 9.00 (s, 1H, NH), 8.93 (s, 1H, NH), 8.30 (d, J = 2.0 Hz, 1H, H-11), 8.20 (d, J = 9.5 Hz, 1H, H-4), 7.69–7.63 (m, 2H, H-3, H-13), 7.61 (d, J = 9.1 Hz, 1H, H-14), 7.59 (d, J = 8.7 Hz, 2H, H-22, H-26), 6.79 (d, J = 2.8 Hz, 1H, H-1), 6.73 (s, 1H, H-20), 6.62 (d, J = 8.5 Hz, 2H, H-23, H-25), 3.82 (s, 3H, OCH_3_), 2.92 (s, 6H, N(CH_3_)_2_). **^13^C NMR (125 MHz, DMSO-*d_6_*):** δ ppm 180.30 (Cq), 158.76 (Cq), 151.98 (Cq), 147.54 (Cq), 147.45 (Cq), 144.24 (CH), 140.25 (Cq), 134.98 (Cq), 131.62 (CH), 130.04 (CH), 128.91 (CH), 127.93 (CH), 126.99 (CH), 126.01 (Cq), 125.79 (CH), 123.32 (Cq), 112.10 (CH), 99.40 (CH), 56.20 (OCH_3_), 40.44 (N(CH_3_)_2_). **HPLC purity:** 98%. **LRMS E*xact Mass*** calcd for C_24_H_22_ClN_5_OS: 463.123; *m*/*z* [M]^+^ found: 464 (100%); 465 (32%); 466 (44%).

#### 3.2.23. (E)-2-(4-bromobenzylidene)-N-(6-chloro-2-methoxyacridin-9-yl)hydrazine-1-carbothioamide (**DL-10**)

Orange solid; yield: 75%. MP: 162–163 °C. R_f_ 0.65 (*n*-hexane/ethyl acetate, 7:3). **^1^H NMR (500 MHz, DMSO-*d_6_*):** δ ppm 9.22 (s, 1H, NH), 9.16 (s, 1H, NH), 8.35 (d, J = 2.0 Hz, 1H, H-11), 8.24 (d, J = 9.5 Hz, 1H, H-4), 7.79 (d, J = 8.7 Hz, 2H, H-22, H-26), 7.67 (td, J = 8.0, 4.4 Hz, 2H, H-3, H-13), 7.61 (d, J = 9.2 Hz, 1H, H-14), 7.51 (d, J = 8.6 Hz, 2H, H-23, H-25), 6.88 (s, 1H, H-20), 6.77 (d, J = 2.8 Hz, 1H, H-1), 3.83 (s, 3H, OCH_3_). **^13^C NMR (125 MHz, DMSO-*d_6_*):** δ ppm 180.94 (Cq), 158.92 (Cq), 147.25 (Cq), 142.44 (CH), 139.96 (Cq), 135.22 (Cq), 133.28 (Cq), 131.88 (CH), 131.35 (CH), 130.53 (CH), 129.15 (CH), 127.66 (CH), 127.28 (CH), 125.86 (Cq), 125.59 (CH), 124.28 (Cq), 123.11 (Cq), 99.21 (CH), 56.26 (OCH_3_). **HPLC purity:** 99%. **LRMS E*xact Mass*** calcd for C_22_H_16_BrClN_4_OS: 497.992; *m*/*z* [M]^+^ found: 499 (100%); 501 (100%).

### 3.3. Cytotoxic Activity

#### 3.3.1. Cells

The human cancer cell lines HCT116 (colon carcinoma) and HepG2 (hepatocellular carcinoma), the mouse cancer cell line B16-F10 (melanoma), and the non-cancerous human cell line MRC-5 (lung fibroblast) were obtained from the American Type Culture Collection (ATCC, Manassas, VA, USA) and cultured according to the ATCC animal cell culture guide. All cell lines tested negative for mycoplasma using a mycoplasma stain kit.

#### 3.3.2. Cytotoxicity Assay

Cell viability was measured using the Alamar blue assay, as previously described [67,96,97]. Cells were seeded in 96-well plates for all experiments. The compounds were dissolved in dimethyl sulfoxide (DMSO, Sigma-Aldrich) and added to each well in eight serial concentrations ranging from 0.19 to 25 μg/mL. As a positive control, doxorubicin was used. After 72 h of treatment, 20 μL of resazurin (0.312 mg/mL) was added to each well. A SpectraMax 190 Micro-plate Reader was used to measure absorbances at 570 and 595 nm (Molecular Devices, Sunnyvale, CA, USA). Nonlinear regression with 95% confidence intervals (CI 95%) was used to calculate the inhibitory concentration of 50% (IC_50_) using GraphPad Prism software (Intuitive Software for Science; San Diego, CA, USA).

### 3.4. Interaction with DNA

#### 3.4.1. UV–Visible Spectroscopy

The concentration of the calf thymus, Type I, fibers (CT DNA), was determined based on the extinction coefficient of the absorbance at 260 nm (6600 L mol^−1^ cm^−1^) [98]. All compounds were dissolved in methanol (HPLC grade) at a concentration of 1 mmol L^−1^ (stock solution). Working solutions were prepared by diluting the stock solution using Tris-HCl buffer (pH 7.4), with concentrations of 40 µmol L^−1^. Under the optimized concentration, the compounds were titered with increasing concentrations of CT DNA (5 to 60 µmol L^−1^). After shaking the system and incubating at room temperature for 10 min, the measurements were recorded using a JASCO J818 spectropolarimeter in the range of 220 to 500 nm at 298 K. The intrinsic bond constant (Kb) was obtained by adjusting the data according to the equation below [99]:

[DNA]/(Ɛ_a_ − Ɛ_f_) = [DNA]/(Ɛ_b_ − Ɛ_f_) + 1/[Kb (Ɛ_b_ − Ɛ_f_)]

where Ɛ_a_, Ɛ_b_ and Ɛ_f_ are the apparent extinction coefficient of the compounds, the extinction coefficient of the compounds found in their free form and the extinction coefficient of the compounds when fully DNA-bound, respectively. The graph of [DNA]/(Ɛ_a_ − Ɛ_f_) versus [DNA] was used to obtain the Kb from the ratio between the slope and the intercept, as previously described in the literature [77,79,86]. Binding data were obtained using SigmaPlot 10.0 software. The study of possible DNA denaturation using methanol–water solutions was evaluated up to the maximum final concentration of methanol in the assays (6%) (ESI, Figures S50 and S53).

#### 3.4.2. Circular Dichroism

The circular dichroism (CD) spectra of DNA in the presence of the compounds were recorded using a fixed concentration of DNA at 60 µmol L^−1^ and increasing concentrations of the compounds (from 0 to 60 µmol L^−1^) (ESI, Figures S54–S57). Readings were performed in the range from 215 to 400 nm, with a speed of 200 nm/min and a band of 5 nm. The entire study was monitored by the high voltage (HT) signal; this parameter indicates the reliability of the generated DC spectra, limiting the HT signal up to 700 volts, indicated by JASCO [85,86,100]. Binding data were obtained using SigmaPlot 10.0 software.

#### 3.4.3. Fluorescence Competition with EtBr

The fluorescence titration results were analyzed using the Stern–Volmer equation [101], and the Ksv values of CT DNA–EtBr in the presence of the compounds calculated the slope of the graphs. The fluorescence spectra of the EtBr or CT DNA–EtBr compounds were obtained by excitation at 526 nm and scanning the emission from 530 to 650 nm. Solutions containing CT DNA–EtBr (20 μM:100 μM) were analyzed in the presence of several derivative concentrations (10, 20, 40, 60, 80 and 100 μM) in Tris-HCl buffer (0.1 M, pH 7.4) (ESI, Figures S58 and S59):

F_0_/F = 1 + Ksv[Q]

F_0_ and F represent the steady-state fluorescence intensities of CT DNA–EtBr in the absence and presence of the compounds, respectively. [Q] refers to the concentration of derivatives as a quencher. Ksv indicates the Stern–Volmer quenching constant [78,80].

### 3.5. Human Topoisomerase IIα Inhibition Assay

For testing the ability of Topo IIα inhibition, the compounds were evaluated in the DNA plasmid relaxation assay. This test consists of evaluating the Topo IIα enzyme activity in the absence and presence of the compound, followed by electrophoresis analysis, as previously described by Almeida et al. (2016) [38]. Briefly, 100 ng pUC19 DNA plasmid (Sigma-Aldrich) and 4.0 units of recombinant human (p170) topoisomerase II (Sigma-Aldrich) in relaxation buffer without or with tested compounds were incubated for 30 min at 37 °C. The tested concentrations were 100 mM, and amsacrine (mAMSA) was used as a positive control. Reactions were terminated with 1% SDS and digested with proteinase K. Then, the reaction products were analyzed by electrophoresis gel stained with ethidium bromide and photographed under UV light. Quantitative evaluation (% of enzyme inhibition by compounds about the positive control mAMSA) of the gel bands corresponding to supercoiled DNA was performed by densitometry using image-processing software (Scion Image, version Beta 4.0.2.) according to Gouveia et al. (2018) [68].

### 3.6. Molecular Docking

The studied structures were first treated based on the semi-empirical theory at the PM6 level using the Spartan 14 software. After being optimized, they were submitted to the study of molecular docking through the Gold 5.8.1 program. The structure of human topoisomerase IIα in complex with DNA (PDB ID: 5gwk) was used as a DNA–Topo IIα model [70]. To validate the study, redocking for target was performed for etoposide in complex with a DNA–Topo IIα, giving an RMSD = 0.48. Receptors and ligand files were studied, the receptor was treated as a rigid molecule, while ligands were treated as flexible. The docking results were analyzed, and intermolecular interactions were used as parameters to measure affinity energy with the target (score). The conformer with the highest score was investigated by the Pymol 2.3.2 program; distance and type of interaction with the biomacromolecule were preponderant for the completion of the modeling study [71,86].

### 3.7. Evaluation of the Non-Clinical Toxicity

#### 3.7.1. Acute Toxicity Study (14 Days)

An acute toxicity study was conducted according to Organization for Economic Co-operation and Development (OECD) guideline 423 (OECD 2001) with modifications (National Health Surveillance Agency Brazil, 2013) [102,103]. In principle, the method is not intended to allow an accurate calculation of the lethal dose (LD_50_), despite providing an estimate of its value; however, it allows a classification of the substance into categories according to the Globally Harmonized Classification System (GHS). The tests were carried out with the consent of the ethics council of the State University of Paraiba (process no. 008/2021, approved 9 November 2021). Swiss mice, three females per group, were subjected to single oral doses of 2000 mg/kg of acridine–thiosemicarbazone derivatives, and to the control group, we administered only the vehicle (12% Tween 80 solution in saline). To trace possible behavioral changes, suggestive of activity in the central nervous system (CNS) or autonomic nervous system (ANS), after administration of the substance, careful observation was performed to detect possible clinical signs of toxicity (e.g., general appearance, ataxia, vocal tremor, irritability, body tone, tremor, salivation, tearing, eyelid ptosis, seizures, behavioral changes and abnormal movements) and animal mortality at the intervals 0, 15, 30 and 60 min, after 4 h and daily for 14 days [88,89,104].

#### 3.7.2. Necropsy, Macroscopic Analysis and Organ Weight

After the end of treatment with acridine–thiosemicarbazone derivatives, the animals were anesthetized with a xylazine–ketamine solution of 0.2 mL/100 g (8.75 mL ketamine (100 mg/mL) and 1.25 mL xylazine (10 mg/mL)) as per Cornell University/Cornell Center for Animal Resources and Education protocol described by Flecknell (1996) [105]. After euthanasia, a macroscopic assessment of the external body surface, cavities (thoracic, abdominal and cranial) and organs (change in position, shape, size, color and consistency) was carried out. The absolute and relative weights of the brain, lungs, liver, heart, spleen and kidneys were also determined. Relative weight was calculated using the formula: relative organ weight (%) = (weight of organ/weight of mouse) × 100 [88,89].

### 3.8. Cytotoxicity Assay on Leukemic Cells

#### 3.8.1. Cells

K562 (human erythroleukemic) and its multidrug-resistant counterpart, K562-Lucena 1, were obtained from the Tumor Immunology Laboratory (Federal University of Rio de Janeiro) (Rumjanek et al., 2001) and were cultured as recommended by Daflon-Yunes et al., 2013 [93,106,107].

#### 3.8.2. Cytotoxicity Assay

CL-07, DL-01 and DL-08 compounds were evaluated for cell viability on K562 and K562-Lucena 1 cells using the colorimetric tetrazolium method (Mosmann, 1983) [108]. Cells were plated in 96-well plates. Compounds were dissolved in 1% (*v*/*v*) DMSO and RPMI-1640 medium (Sigma-Aldrich) in five serial concentrations from 1 to 50 µg/mL and were later added to each well and incubated for 72 h at 37 °C in a 5% CO_2_ humidified environment. As a positive control, 1 µM staurosporine was used. Then, the cells were treated with 20 µL of 5 mg/mL 3-(4,5-dimethylthiazol-2yl)-2,5-diphenyltetrazolium bromide solution (MTT solution in phosphate buffer solution) for 3 h. At the end of treatment, absorbances at 562 nm were measured using an iMark™ Microplate Absorbance Reader (iMark™, Hercules, CA, USA). Half-inhibitory concentration (IC_50_) was obtained by nonlinear regression with 95% confidence intervals (CI 95%) using GraphPad Prism software (Intuitive Software for Science; San Diego, CA, USA).

## 4. Conclusions

In summary, twenty new acridine–thiosemicarbazone derivatives were synthesized and their chemical structures were successfully confirmed. The new derivatives showed moderate cytotoxic activity with low selectivity for tumor cells. Additionally, some derivatives were evaluated for their ability to inhibit Topo IIα at 100 µM. DL-01, DL-07 and DL-08 were able to inhibit enzymatic activity with percentages above 74% when compared to mAMSA activity. DL-06 and CL-07 did not show inhibition capacity against Topo IIα. Molecular docking studies showed that stereoelectronic restrictions of the enzymatic cavity directed to the substituted portion of the benzylidene of these derivatives, as well as alterations in the electronic profile of the acridine scaffold, would justify the non-inhibitory profile presented. Three of the most cytotoxic compounds, CL-07, DL-01 and DL-08, were studied for their potential to interact with DNA, and their cytotoxicity on leukemic cells and non-clinical toxicity and cytotoxicity assays on leukemic cells. Compound CL-07 showed the highest affinity for DNA, reaching the highest value found for hypochromism and binding constant (Kb) by UV–Vis spectroscopy. CL-07 also had more accentuated changes in the DC of CT DNA and the highest fluorescence quenching constant (Ksv) against the CT DNA–EtBr system compared to other compounds studied. In general, the compounds studied did not show toxicity at the concentration of 2000 mg/kg in mice, and the cytotoxicity analysis against the leukemic cells studied indicated an active profile of the DL derivatives. Together, these results suggest that topoisomerase is a more effective mechanism for this class of compounds, with DNA as a secondary mechanism. The substitution patterns studied were intrinsic factors for the antiproliferative activities found, as well as in the Topo IIα inhibition profile and DNA affinity. Tests on other cancer cell lines may be justified by the high inhibition found for DNA–Topo IIα [50]. In this work, we based our development on some general concepts of the Topliss tree for aromatic substitutions. Given our results, new compounds with substitutions in R such as 4-F and 2-OH are being developed. These compounds may present increases in potency against the cell lines and are being studied biologically [109,110].

## Figures and Tables

**Table 1:** Cytotoxicity activity of new acridine–thiosemicarbazone derivatives.

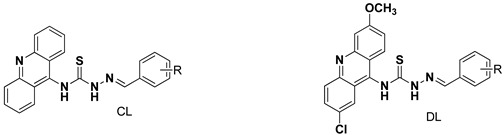					
R	Comp	IC_50_ (µM) ^1^			
		HCT116	HepG2	B16-F10	MRC-5
**H**	CL-01	>70.00	>70.00	ND	>70.00
**4-Cl**	CL-02	>70.00	>70.00	ND	>70.00
**4-OCH_3_**	CL-03	>70.00	>70.00	>70.00	>70.00
**2,4-Cl**	CL-04	>60.00	>60.00	ND	>60.00
**4-NO_2_**	CL-05	>60.00	>60.00	**22.71**	**26.78**
				16.60–31.09	22.26–32.19
**4-CH_3_**	CL-06	**64.76**	**58.06**	**23.54**	**28.02**
		44.07–95.16	38.93–86.62	15.94–28.50	21.59–36.31
**4-OH**	CL-07	**39.67**	**50.66**	**19.83**	**43.40**
		19.13–63.40	43.24–59.31	18.73–29.61	36.28–51.89
**3-OH**	CL-08	**39.16**	**49.42**	**21.32**	**33.38**
		17.76–59.53	43.99–55.52	14.56–27.00	27.79–40.07
**4-N(CH_3_)_2_**	CL-09	>60.00	>60.00	ND	>60.00
**4-Br**	CL-10	>60.00	>60.00	ND	>60.00
**H**	DL-01	>60.00	**21.87**	**39.56**	**36.97**
			15.97–29.97	24.11–64.89	25.35–53.97
**4-Cl**	DL-02	>60.00	>60.00	**30.57**	>60.00
				21.74–42.99	
**4-OCH_3_**	DL-03	>60.00	>60.00	>60.00	>60.00
**2,4-Cl**	DL-04	>60.00	>60.00	>60.00	>60.00
**4-NO_2_**	DL-05	>60.00	>60.00	>60.00	>60.00
**4-CH_3_**	DL-06	>60.00	**33.38**	**26.26**	>60.00
			23.64–47.13	14.81–46.53	
**4-OH**	DL-07	**28.46**	**24.84**	**21.12**	**23.64**
		25.13–32.24	16.21–38.04	16.39–26.72	17.06–32.79
**3-OH**	DL-08	**24.22**	**21.28**	**14.79**	**14.36**
		21.32–27.49	11.17–40.50	11.51–18.99	9.61–21.44
**4-N(CH_3_)_2_**	DL-09	>60.00	>60.00	**46.04**	>60.00
				27.68–50.85	
**4-Br**	DL-10	>50.00	**20.88**	**34.62**	>50.00
			11.22–38.83	25.74–53.55	
	DOX ^2^	**0.15**	**0.15**	**0.03**	**2.22**
	5-FU ^3^	**4.1**	**1.3**	**3.5**	**57.9**

^1^ Data are presented as IC_50_ values in µM and their 95% confidence interval was obtained by nonlinear regression from three independent experiments performed in duplicate, measured using Alamar blue assay after 72 h incubation. Cancer cells: HCT116 (human colon carcinoma), HepG2 (human hepatocellular carcinoma) and B16F10 (murine melanoma carcinoma). Non-cancerous cell: MRC-5 (human lung fibroblast). ^2^ Doxorubicin was used as a positive control. ^3^ Cytotoxicity for 5-fluorouracil (5-FU) was published by Silva et al. (2018) [67]. ND: not determined due to technical problems.

**Table 2:** Topo IIα inhibition activity of acridine derivatives.

Compounds	*m*AMSA	DL01	DL06	DL07	DL08	CL07
% of enzyme inhibition	100	77	-	74	79	-

**Table 3:** Docking scores and interacting residues of test compounds with DNA–Topo IIα.

Compounds	Docking Score	Hydrogen Bond	Hydrophobic Interactions	
			π-Alkyl	Stacked π–π
**CL-07**	82.02	T^9a^ G^10^ *	Arg^487^	A^12^ G^13^
**DL-01**	93.88	Arg^487^	T^9^ Arg^487^	G^13^
**DL-06**	84.01	-	T^9^ Arg^487^	A^12^ G^13^
**DL-07**	97.98	Arg^487^ T^9a^ Ser^464^ G^10^ *	T^9^ Arg^487^	A^12^ G^13^
**DL-08**	98.06	Arg^487^ Asp^463^ Ser^464^	T^9^ Arg^487^	T^9^ G^13^
***m*AMSA**	95.21	Asp^463^ Ser^464^ Arg^487^	Arg^487^	C^8^ T^9^ G^13^
**Etoposide**	98.35	Asp^463^	T^9^ Arg^487^	G^13^ Arg^487^

* Fragments of DNA strands.

**Table 4:** Absorptive properties of derivatives and EtBr in the presence of CT DNA.

Compounds	Absorptive Properties				
	λ Kb (nm)	∆λ	R^2^	Hypochromic (%)	Kb (10^4^) (M^−1^)
**CL-07**	325	3 ^1^	0.9586	25.52	4.51 ± 0.14
**DL-01**	320	2 ^2^	0.9626	12.89	0.75 ± 0.07
**DL-08**	305	3 ^2^	0.9708	14.79	1.73 ± 0.23
**EtBr**	489	6 ^2^	0.9652	35.15	5.56 ± 0.19

^1.^ Hypsochromic shift; ^2^ Batochromic shift.

**Table 5:** Emissive properties of derivatives in the presence of CT DNA–EtBr.

Compounds	Emissive Properties		
	R^2^	Quenching (%)	Ksv (10^3^) (M^−1^)
**CL-07**	0.9903	21.71	2.6
**DL-01**	0.9954	15.61	1.8
**DL-08**	0.9886	20.83	2.5

Emissive properties of compounds in the presence of CT DNA–EtBr from the emission quenching curves of EtBr–DNA: [EtBr] = 20 μmol L^−1^, [DNA] = 100 μmol L^−1^, [compound] = 0–100 μmol L^−1^.

**Table 6:** Effects of acridines derivatives in animals submitted to acute toxicity study.

Toxicity Study	DA/TA	Mortality (%)	Signs of Toxicity
**CL-07**	0/3	0	None
**DL-01**	0/3	0	Reduced feed consumption
**DL-08**	0/3	0	Hepatic steatosis
**NC**	0/3	0	None

NC, control vehicle; DA, dead animals; TA, total animals.

## Data Availability

Data is contained within the article and Supplementary Material.

## Supplementary Materials

The following supporting information can be downloaded at: https://www.mdpi.com/article/10.3390/ph15091098/s1.
