# Supplementary material for: Synthesis and Evaluation of Antiproliferative Activity, Topoisomerase IIα Inhibition, DNA Binding and Non-Clinical Toxicity of New Acridine–Thiosemicarbazone Derivatives
Source: Pharmaceuticals (Basel). 2022 Sep 2;15(9):1098. doi: 10.3390/ph15091098 (PMC9506480; doi:10.3390/ph15091098)
Supplement: Supplementary file 1 [file pharmaceuticals-15-01098-s001.zip › pharmaceuticals-1829120-supplementary.pdf]

Synthesis and Evaluation of Antiproliferative Activity, Topoisomerase IIα Inhibition, DNA Binding and Non-Clinical Toxicity of New Acridine–Thiosemicarbazone Derivatives.

Gleyton Sousa [1,2], Maria C. F. de Almeida [3], Lucas L. Lócio [3], Vanda L. dos Santos [3], Daniel P. Bezerra [4], Valdenizia R. Silva [4], Sinara M. V. de Almeida [5], Alice Simon [6], Thiago da S. Honório [6], Lucio M. Cabral [6], Rosane N. Castro [1], Ricardo O. de Moura [3] and Arthur E. Kümmerle [1,*]

[1] Institute of Chemistry, Federal Rural University of Rio de Janeiro, Seropédica 23897-000, Brazil

[2] Department of Chemistry, State University of Paraíba, Campina Grande 58429-500, Brazil

[3] Department of Biological Sciences, State University of Paraíba, Campina Grande 58429-500, Brazil

[4] Laboratory of Tissue Engineering and Immunopharmacology (LETI), Institute Gonçalo Moniz (IGM), Foundation Oswaldo Cruz (Fiocruz), Salvador 40296-710, Brazil

[5] Molecular Biology Laboratory, University of Pernambuco (UPE), Garanhuns 55290-000, Brazil

[6] Department of Drugs and Pharmaceutics, Faculty of Pharmacy, Universidade Federal do Rio de Janeiro, Av. Carlos Chagas Filho, 373, CCS, Bss, Rio de Janeiro 21941-902, Brazil

* Correspondence: akummerle@hotmail.com; Tel.: +55-21-998576298

SUPPLEMENTARY MATERIAL

(E)-N-(acridin-9-yl)-2-benzylidenehydrazine-1-carbothioamide **(CL-01)**

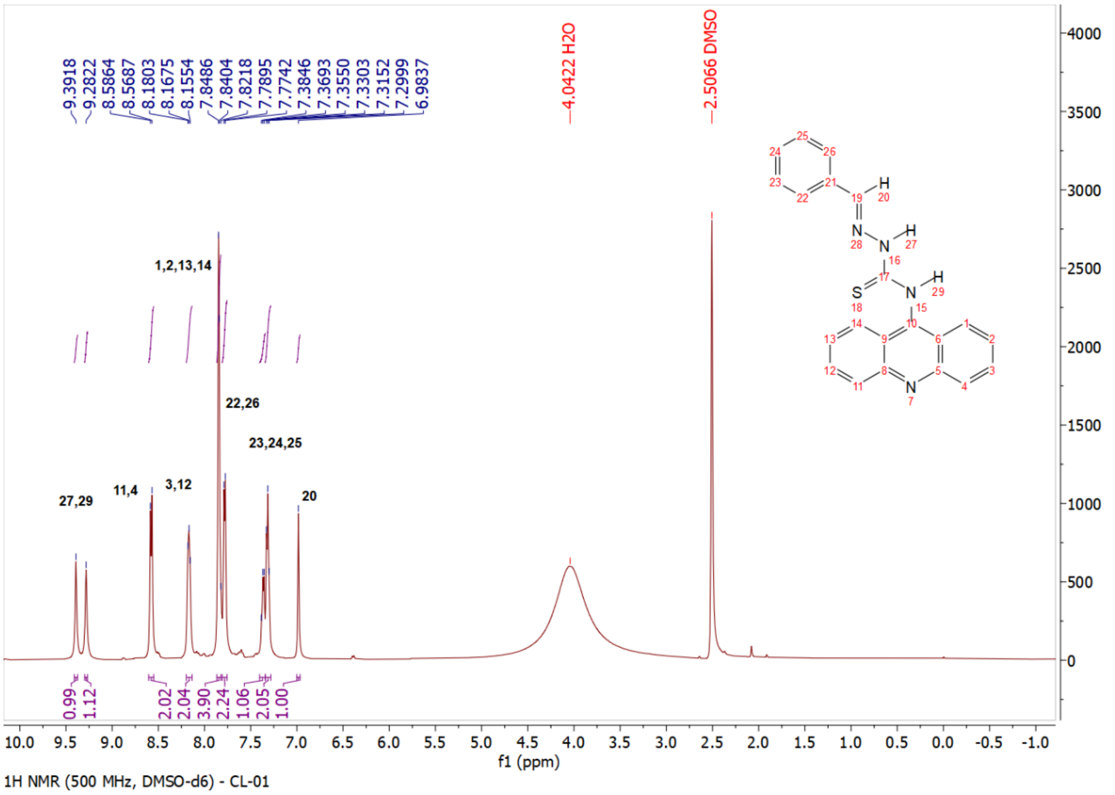

Figure S1. ^{1}H NMR spectrum of **CL-01**.

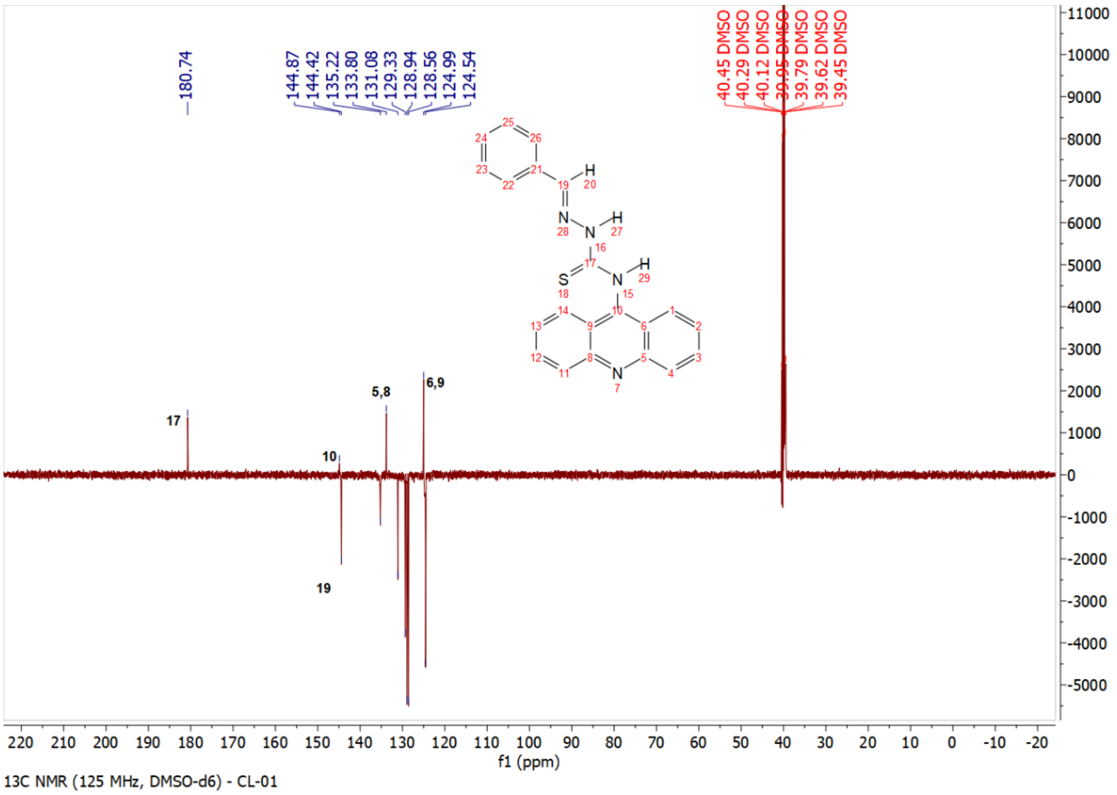

Figure S2. ^{13}C NMR spectrum of **CL-01**.

(E)-N-(acridin-9-yl)-2-(4-chlorobenzylidene)hydrazine-1-carbothioamide (**CL-02**)

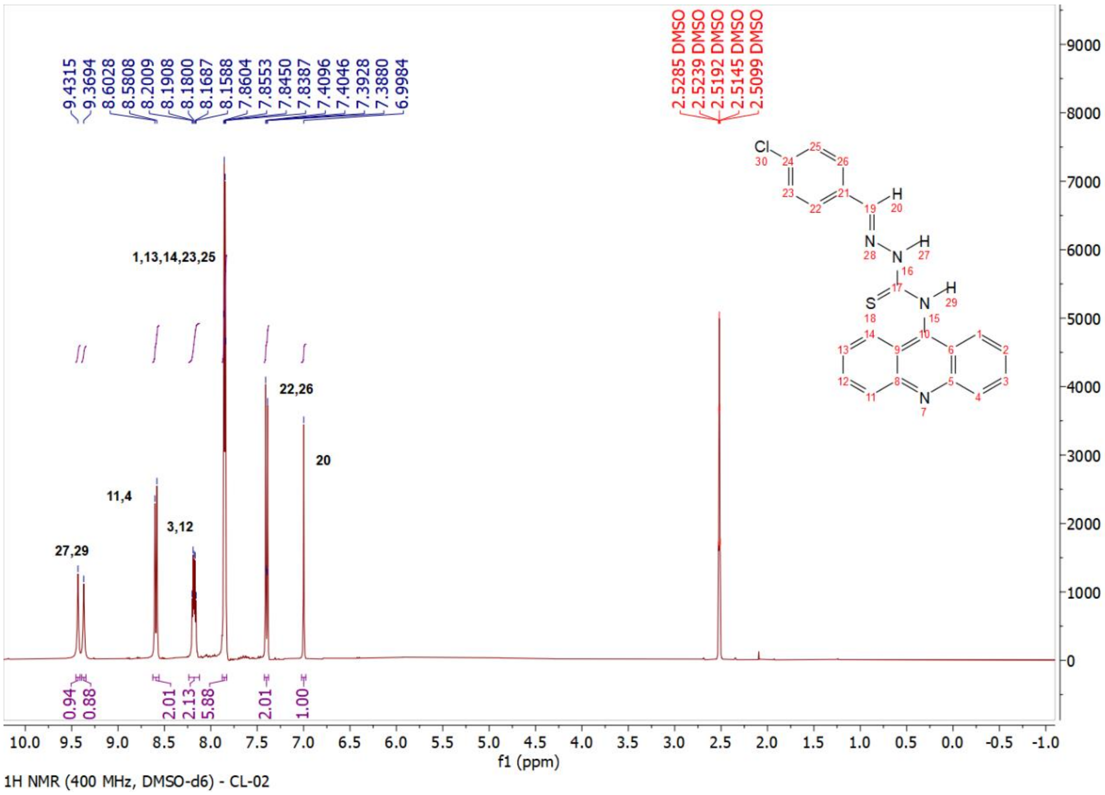

Figure S3. ^{1}H NMR spectrum of **CL-02**.

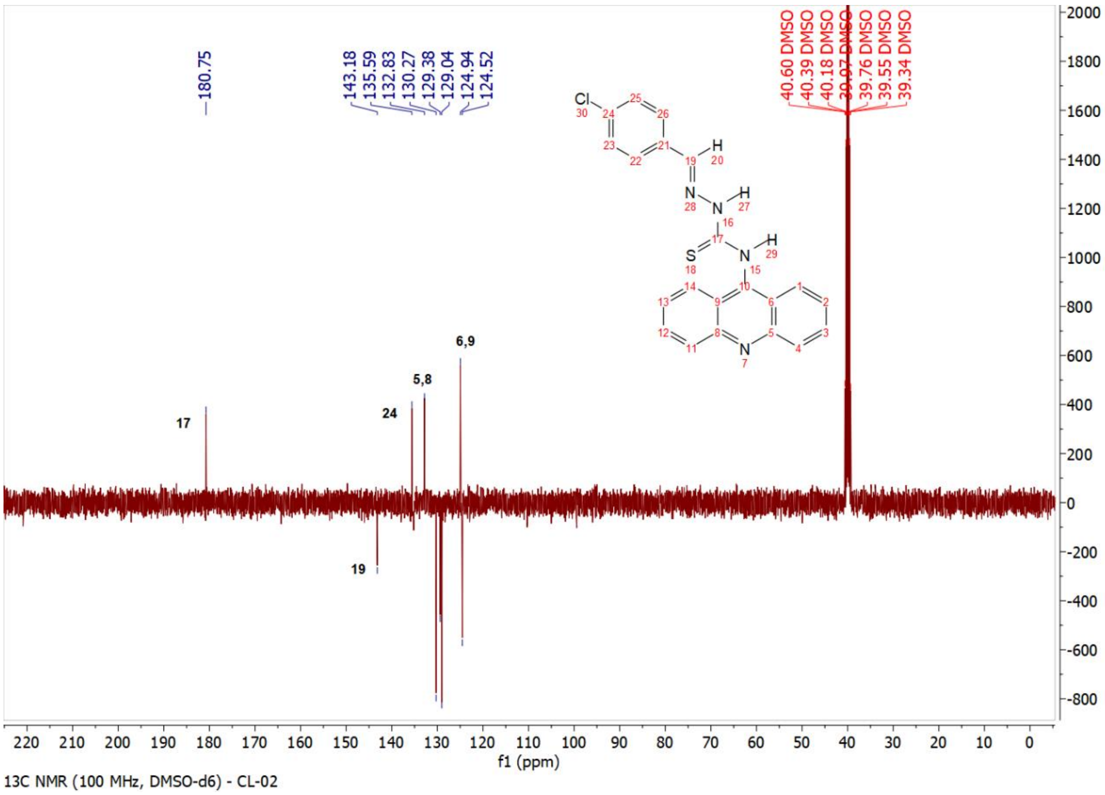

Figure S4. ^{13}C NMR spectrum of **CL-02**.

(E)-N-(acridin-9-yl)-2-(4-methoxybenzylidene)hydrazine-1-carbothioamide (**CL-03**)

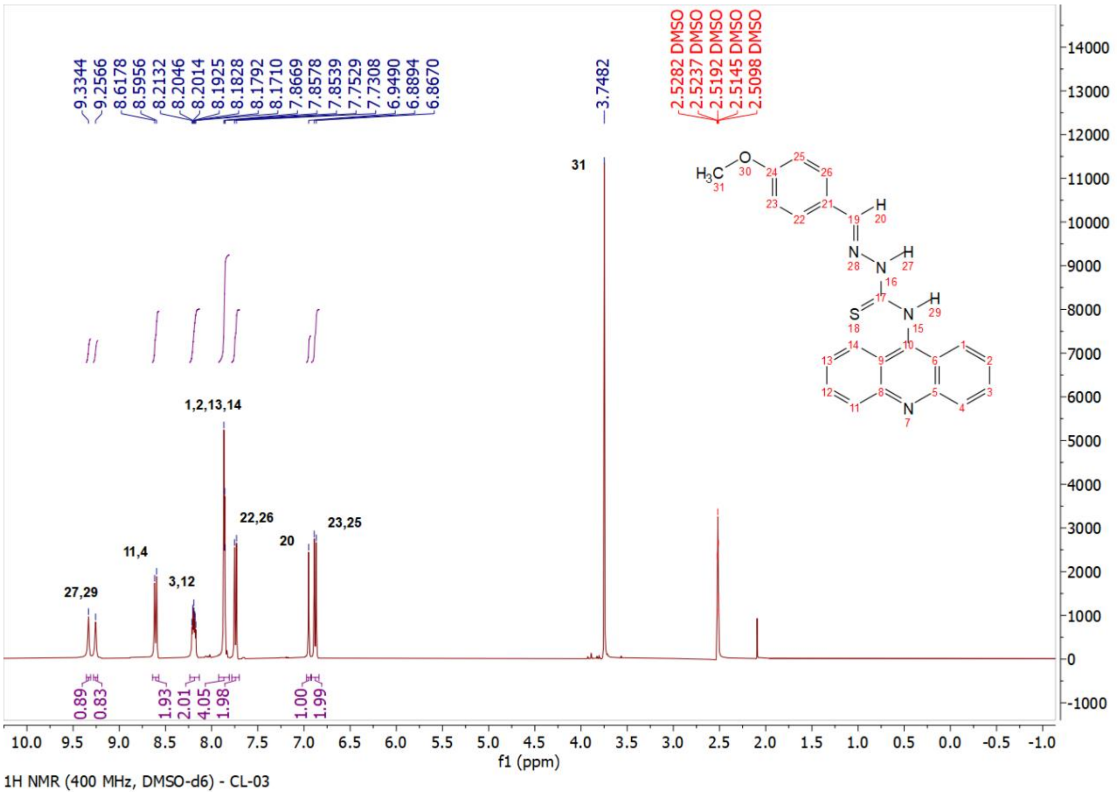

Figure S5. ^{1}H NMR spectrum of **CL-03**.

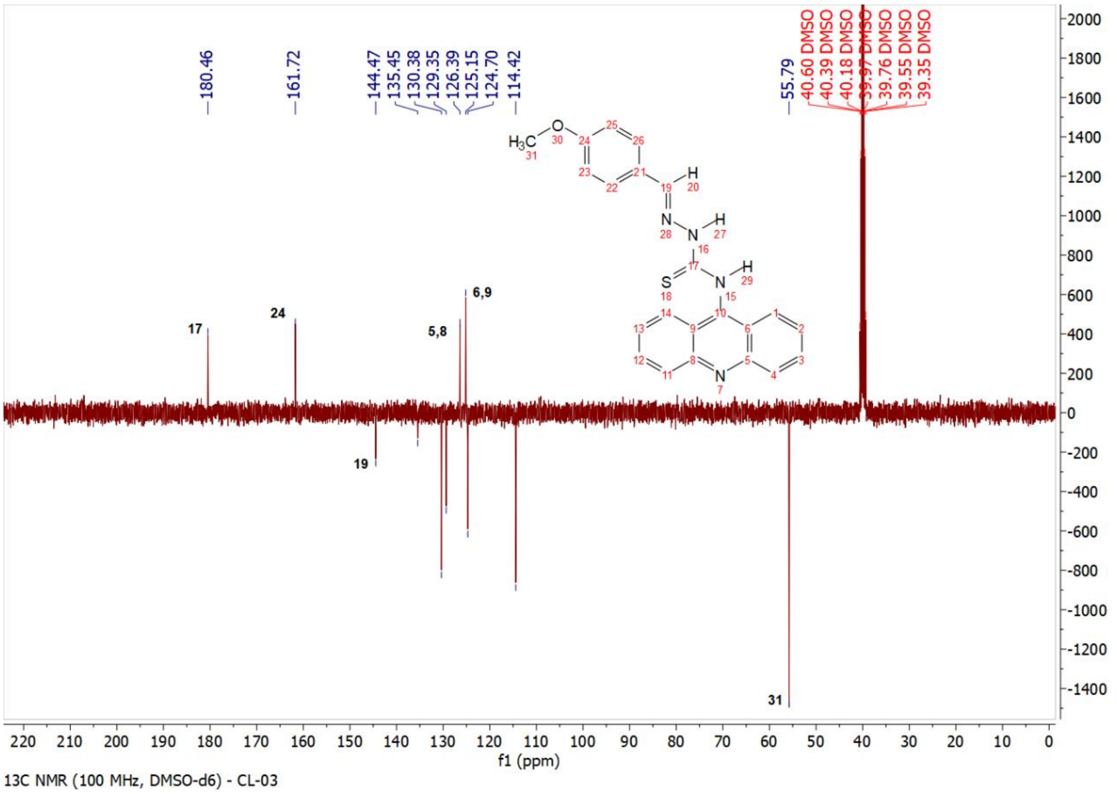

Figure S6. ^{13}C NMR spectrum of **CL-03**.

(E)-N-(acridin-9-yl)-2-(2,4-dichlorobenzylidene)hydrazine-1-carbothioamide (**CL-04**)

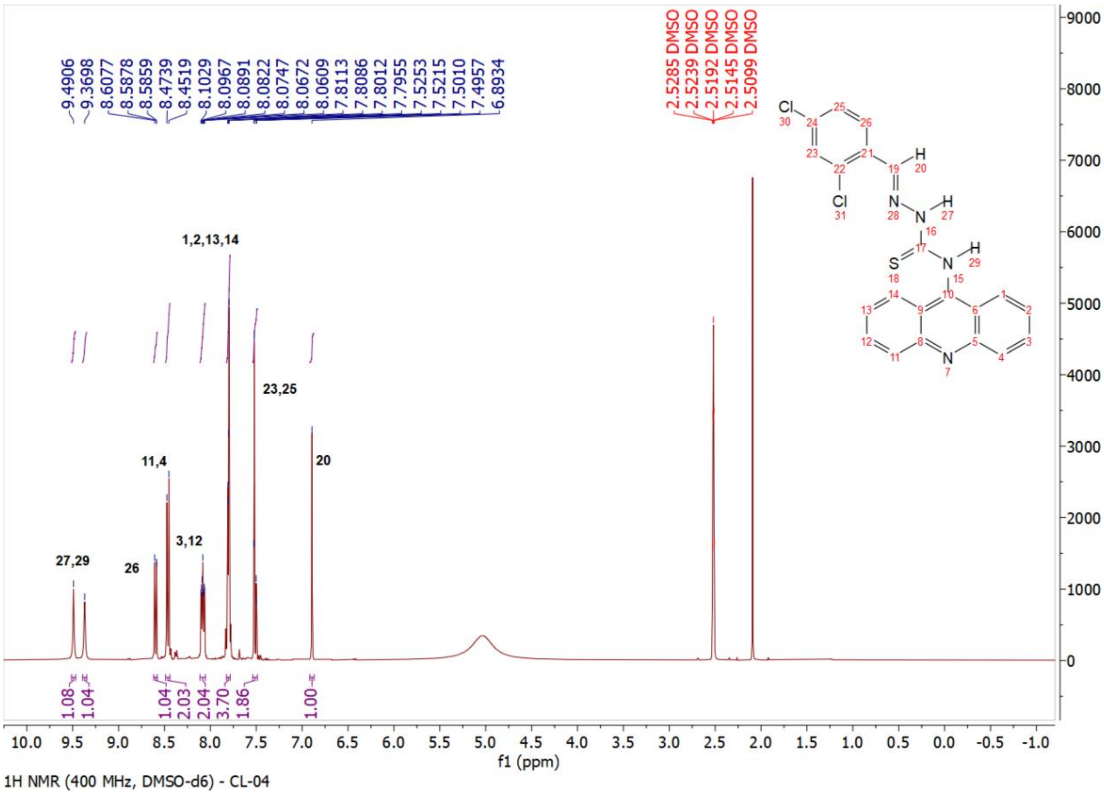

Figure S7. ^{1}H NMR spectrum of **CL-04**.

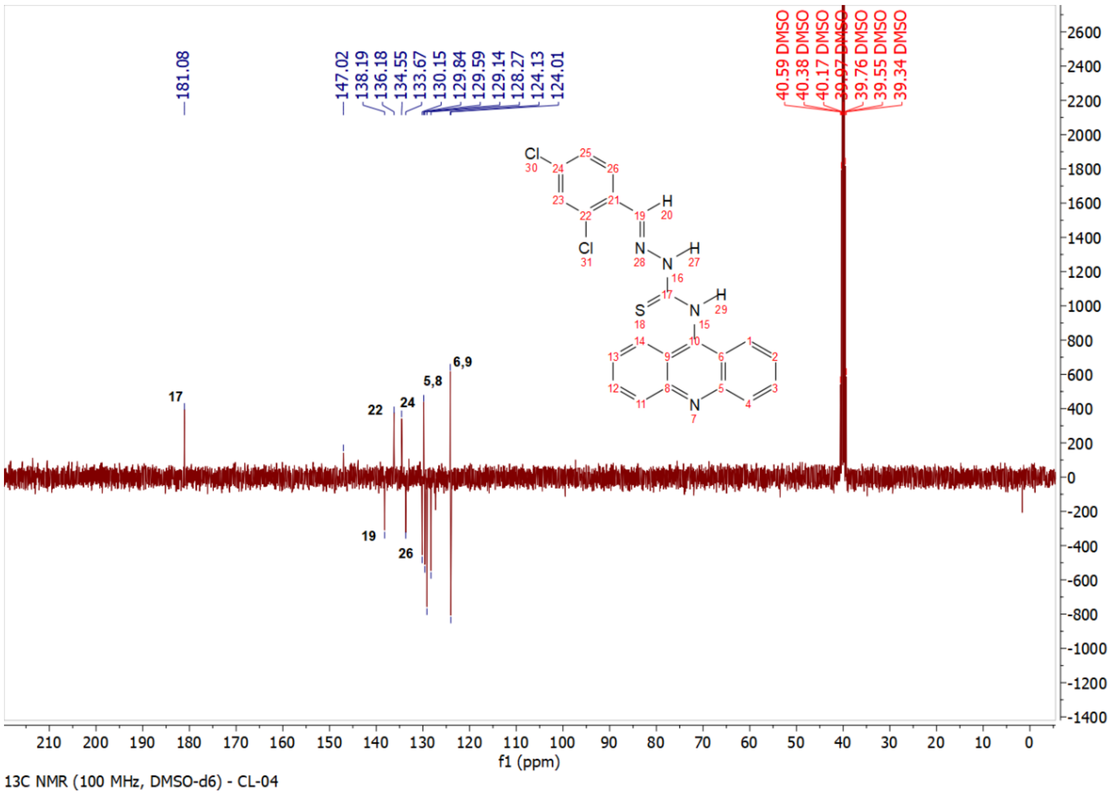

Figure S8. ^{13}C NMR spectrum of **CL-04**.

(E)-N-(acridin-9-yl)-2-(4-nitrobenzylidene)hydrazine-1-carbothioamide (**CL-05**)

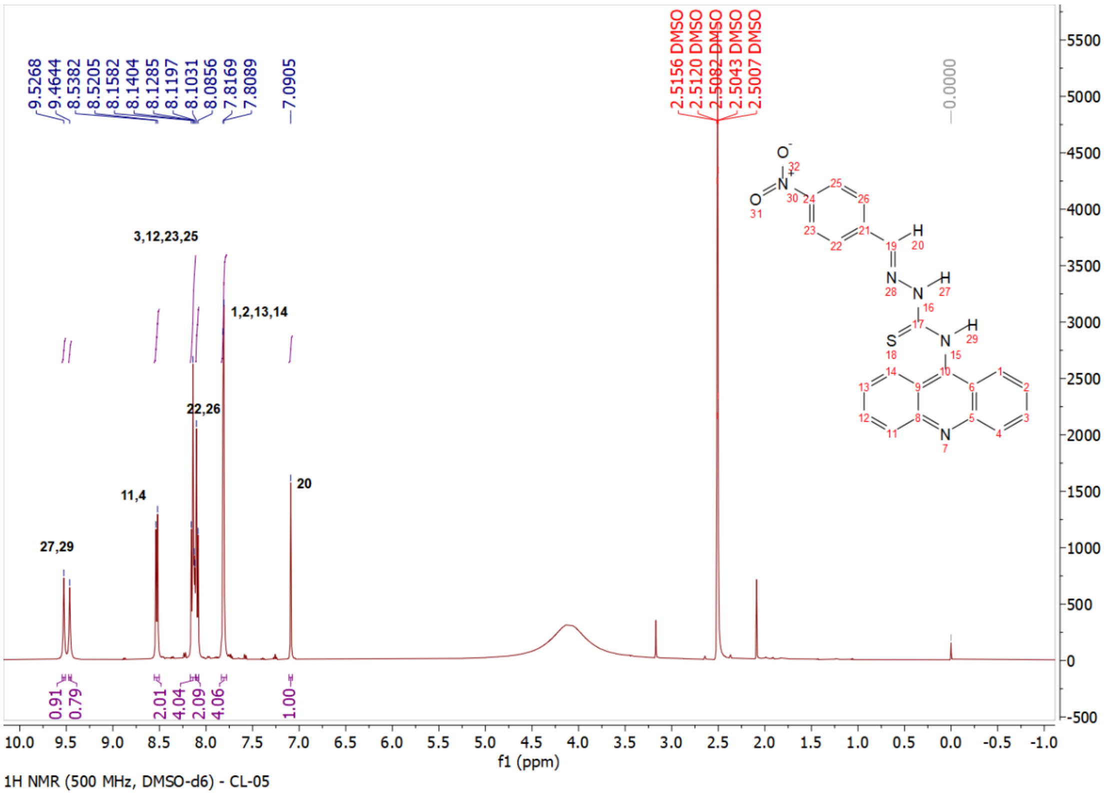

Figure S9. ^{1}H NMR spectrum of **CL-05**.

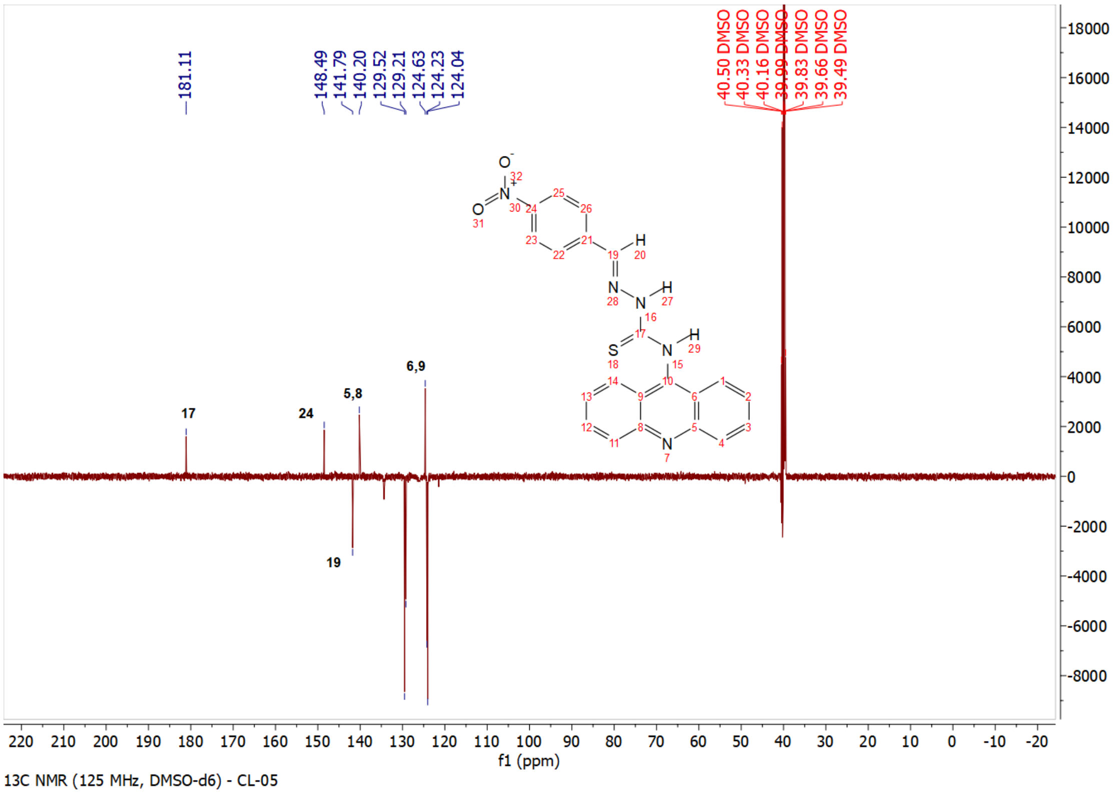

Figure S10: ^{13}C NMR spectrum of **CL-05**.

(E)-N-(acridin-9-yl)-2-(4-methylbenzylidene)hydrazine-1-carbothioamide (**CL-06**)

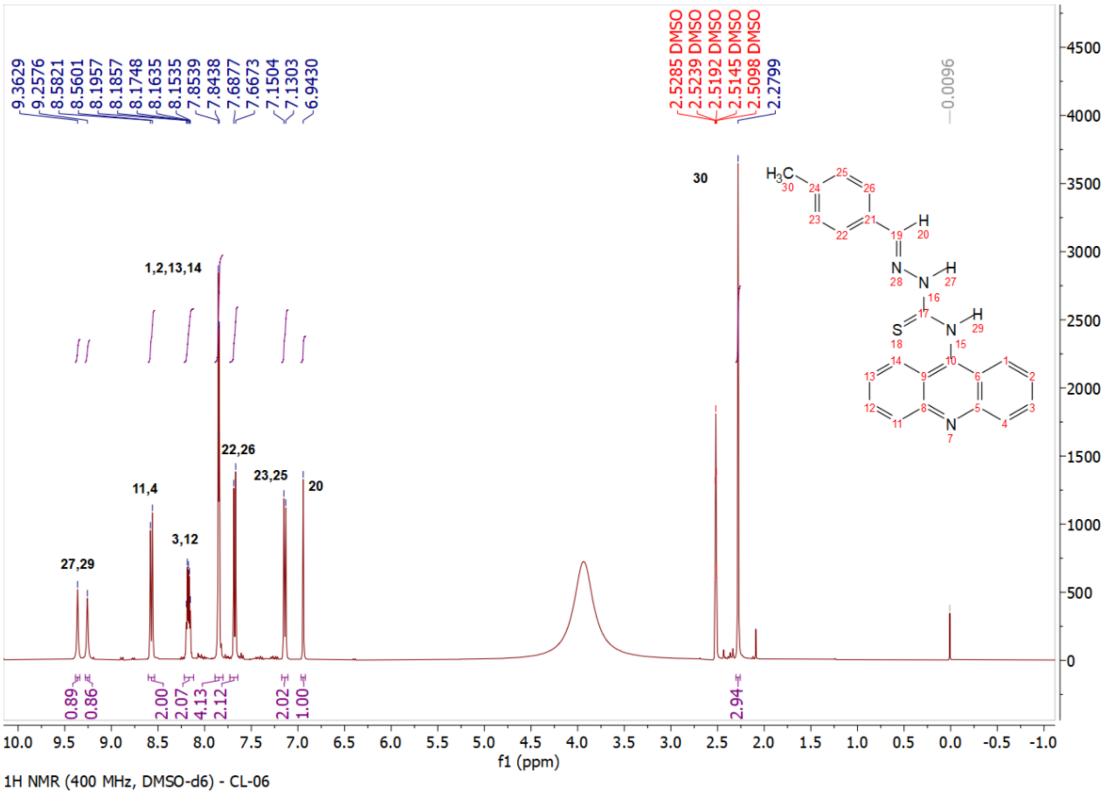

Figure S11. ^{1}H NMR spectrum of **CL-06**.

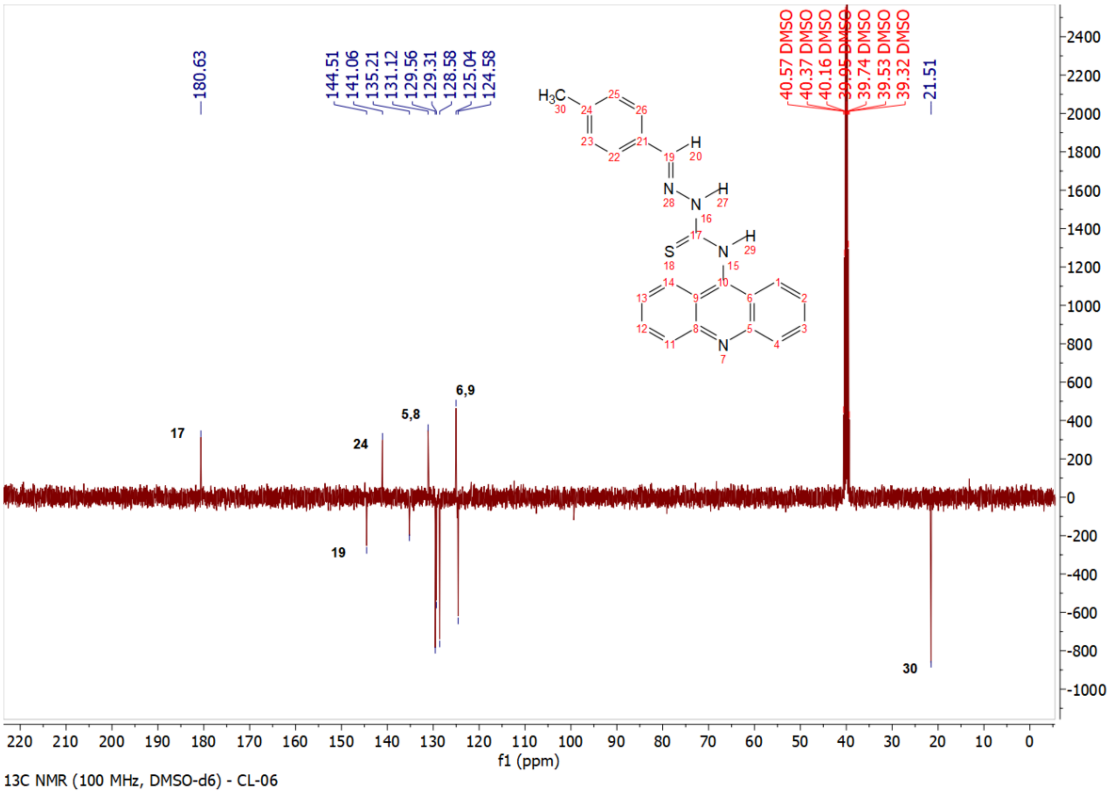

Figure S12. ^{13}C NMR spectrum of **CL-06**.

(E)-N-(acridin-9-yl)-2-(4-hydroxybenzylidene)hydrazine-1-carbothioamide (**CL-07**)

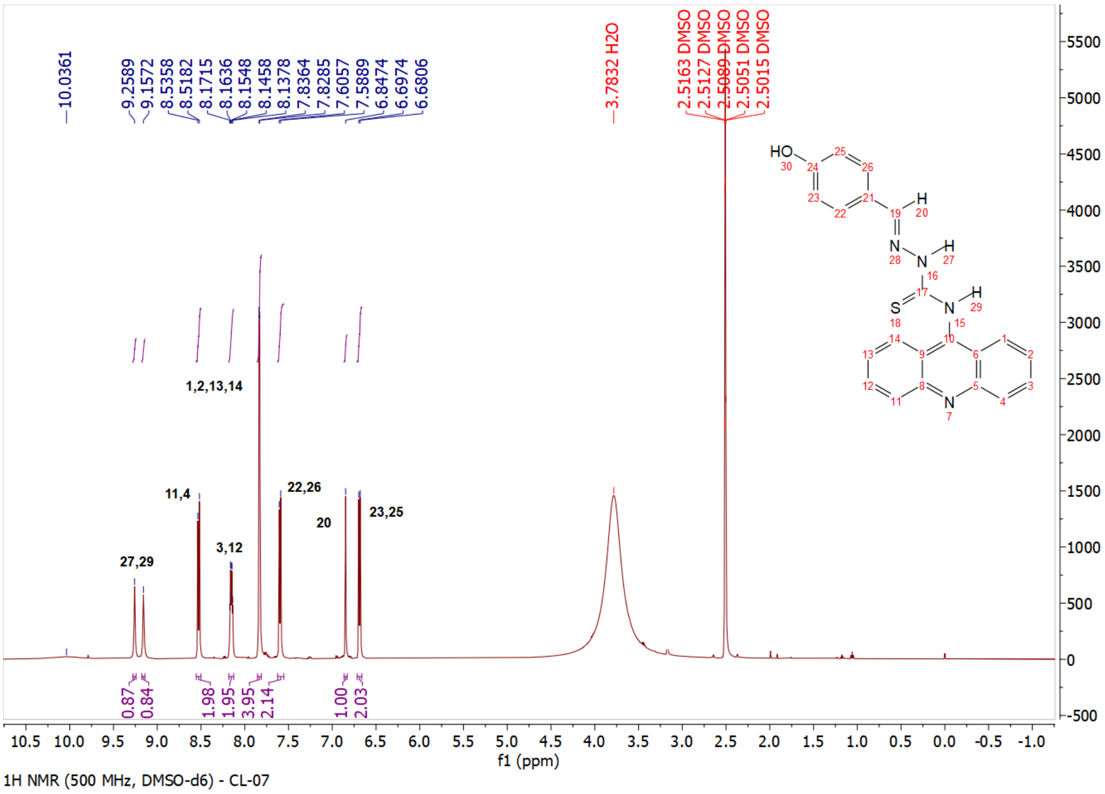

Figure S13. ^{1}H NMR spectrum of **CL-07**.

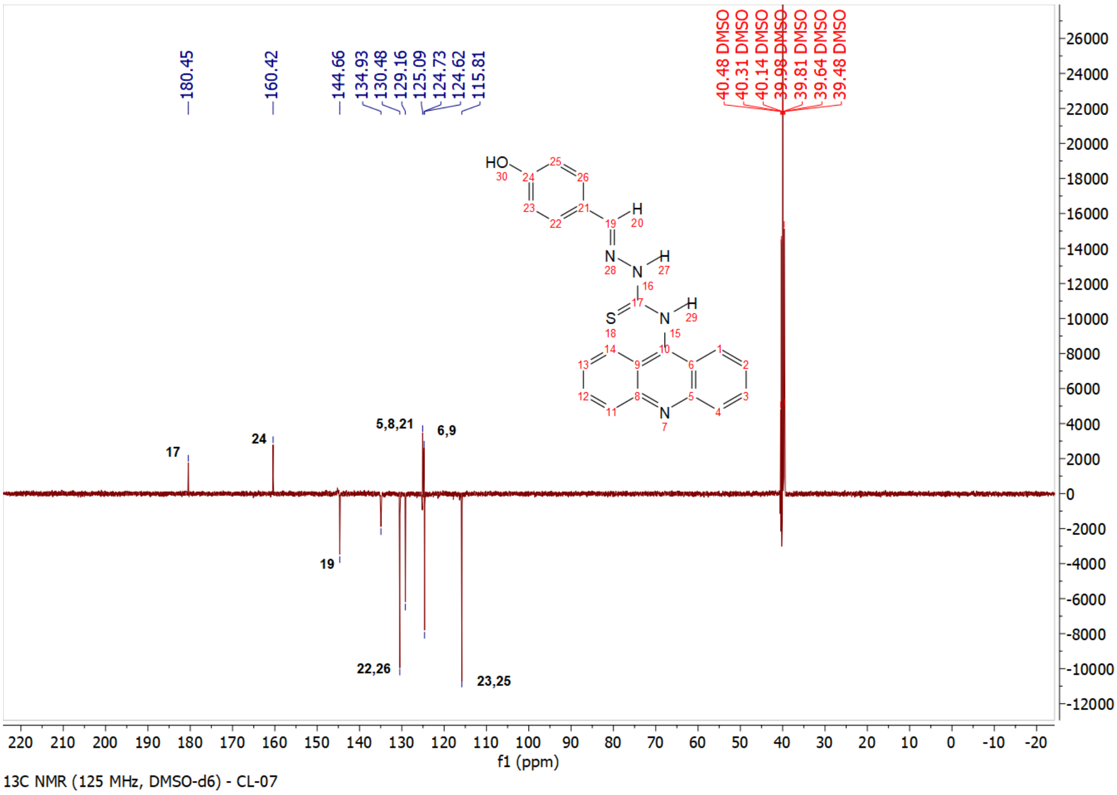

Figure S14. ^{13}C NMR spectrum of **CL-07**.

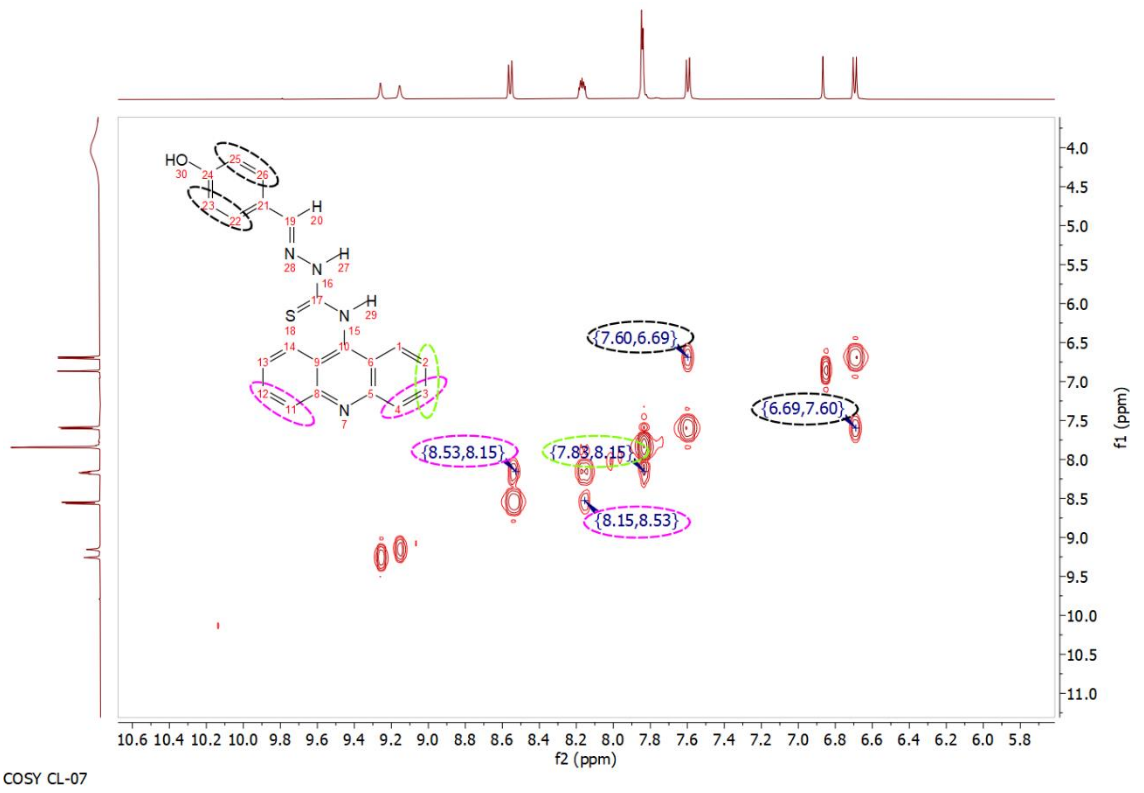

Figure S15. COSY spectrum of **CL-07 (**Solvent: DMSO).

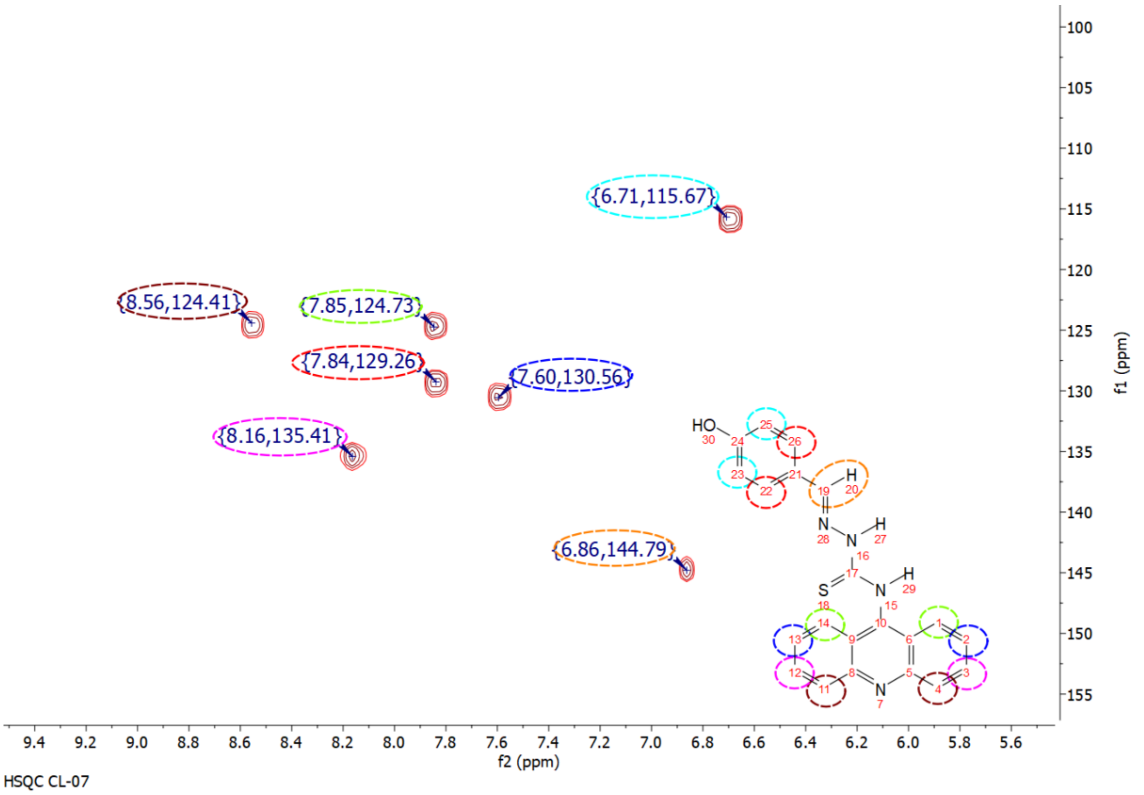

Figure S16. HSQC spectrum of **CL-07 (**Solvent: DMSO).

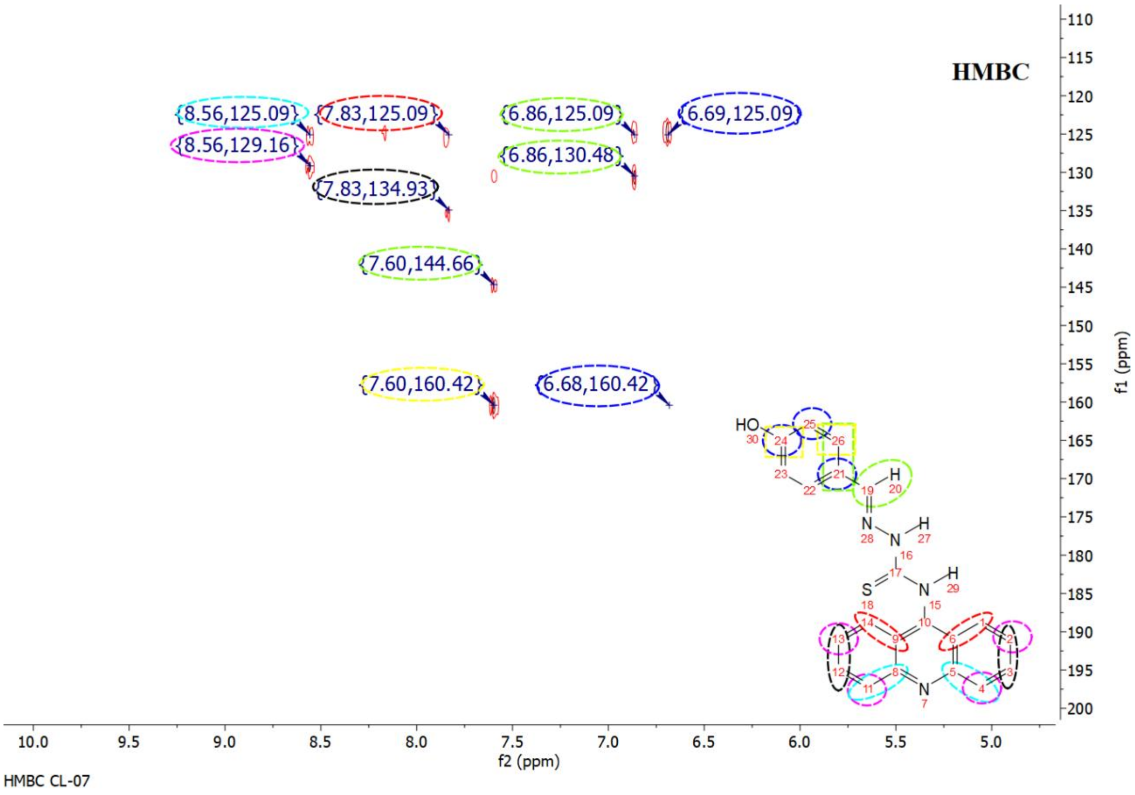

Figure S17. HMBC spectrum of **CL-07 (**Solvent: DMSO).

(E)-N-(acridin-9-yl)-2-(3-hydroxybenzylidene)hydrazine-1-carbothioamide (**CL-08**)

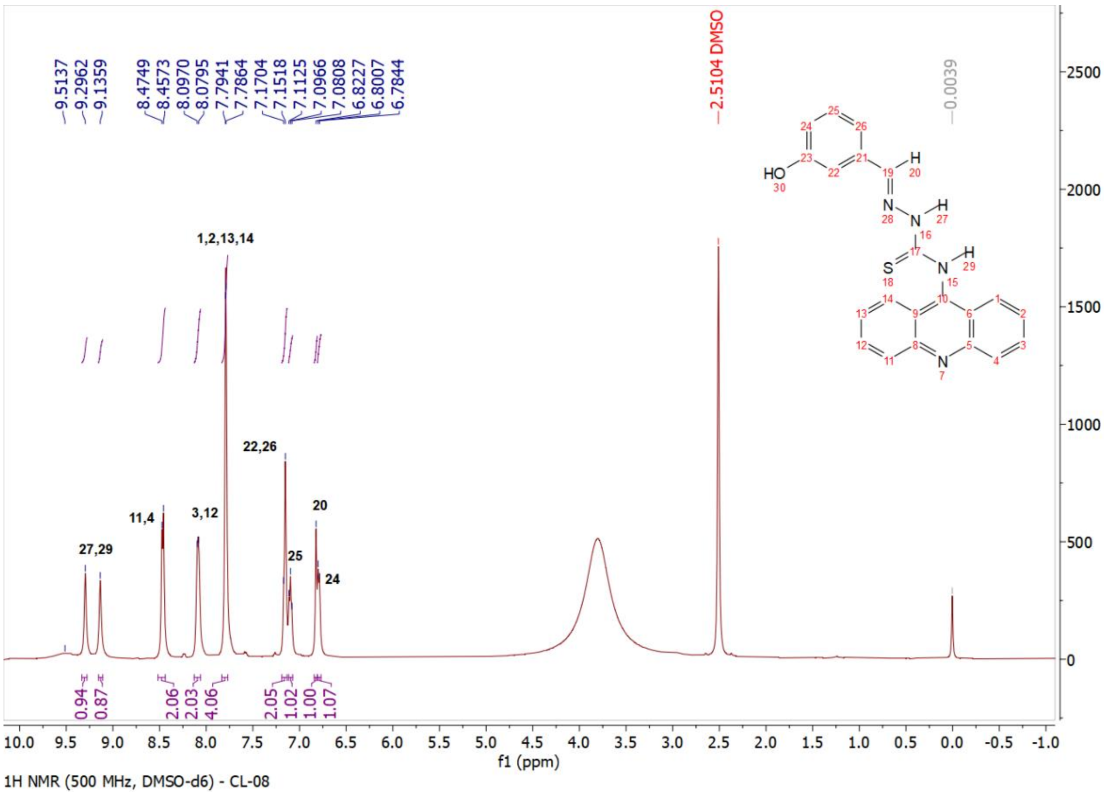

Figure S18. ^{1}H NMR spectrum of **CL-08**.

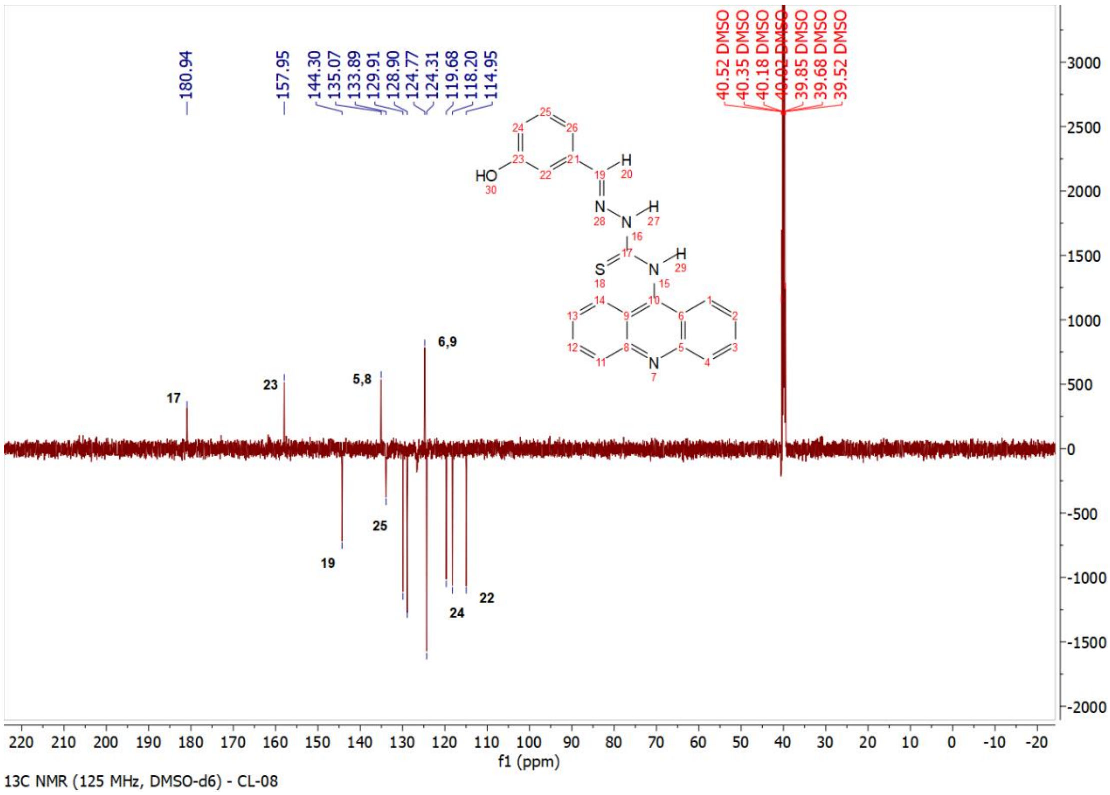

Figure S19. ^{13}C NMR spectrum of **CL-08**.

(E)-N-(acridin-9-yl)-2-(4-(dimethylamino)benzylidene)hydrazine-1-carbothioamide (**CL-09**)

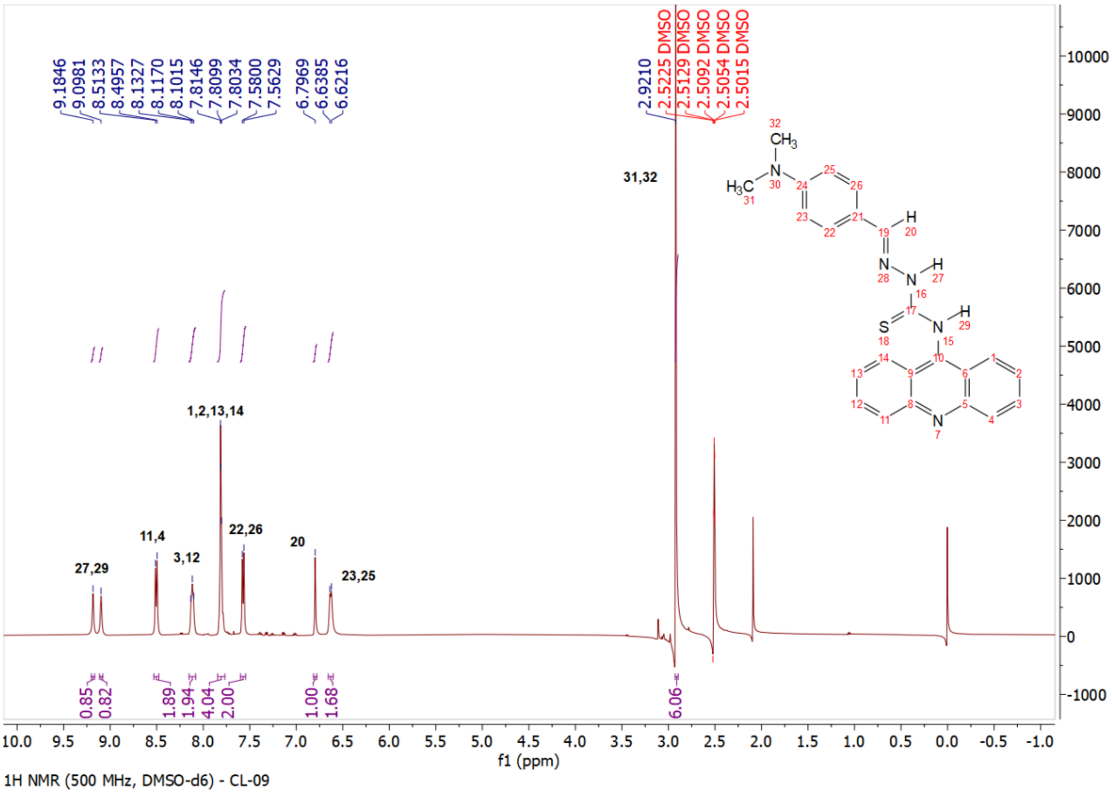

Figure S20. ^{1}H NMR spectrum of **CL-09**.

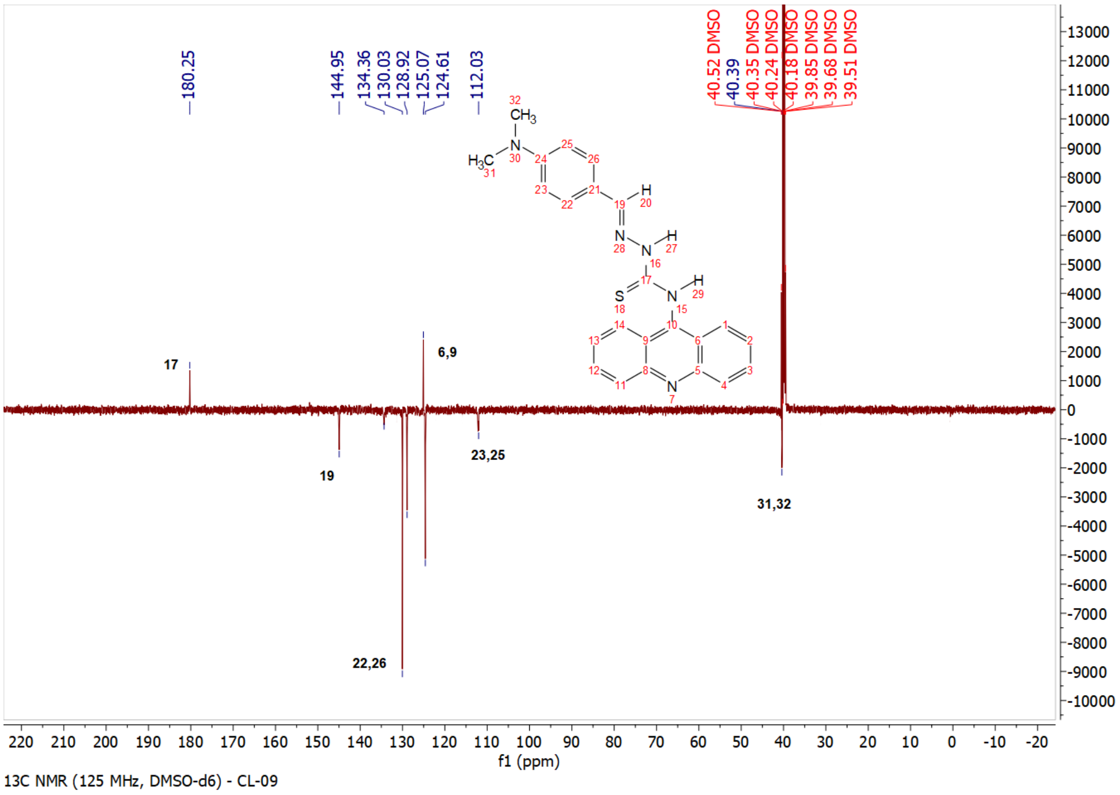

Figure S21. ^{13}C NMR spectrum of **CL-09**.

(E)-N-(acridin-9-yl)-2-(4-bromobenzylidene)hydrazine-1-carbothioamide (**CL-10**)

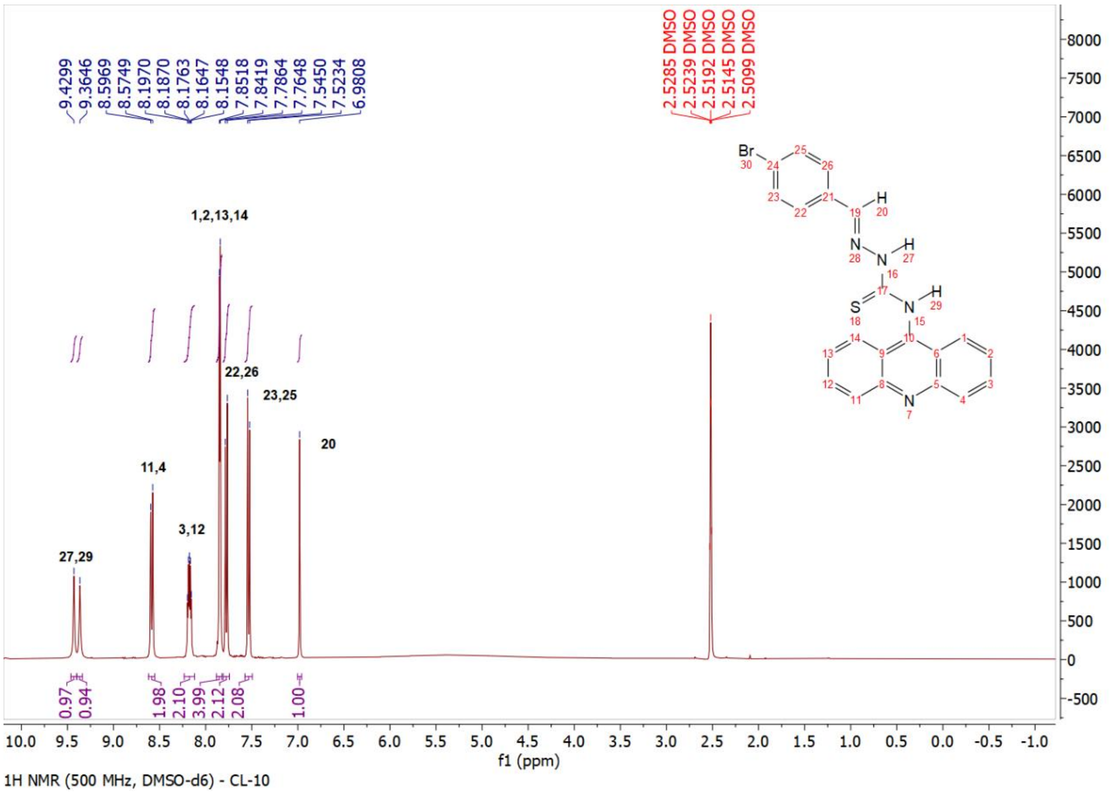

Figure S22. ^{1}H NMR spectrum of **CL-10**.

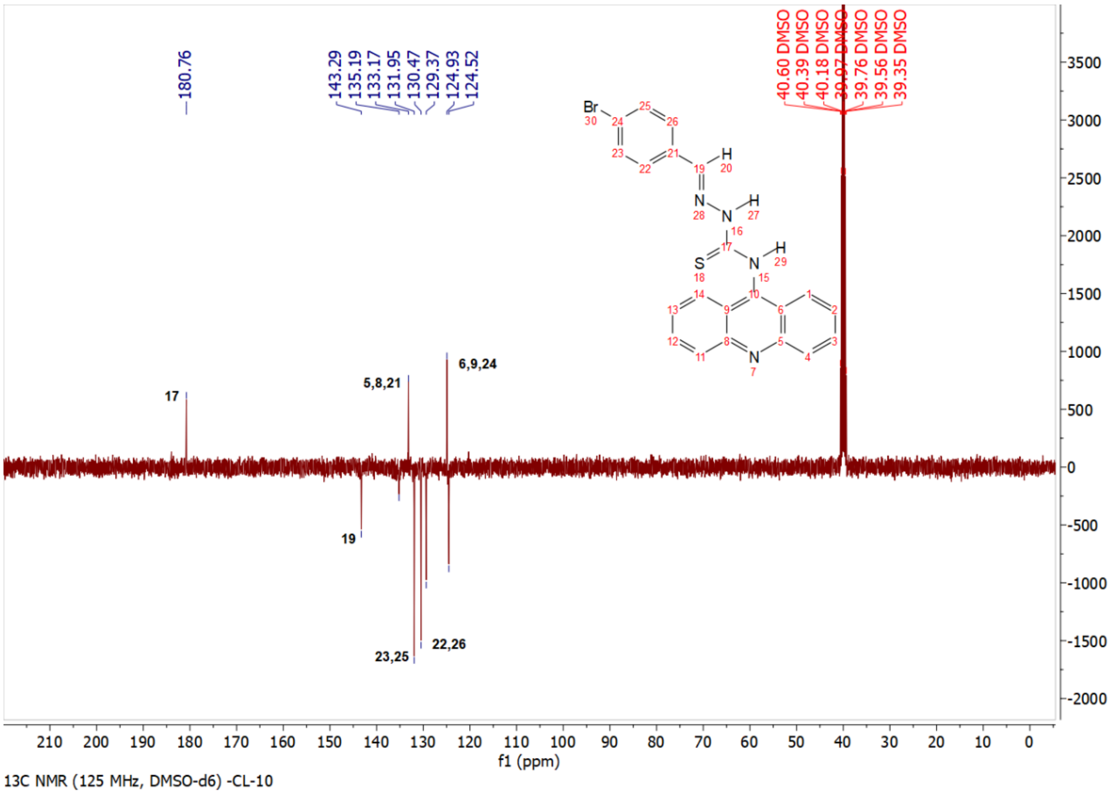

Figure S23. ^{13}C NMR spectrum of **CL-10**.

(E)-2-benzylidene-N-(6-chloro-2-methoxyacridin-9-yl)hydrazine-1-carbothioamide (**DL-01**)

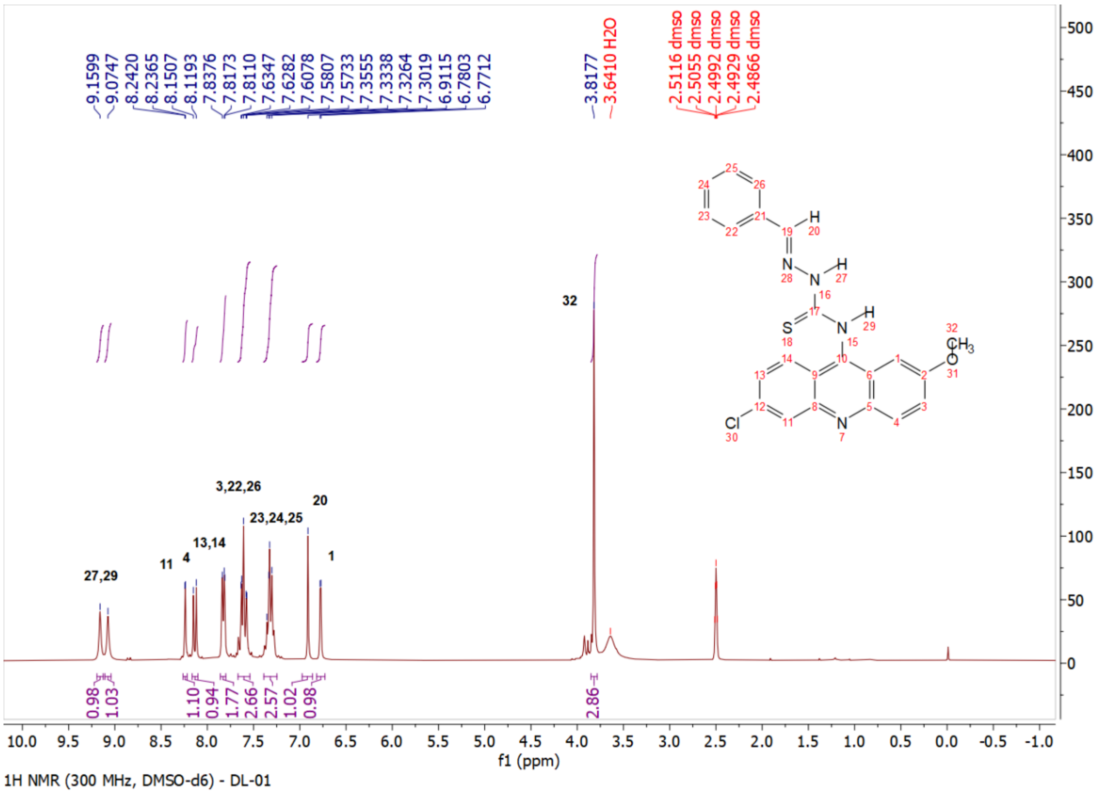

Figure S24. ^{1}H NMR spectrum of **DL-01**.

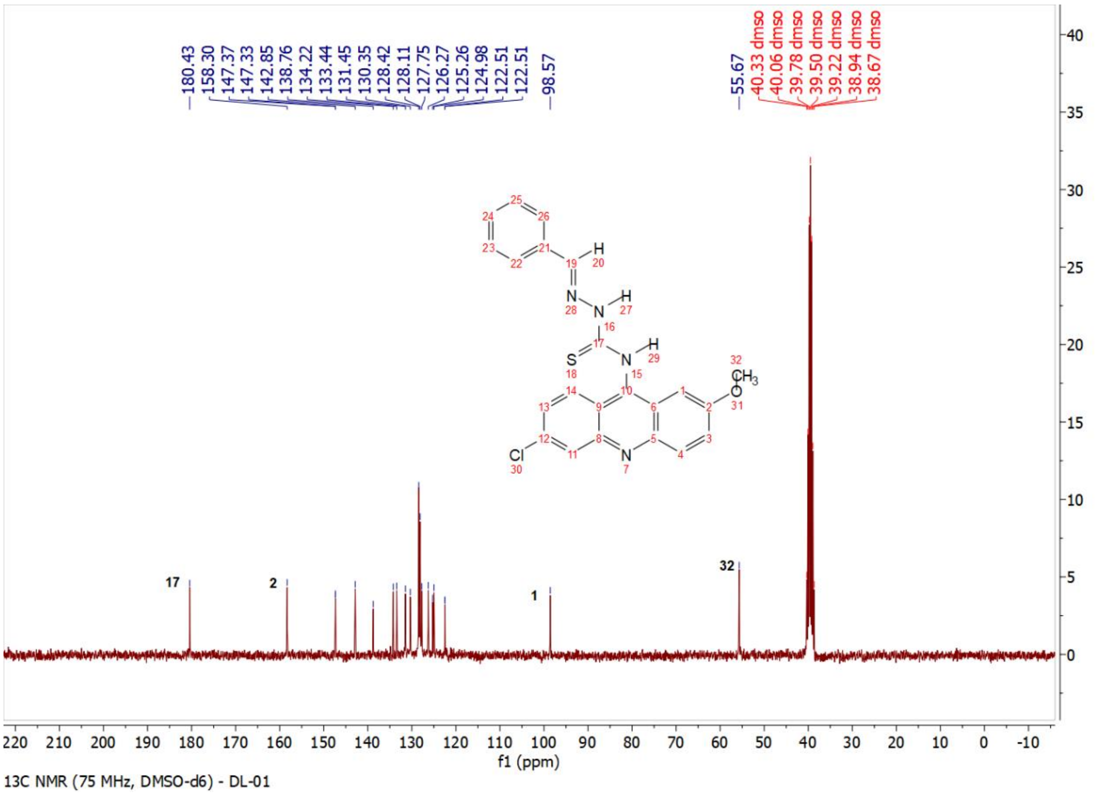

Figure S25. ^{13}C NMR spectrum of **DL-01**.

(E)-N-(6-chloro-2-methoxyacridin-9-yl)-2-(4-chlorobenzylidene)hydrazine-1-carbothioamide (**DL-02**)

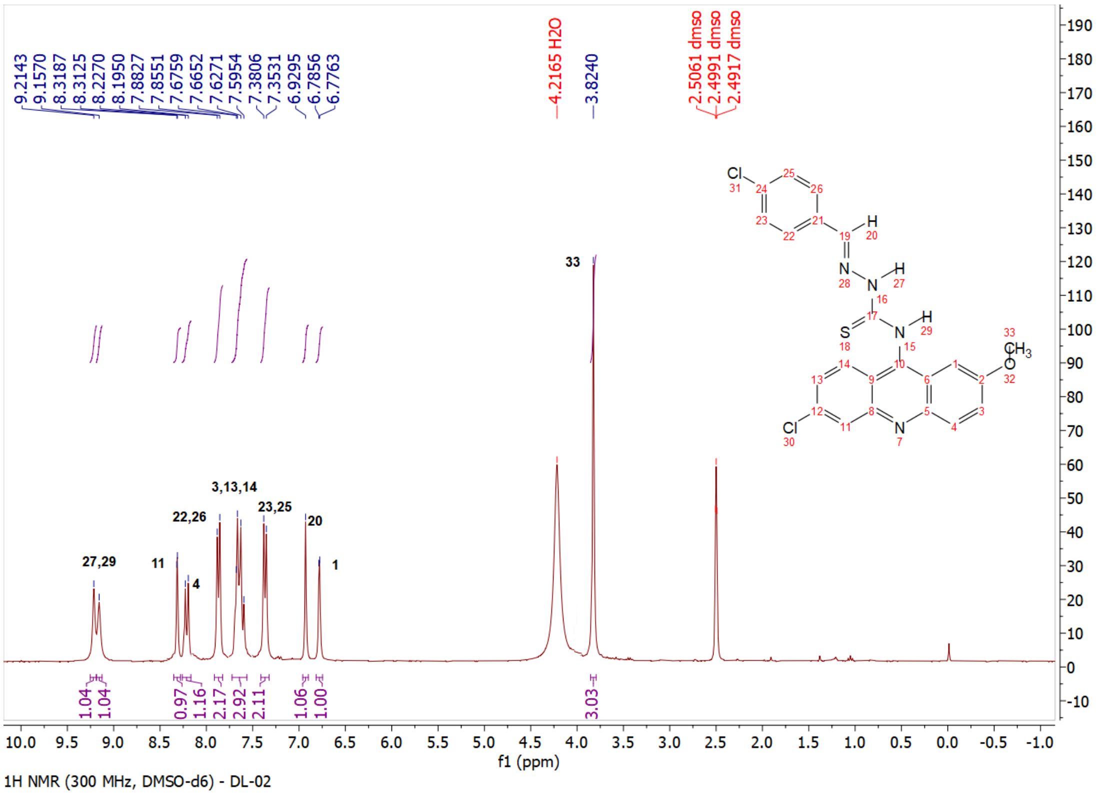

Figure S26. ^{1}H NMR spectrum of **DL-02**.

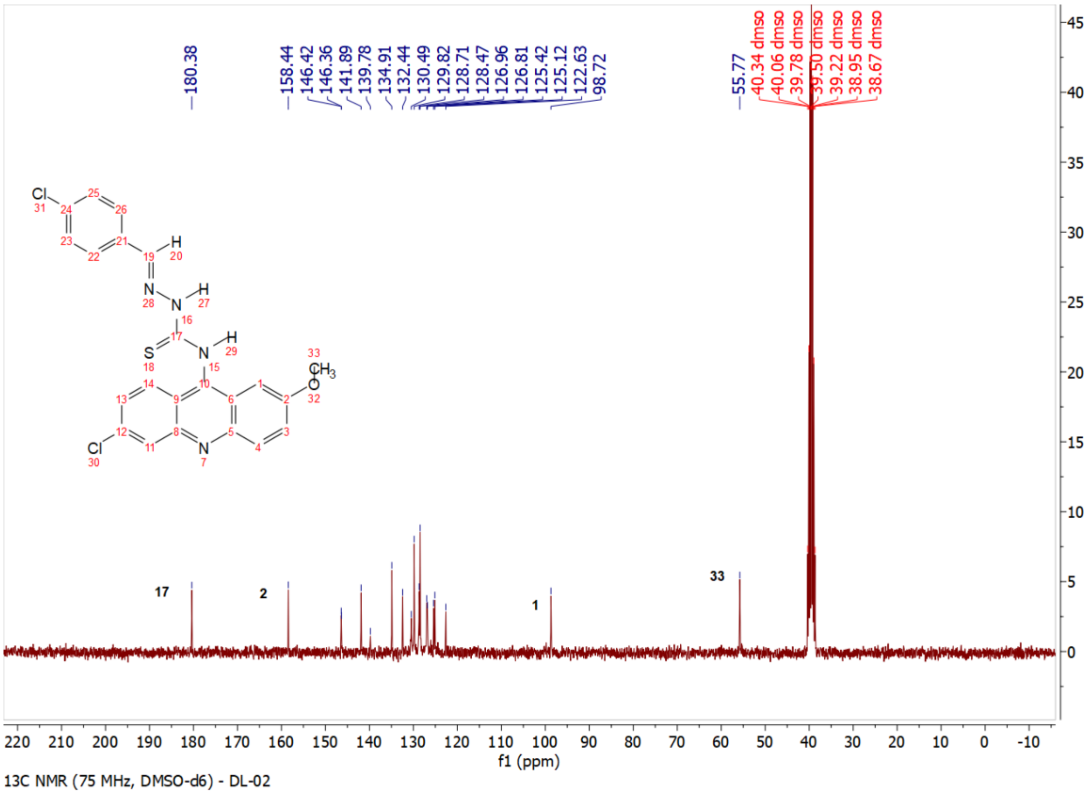

Figure S27. ^{13}C NMR spectrum of **DL-02**.

(E)-N-(6-chloro-2-methoxyacridin-9-yl)-2-(4-methoxybenzylidene)hydrazine-1-carbothioamide (**DL-03**)

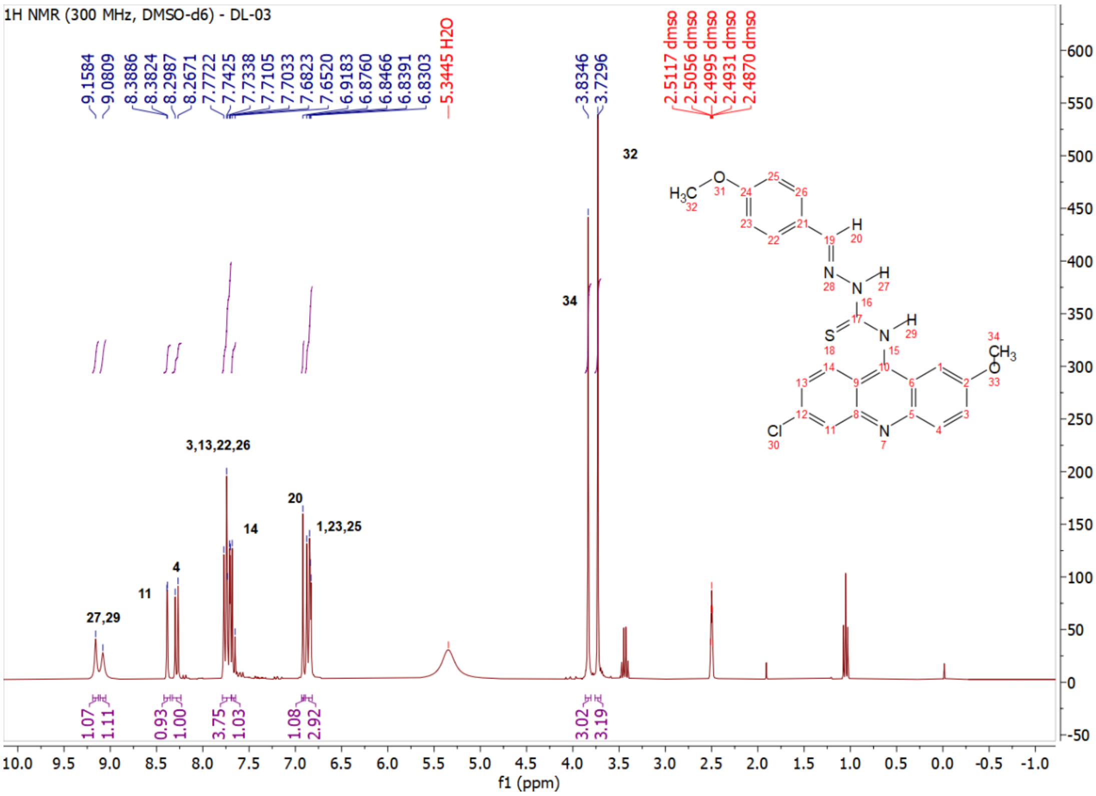

Figure S28. ^{1}H NMR spectrum of **DL-03**.

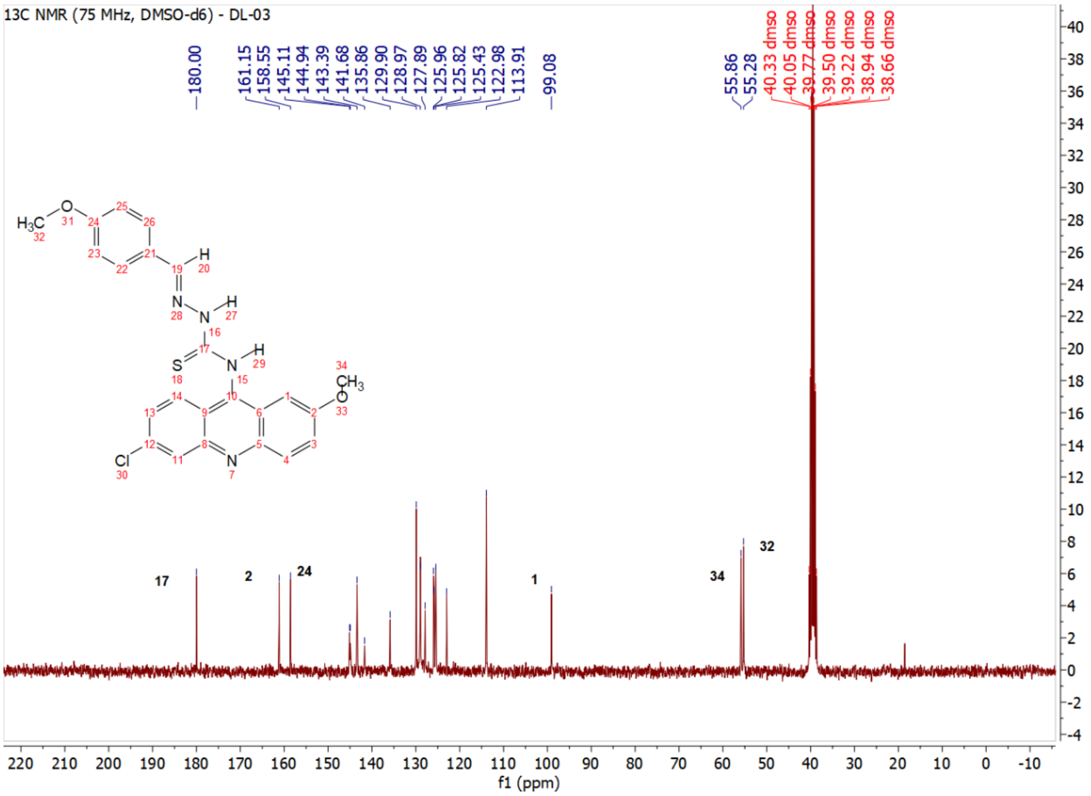

Figure S29. ^{13}C NMR spectrum of **DL-03**.

(E)-N-(6-chloro-2-methoxyacridin-9-yl)-2-(2,4-dichlorobenzylidene)hydrazine-1-carbothioamide (**DL-04**)

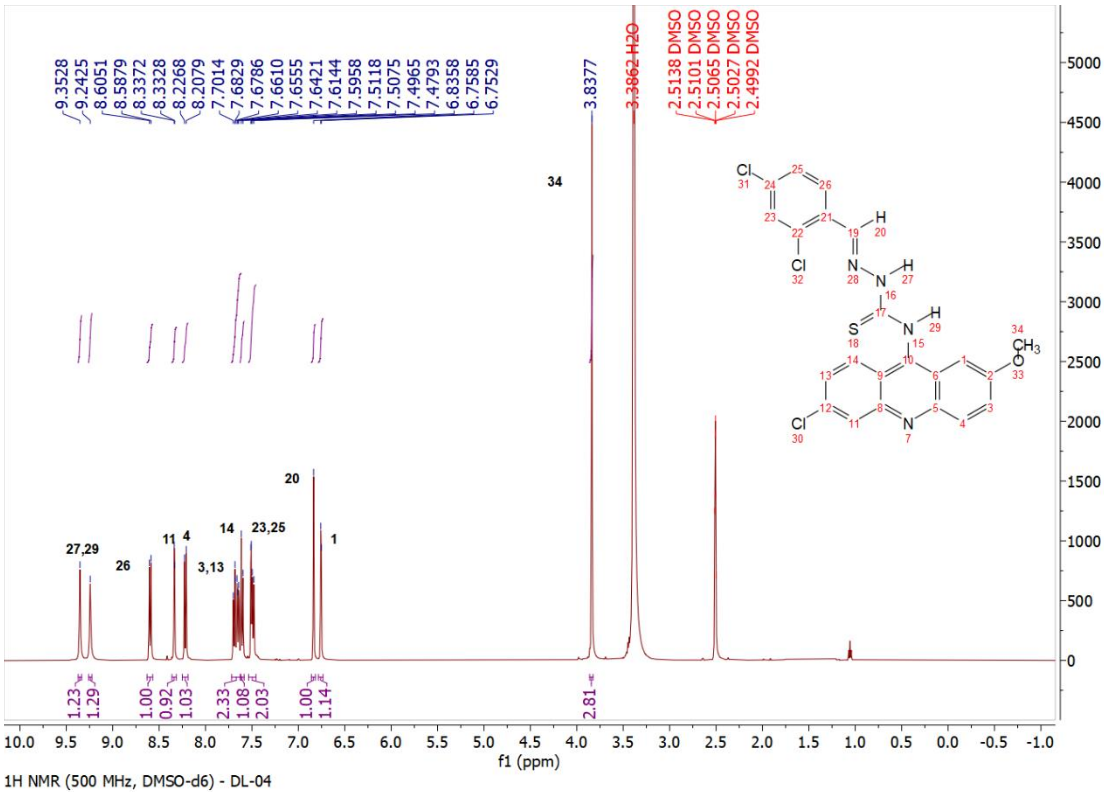

Figure S30. ^{1}H NMR spectrum of **DL-04**.

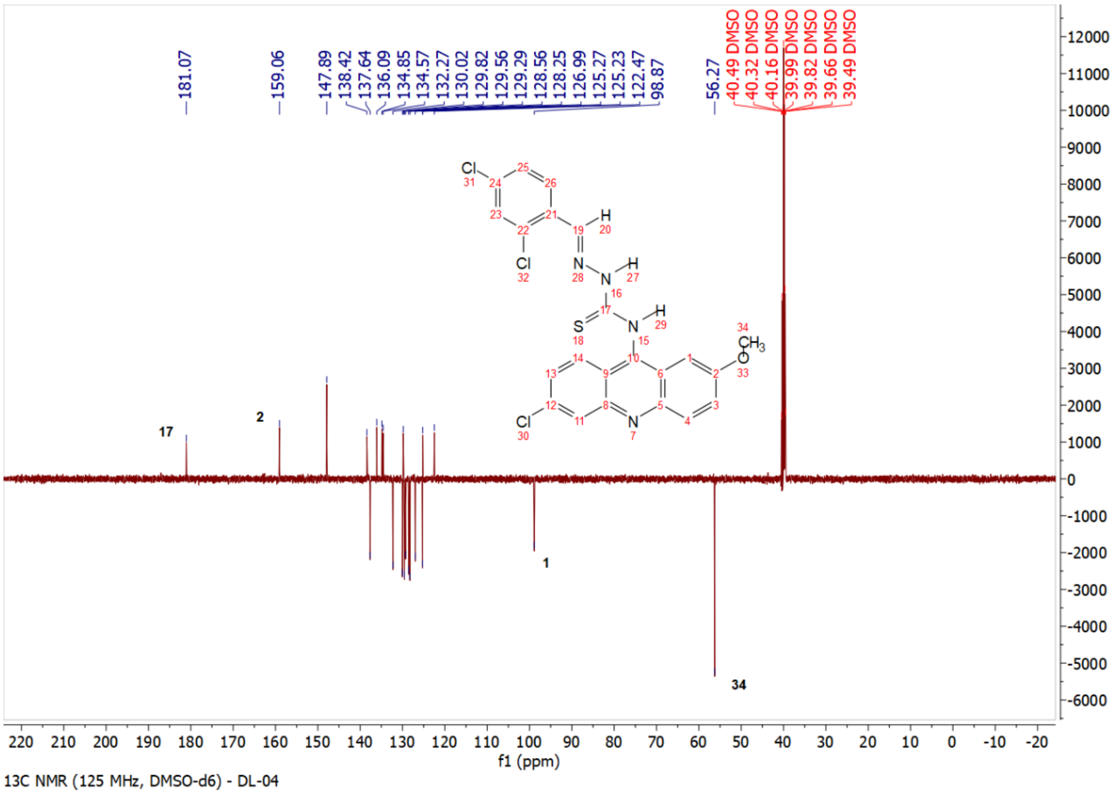

Figure S31. ^{13}C NMR spectrum of **DL-04**.

(E)-N-(6-chloro-2-methoxyacridin-9-yl)-2-(4-nitrobenzylidene)hydrazine-1-carbothioamide (**DL-05**)

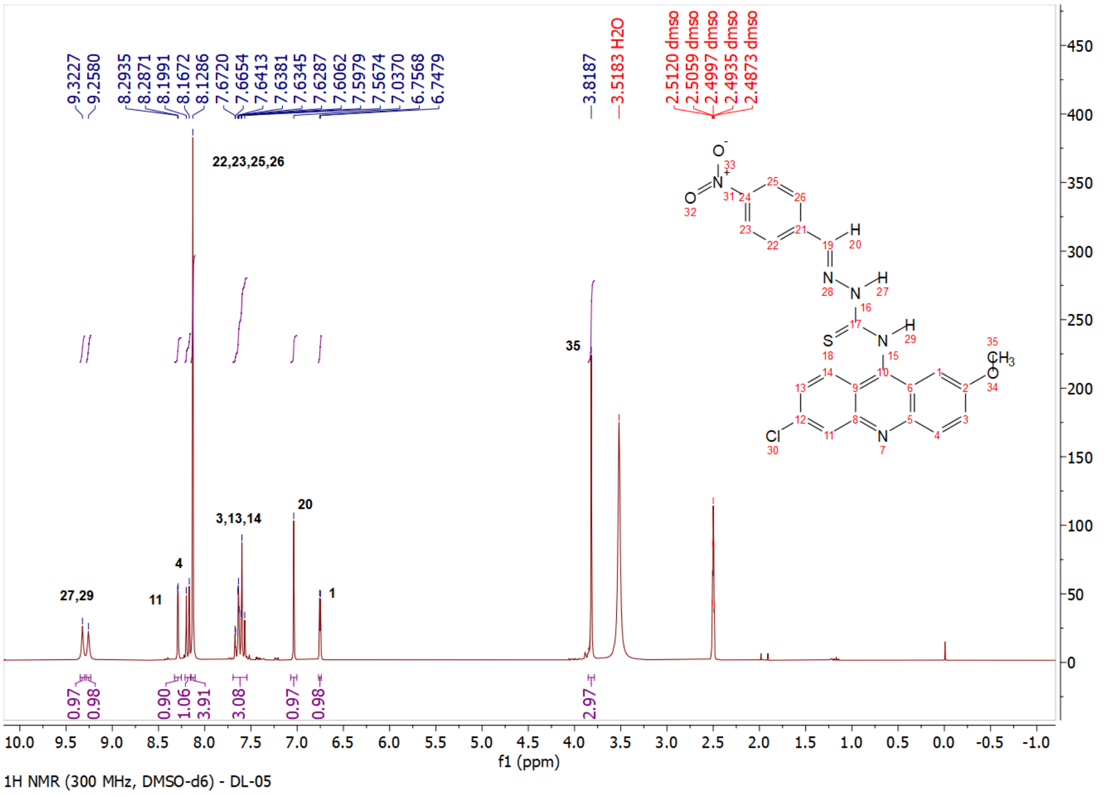

Figure S32. ^{1}H NMR spectrum of **DL-05**.

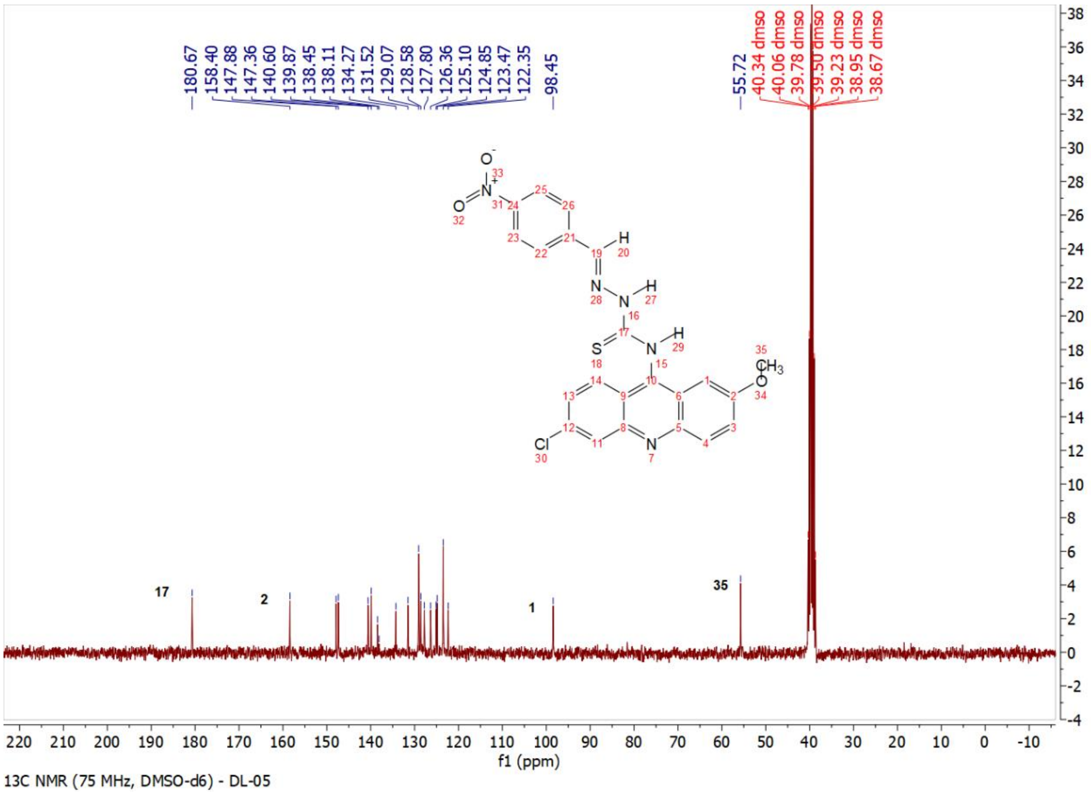

Figure S33. ^{13}C NMR spectrum of **DL-05**.

(E)-N-(6-chloro-2-methoxyacridin-9-yl)-2-(4-methylbenzylidene)hydrazine-1-carbothioamide (**DL-06**)

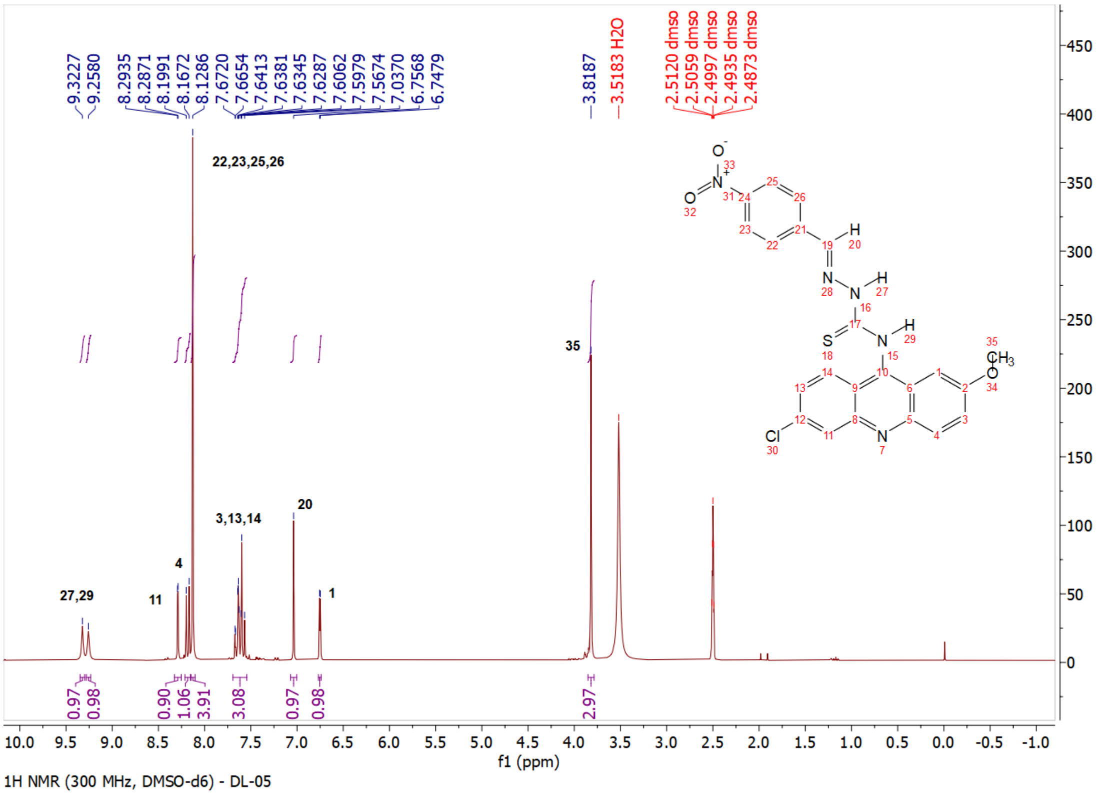

Figure S34. ^{1}H NMR spectrum of **DL-06**.

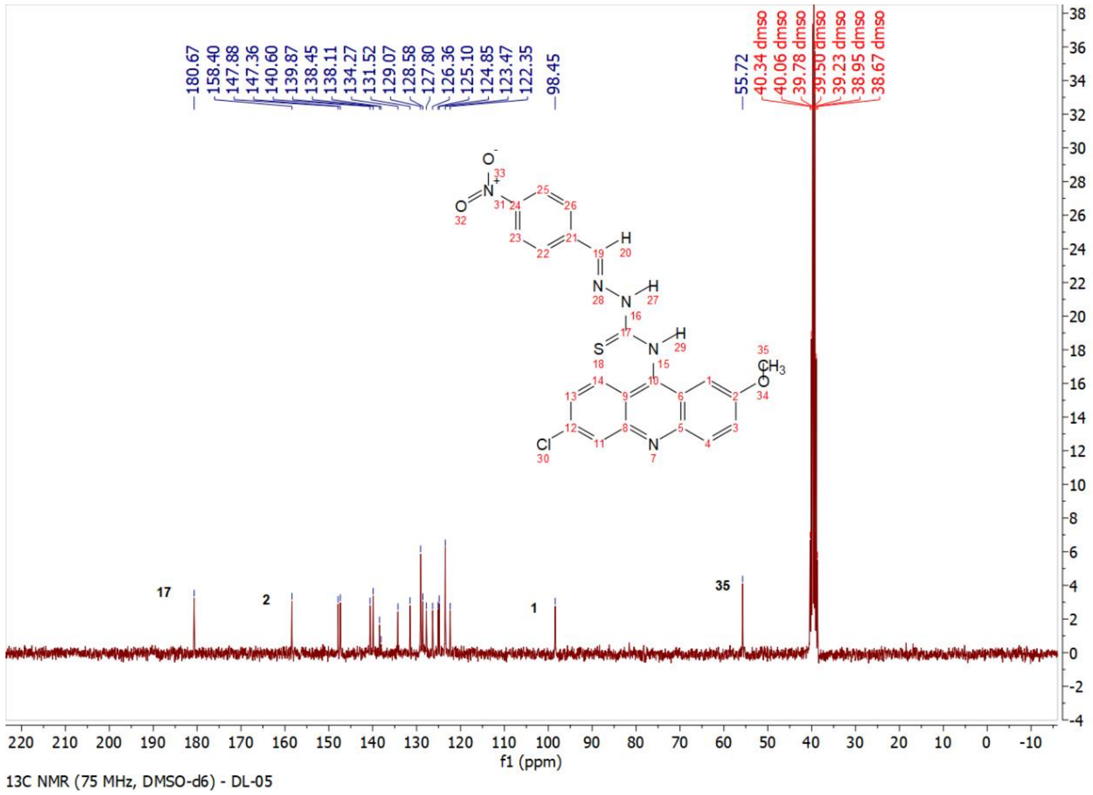

Figure S35. ^{13}C NMR spectrum of **DL-06**.

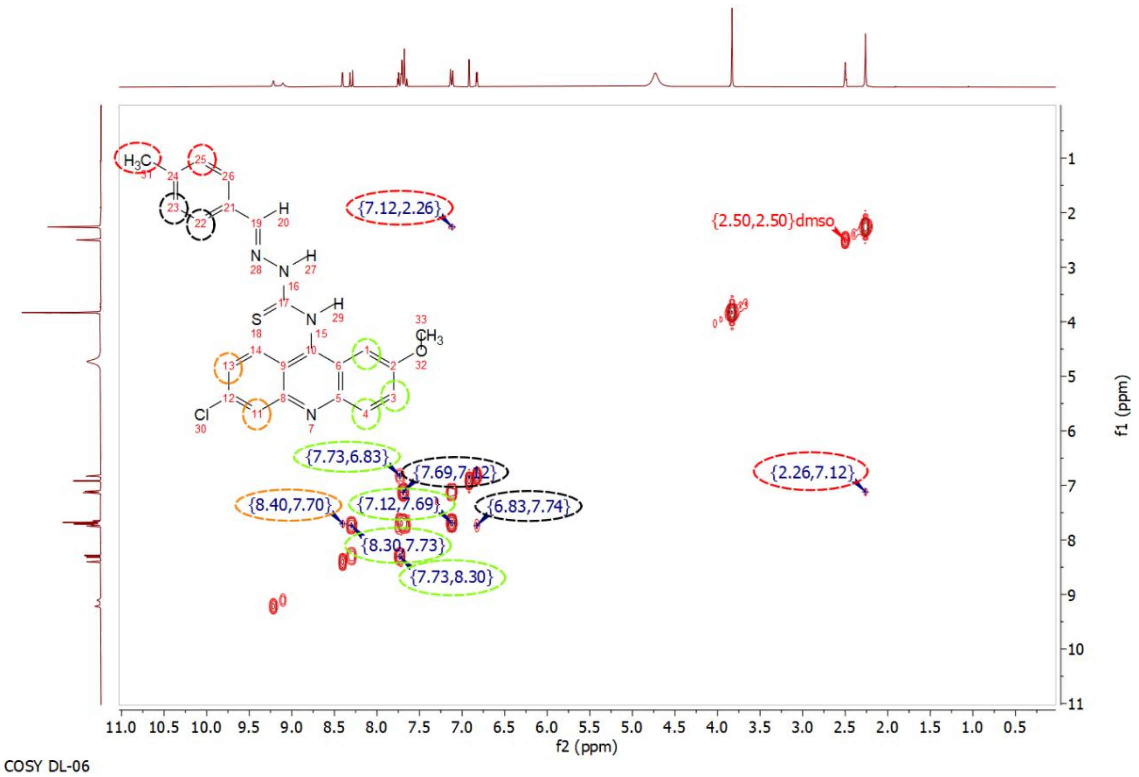

Figure S36. COSY spectrum of **DL-06 (**Solvent: DMSO).

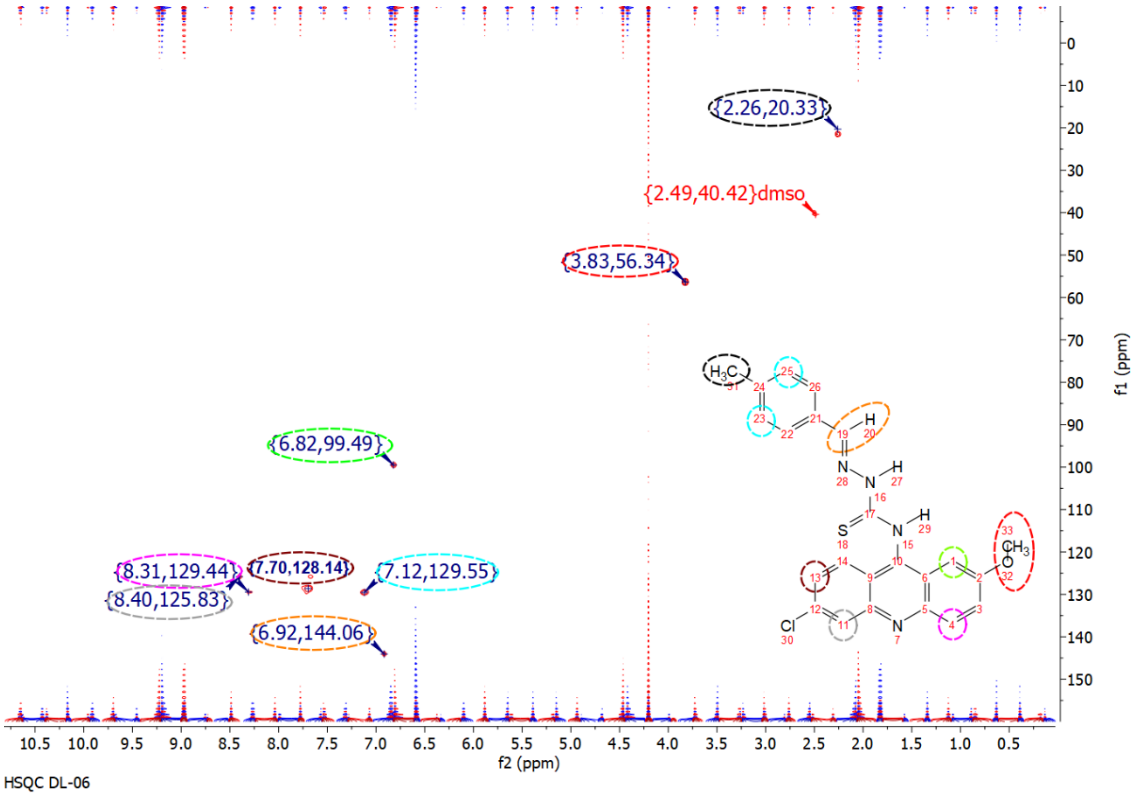

Figure S37. HSQC spectrum of **DL-06 (**Solvent: DMSO).

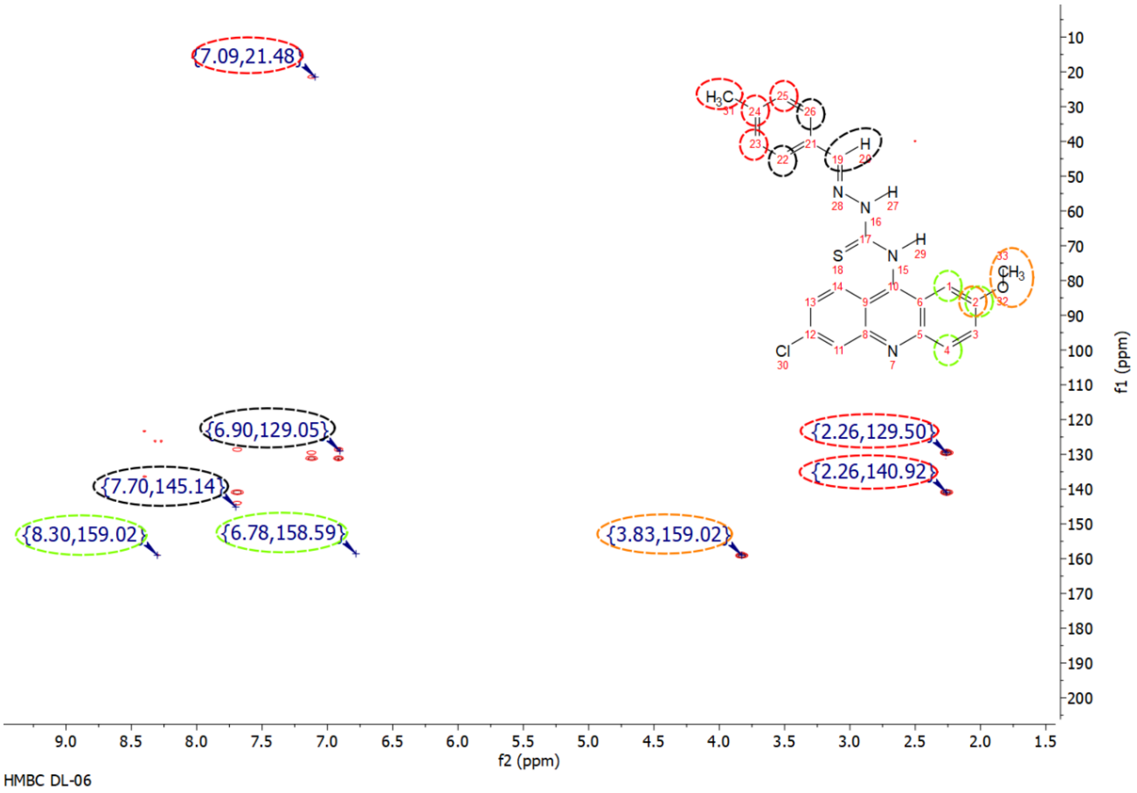

Figure S38. HMBC spectrum of **DL-06 (**Solvent: DMSO).

(E)-N-(6-chloro-2-methoxyacridin-9-yl)-2-(4-hydroxybenzylidene)hydrazine-1-carbothioamide (**DL-07**)

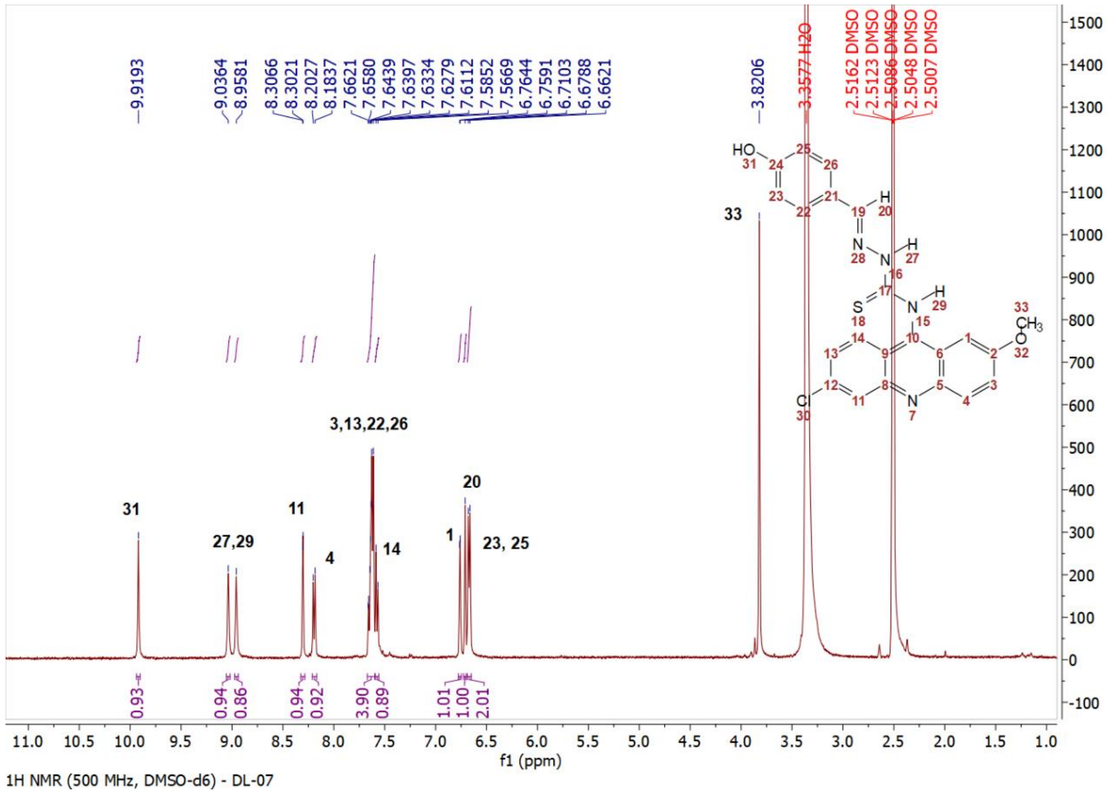

Figure S39. ^{1}H NMR spectrum of **DL-07**.

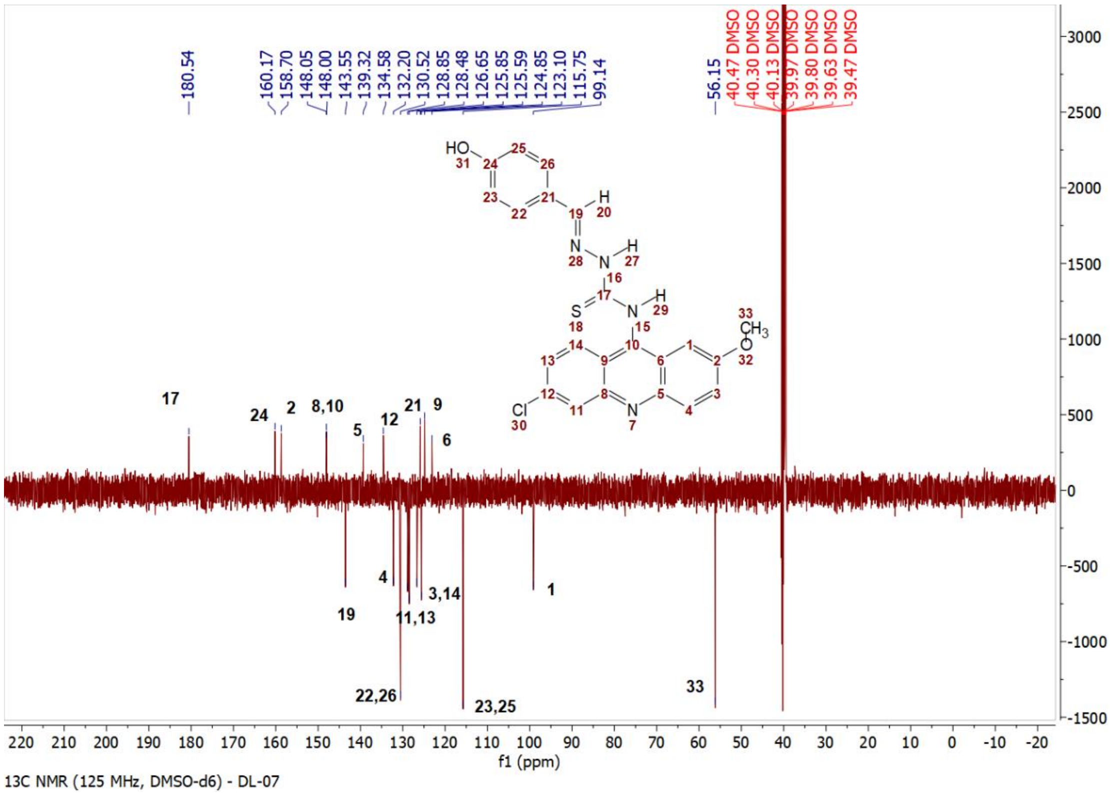

Figure S40. ^{13}C NMR spectrum of **DL-07**.

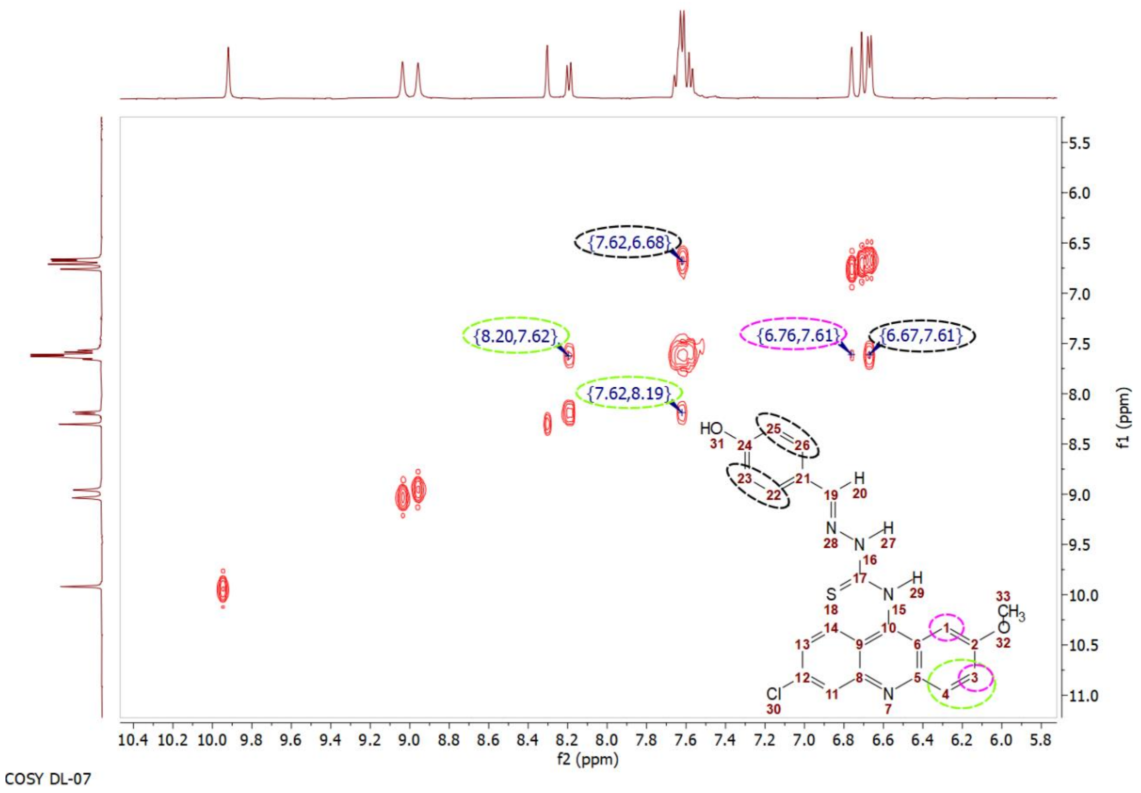

Figure S41. COSY spectrum of **DL-07 (**Solvent: DMSO).

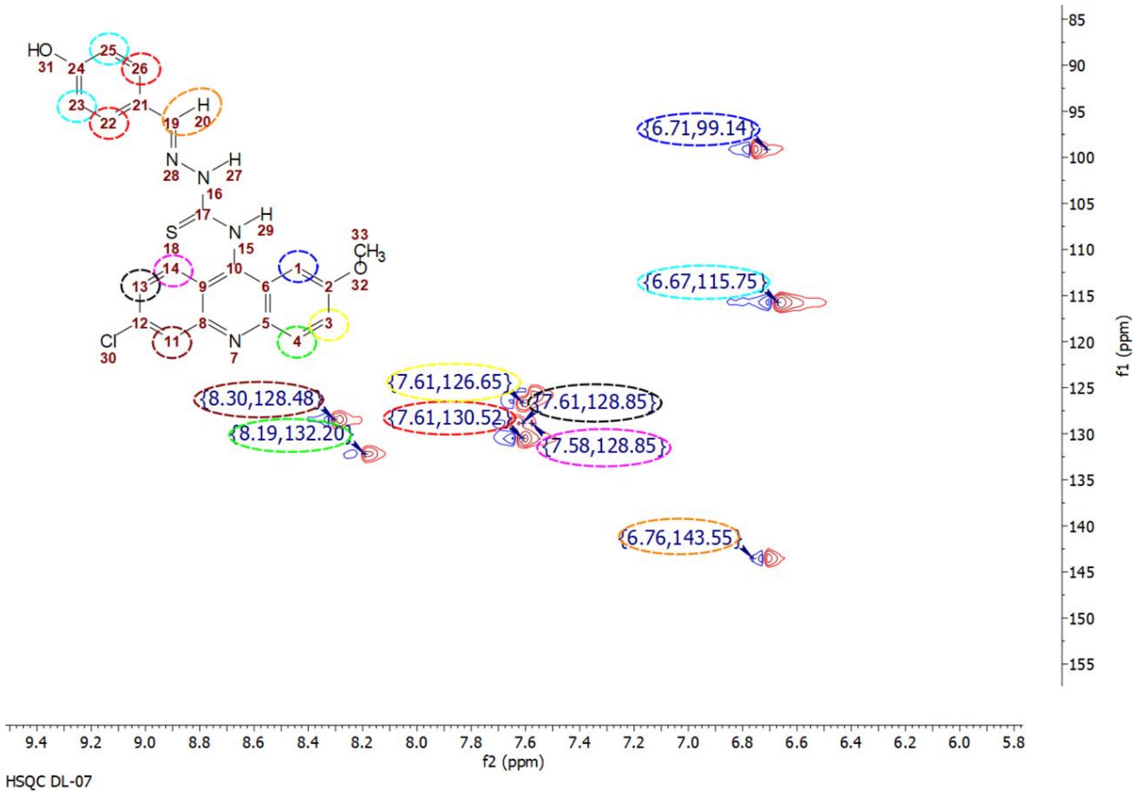

Figure S42. HSQC spectrum of **DL-07 (**Solvent: DMSO).

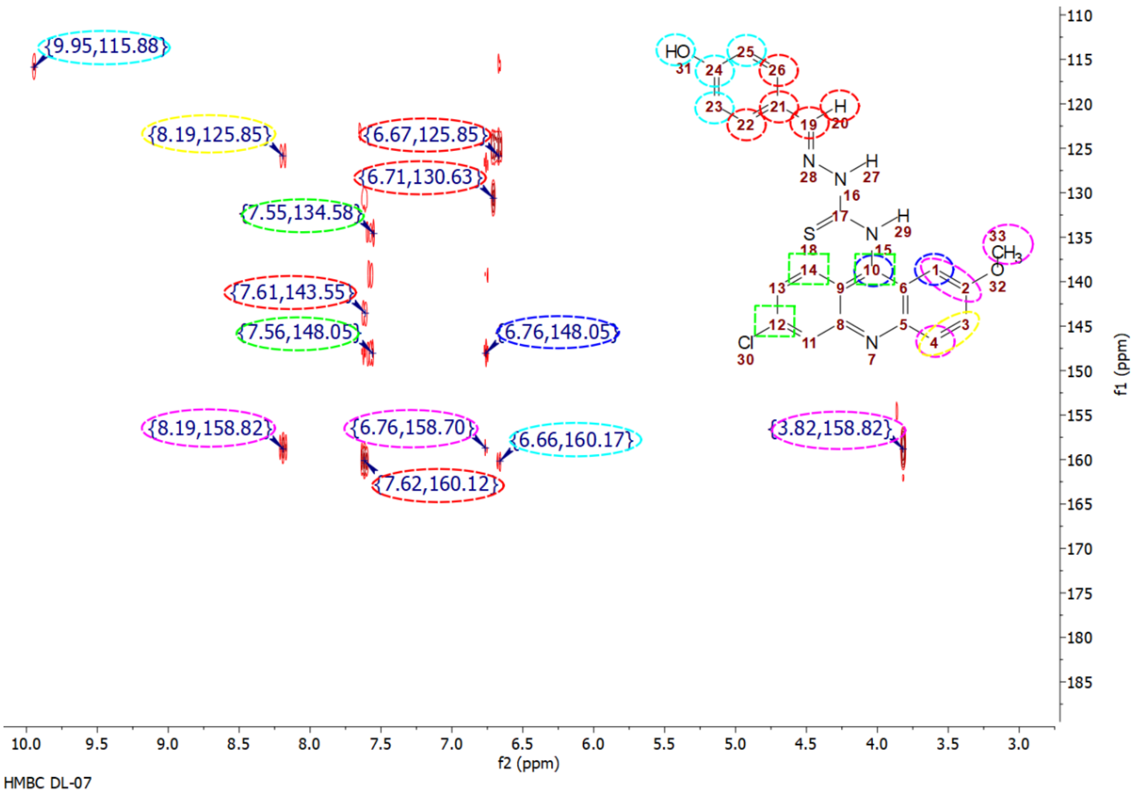

Figure S43. HMBC spectrum of **DL-07** (Solvent: DMSO).

(E)-N-(6-chloro-2-methoxyacridin-9-yl)-2-(3-hydroxybenzylidene)hydrazine-1-carbothioamide (**DL-08**)

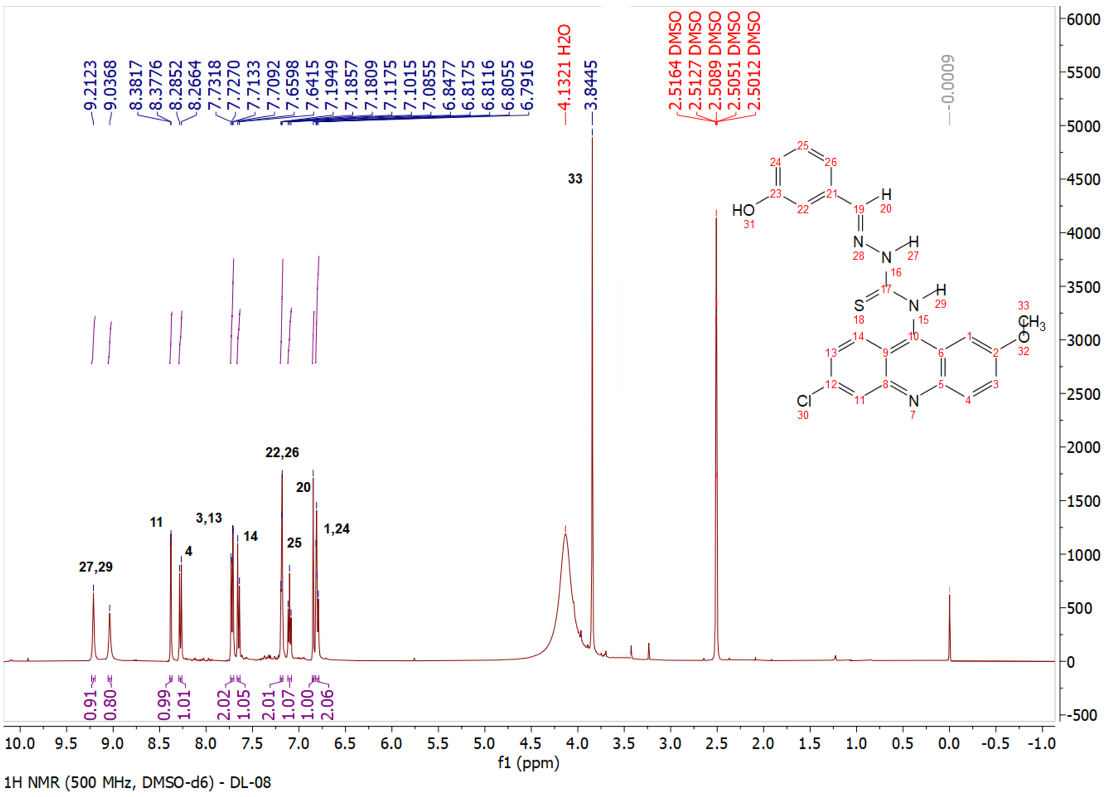

Figure S44. ^{1}H NMR spectrum of **DL-08**.

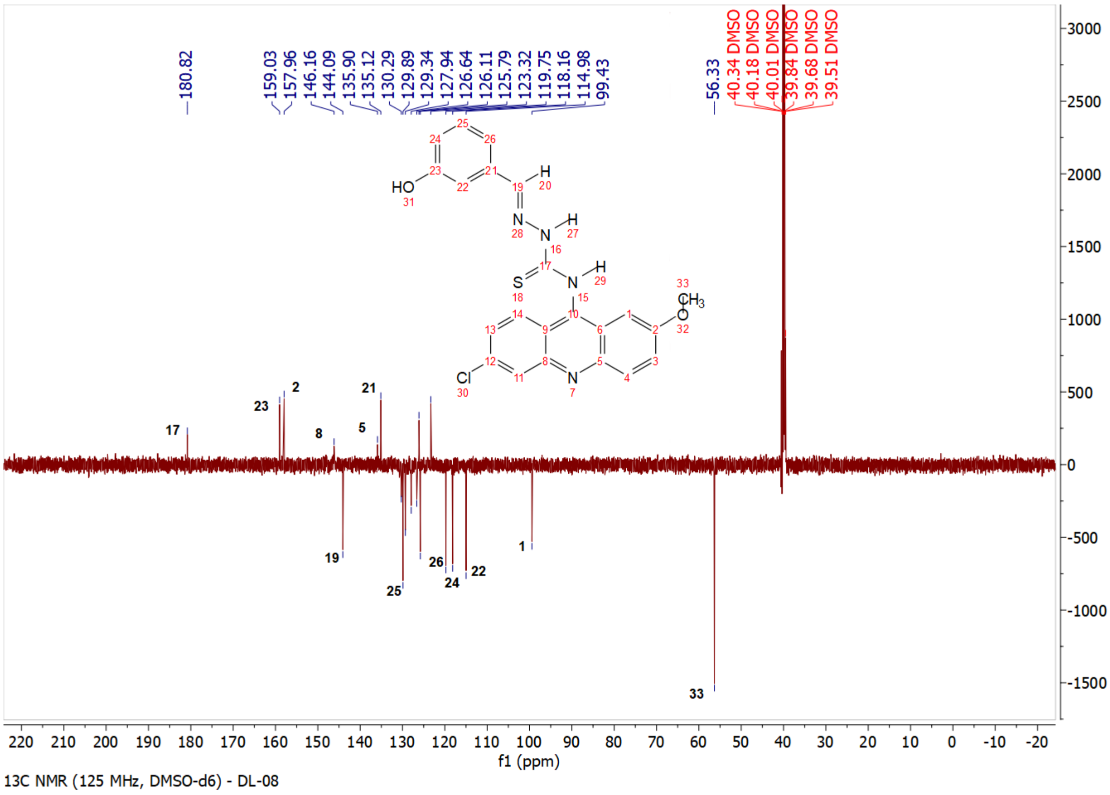

Figure S45. ^{13}C NMR spectrum of **DL-08**.

(E)-N-(6-chloro-2-methoxyacridin-9-yl)-2-(4-(dimethylamino)benzylidene)hydrazine-1-carbothioamide (**DL-09**)

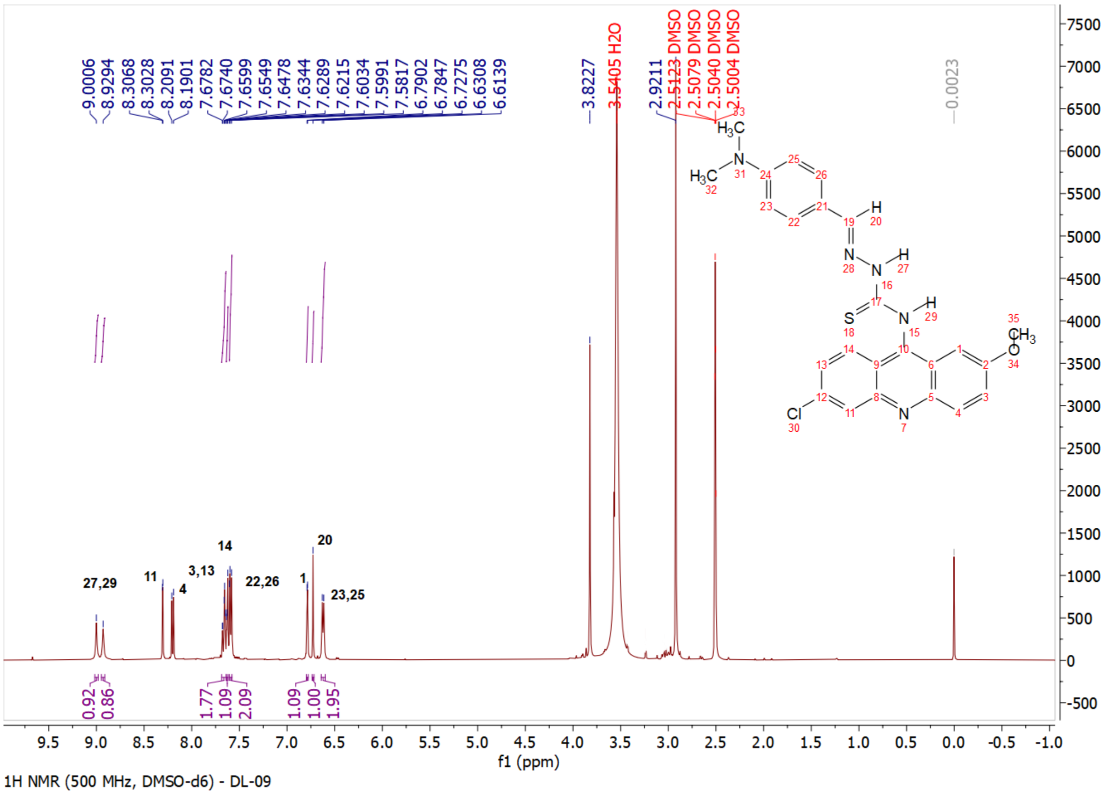

Figure S46. ^{1}H NMR spectrum of **DL-09**.

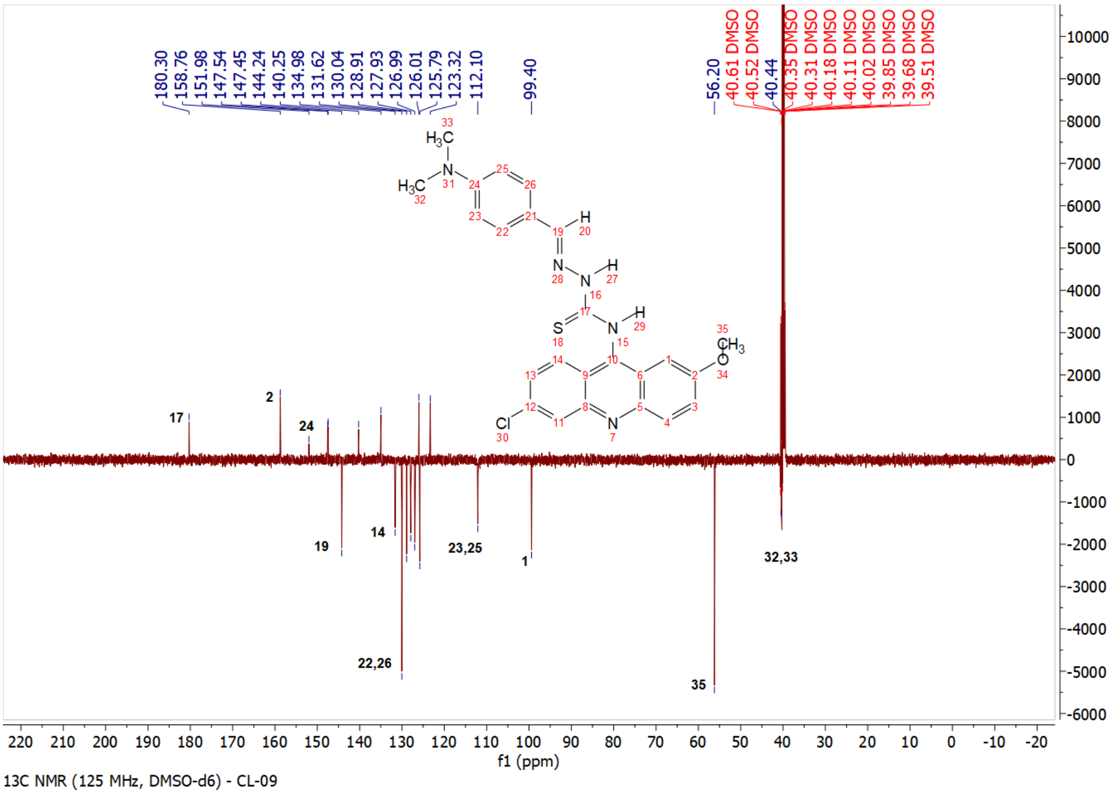

Figure S47. ^{13}C NMR spectrum of **DL-09**.

(E)-2-(4-bromobenzylidene)-N-(6-chloro-2-methoxyacridin-9-yl)hydrazine-1-carbothioamide (**DL-10**)

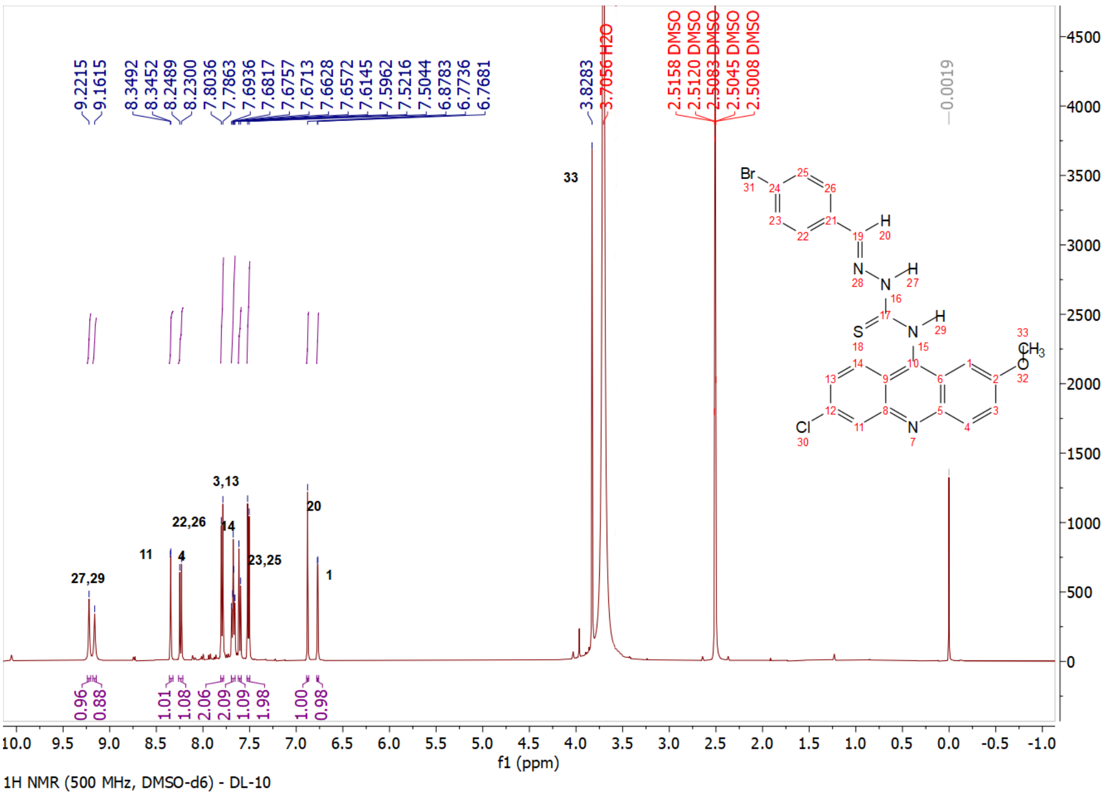

Figure S48. ^{1}H NMR spectrum of **DL-10**.

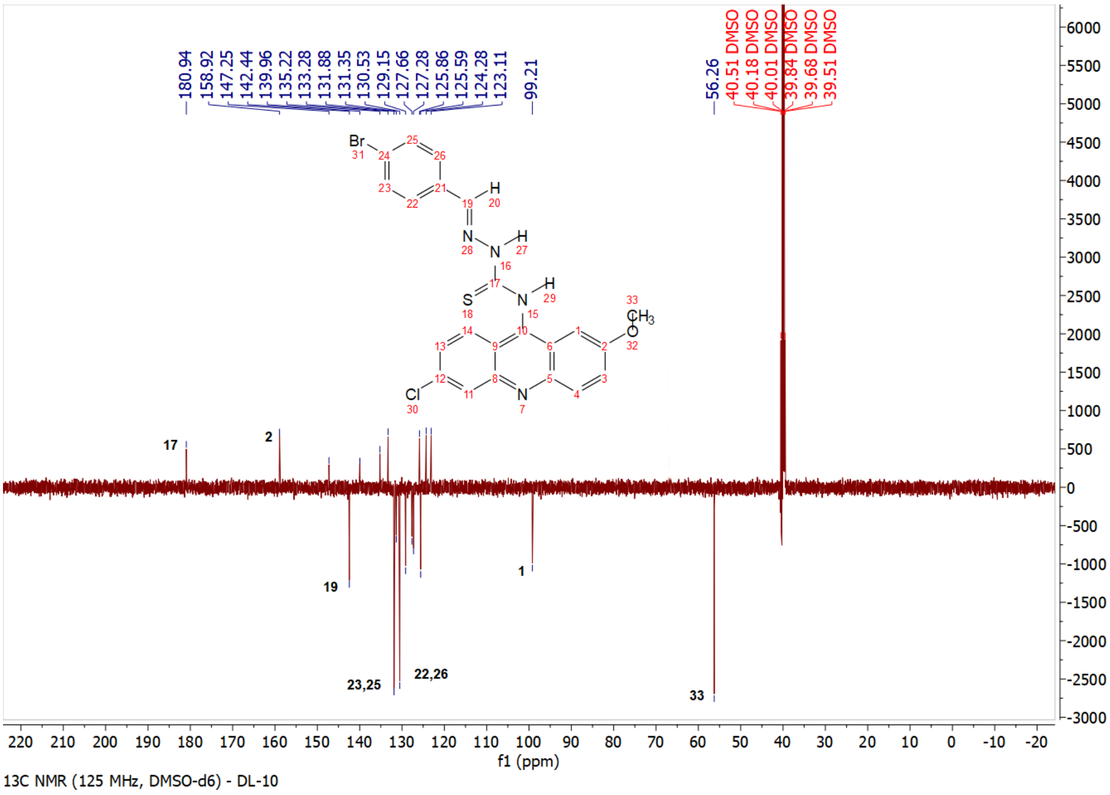

Figure S49. ^{13}C NMR spectrum of **DL-10**.

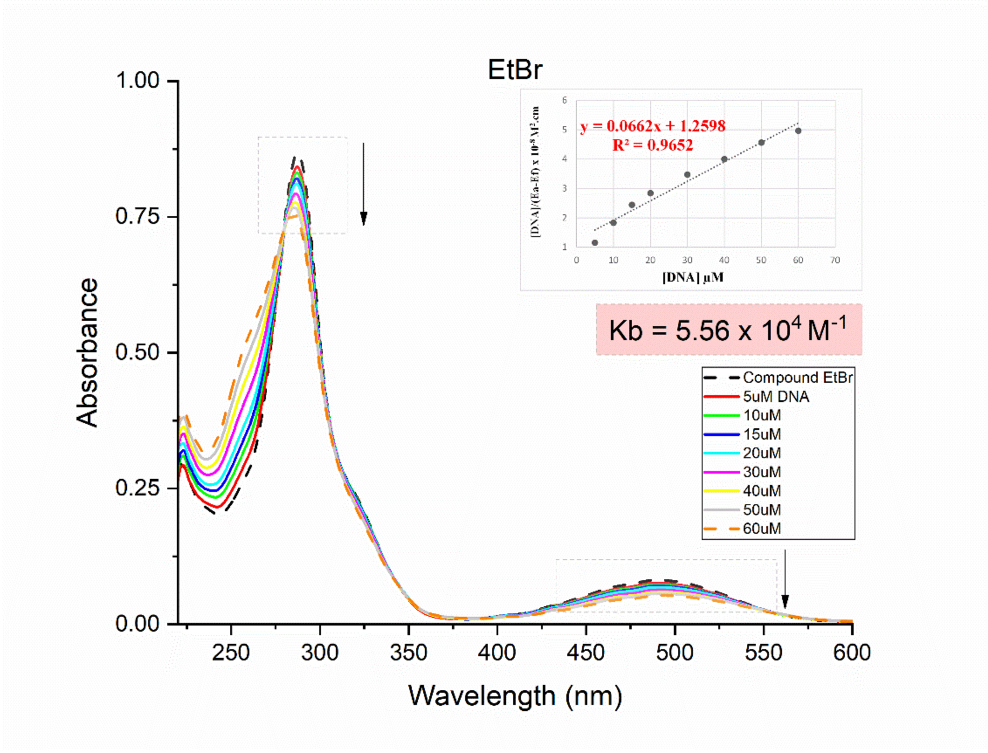

Figure S50. Absorption spectra of **EtBr** at 40 μmol L^{-1} in Tris-HCl buffer (pH 7.4) in the presence of ct-DNA in different concentrations (0, 5, 10, 20, 30, 40, 50 and 60 μmol L^{-1}). λ (Kb) = 489 nm.

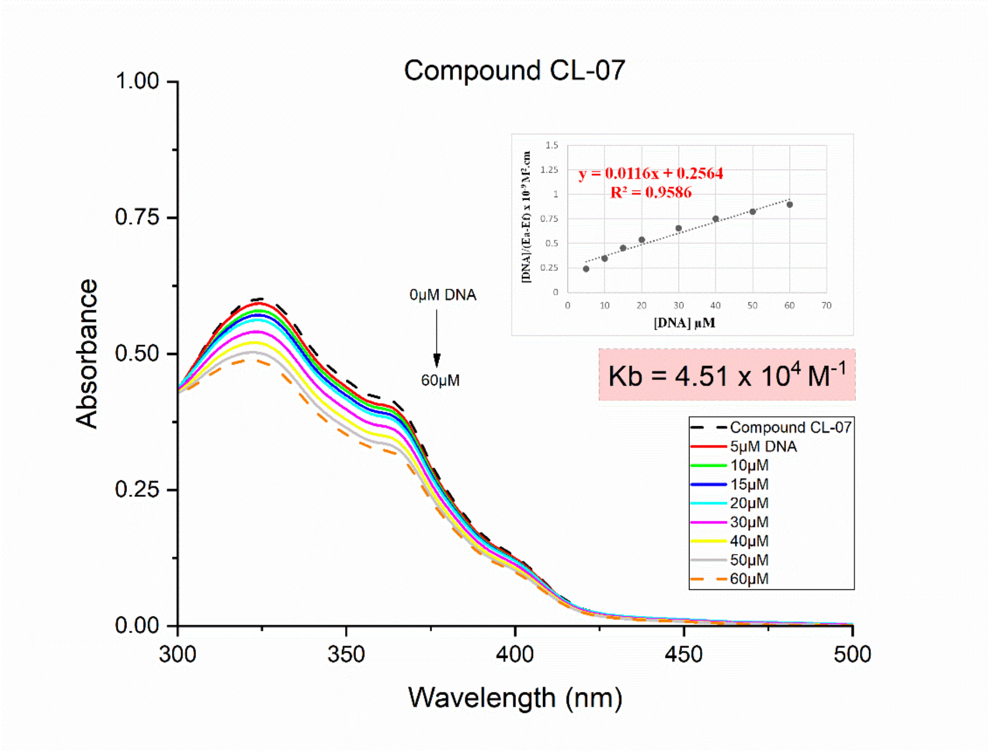

Figure S51. Absorption spectra of **CL-07** at 40 μmol L^{-1} in Tris-HCl buffer (pH 7.4) in the presence of CT DNA in different concentrations (0, 5, 10, 20, 30, 40, 50 and 60 μmol L^{-1}). λ (Kb) = 325 nm.

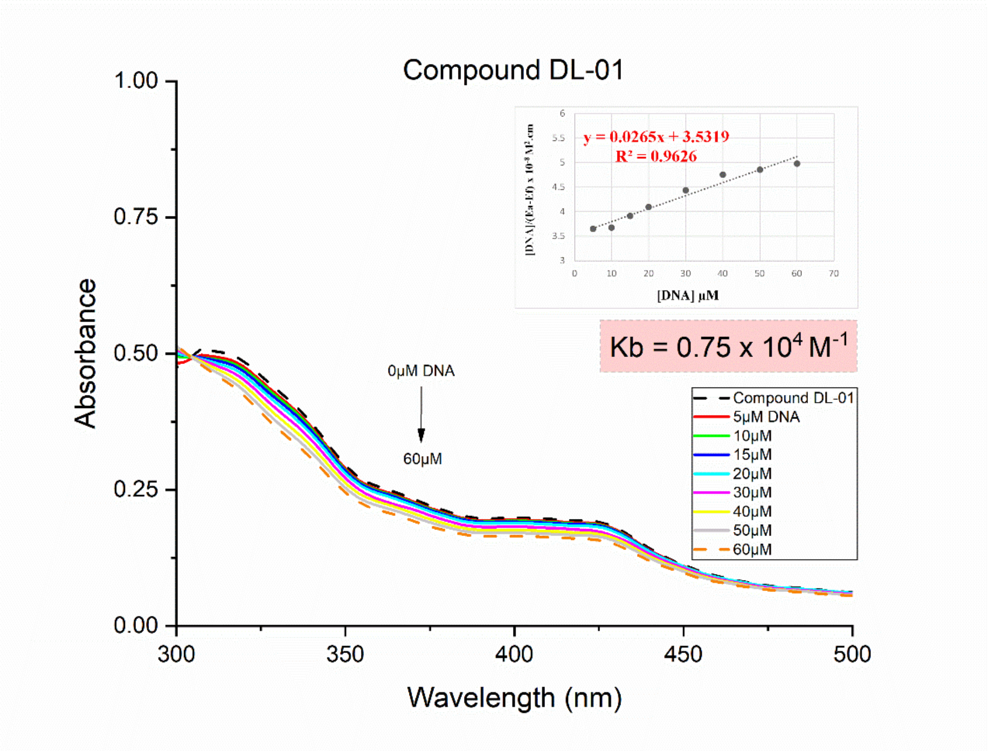

Figure S52. Absorption spectra of **DL-01** at 40 μmol L^{-1} in Tris-HCl buffer (pH 7.4) in the presence of CT DNA in different concentrations (0, 5, 10, 20, 30, 40, 50 and 60 μmol L^{-1}). λ (Kb) = 320 nm.

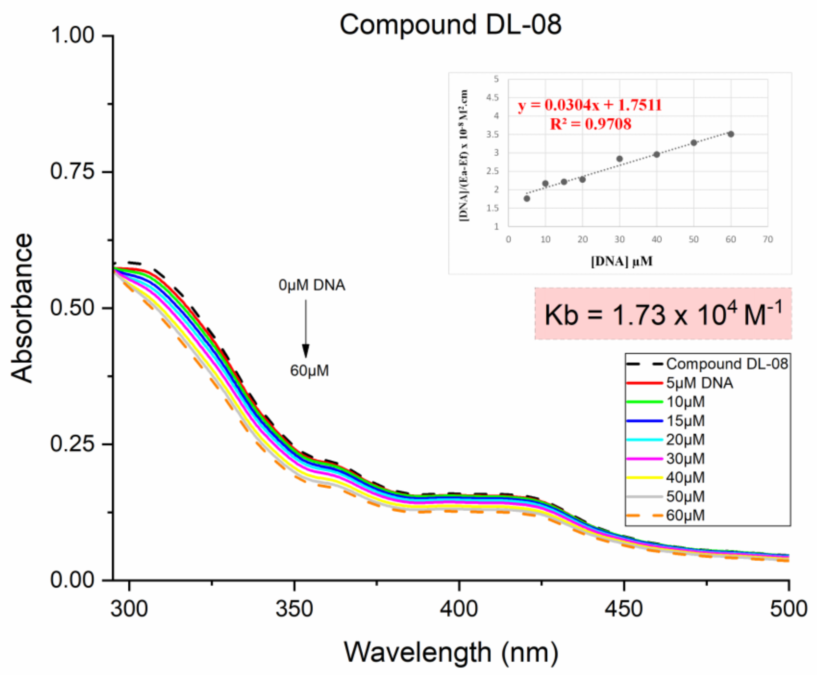

Figure S53. Absorption spectra of **DL-08** at 40 µmol L^{-1} in Tris-HCl buffer (pH 7.4) in the presence of ct-DNA in different concentrations (0, 5, 10, 20, 30, 40, 50 and 60 µmol L^{-1}). λ (Kb) = 305 nm.

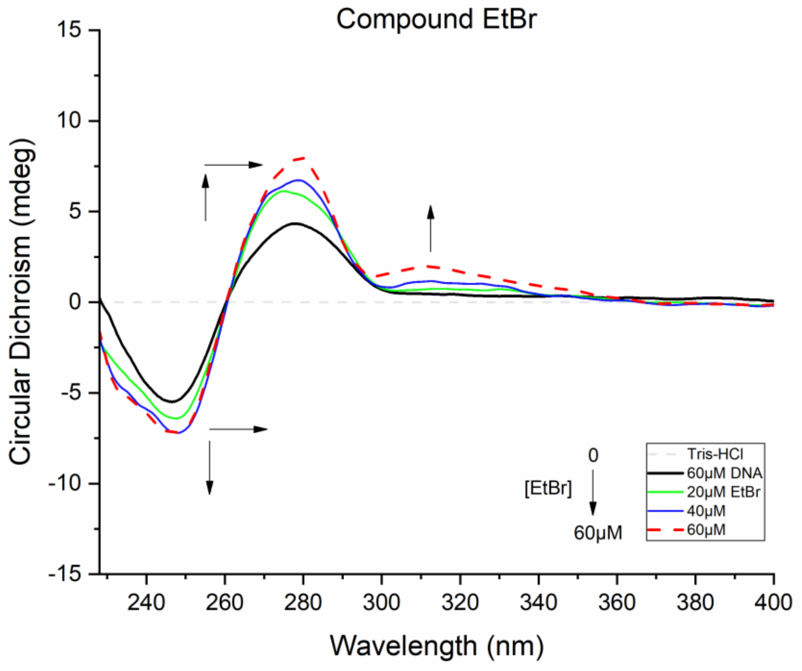

Figure S54. Circular dichroism of DNA at a concentration of 60 µmol L^{-1} in Tris-HCl buffer (pH 7.4), in the presence of increasing concentrations of **EtBr** (20, 40 and 60 µmol L^{-1}).

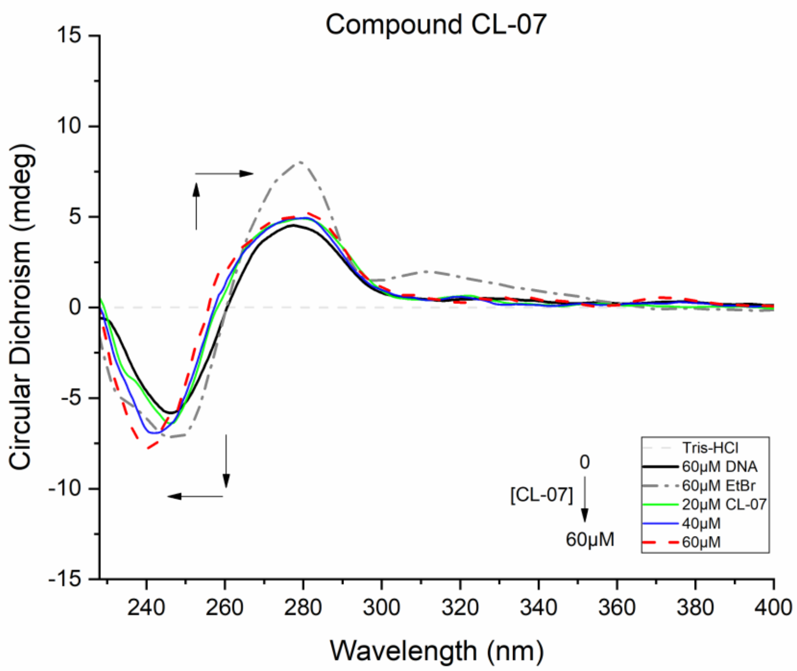

Figure S55. Circular dichroism of DNA at a concentration of 60 μmol L^{-1} in Tris-HCl buffer (pH 7.4), in the presence of increasing concentrations of **CL-07** (20, 40 and 60 μmol L^{-1}).

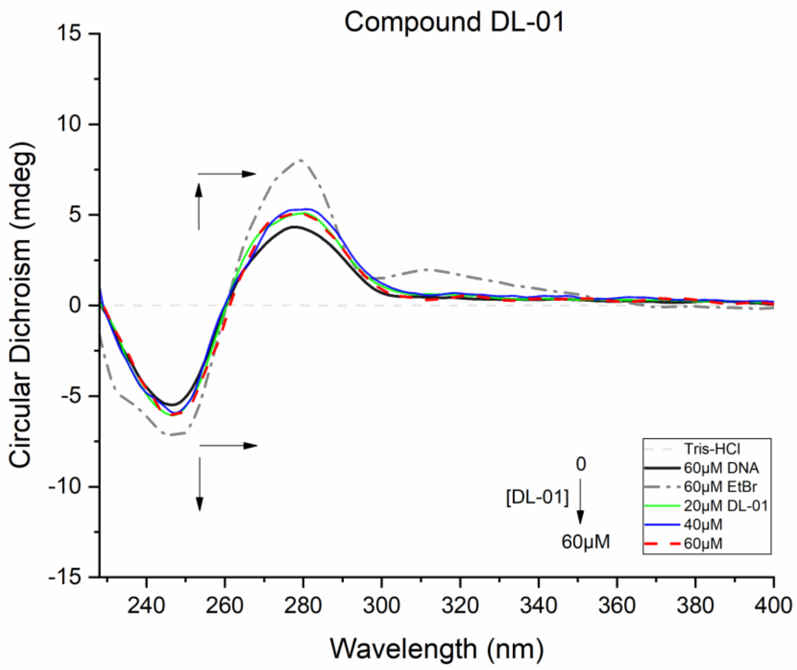

Figure S56. Circular dichroism of DNA at a concentration of 60 μmol L^{-1} in Tris-HCl buffer (pH 7.4), in the presence of increasing concentrations of **DL-01** (20, 40 and 60 μmol L^{-1}).

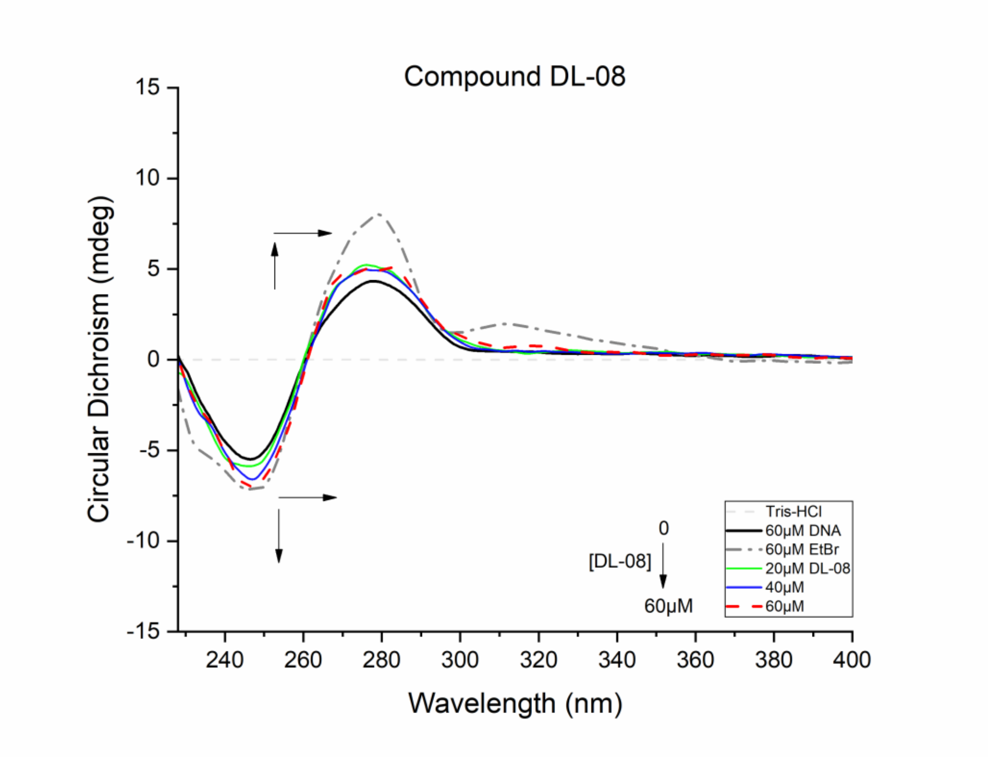

Figure S57. Circular dichroism of DNA at a concentration of 60 µmol L^{-1} in Tris-HCl buffer (pH 7.4), in the presence of increasing concentrations of **DL-08** (20, 40 and 60 µmol L^{-1}).

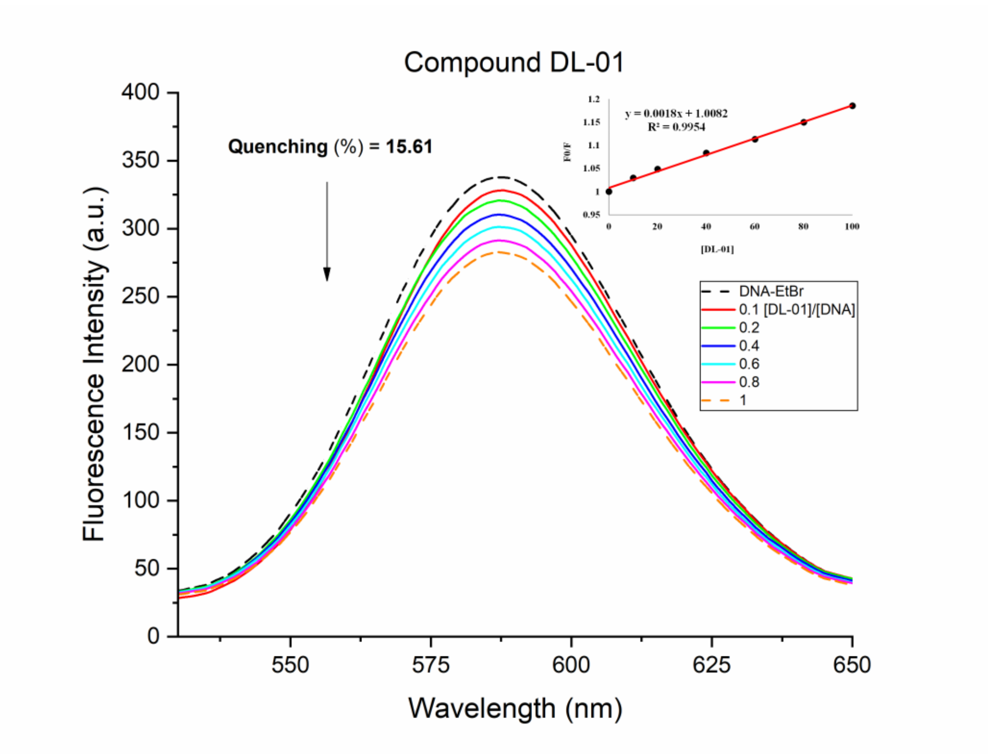

Figure S58. Emission quenching curves of EtBr-DNA by compound **DL-01** ([EtBr] = 20 µmol L^{-1}, [DNA] = 100 µmol L^{-1}, [DL-01] = 0 –100 µmol L^{-1} (λex = 520 nm).

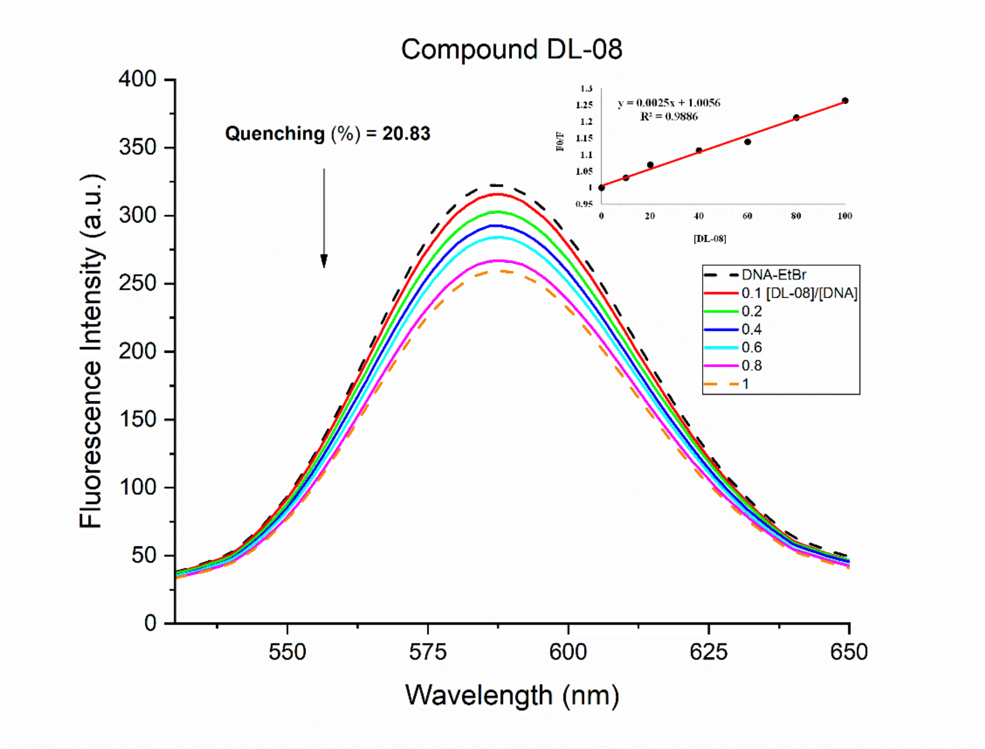

Figure S59. Emission quenching curves of EtBr-DNA by compound **DL-08** ([EtBr] = 20 μmol L^{-1}, [DNA] = 100 μmol L^{-1}, [DL-01] = 0 – 100 μmol L^{-1} (λex = 520 nm).

Table S1. Absolute and relative organ mass from animals treated with acridine derivatives after an acute toxicity study.

Organ mass	Liver (g)	Lungs (g)	Speleen (g)	Heart (g)	Kidneys (g)
**CL-07**	6,135±0,287[a]	0,619±0,089 [b]	0,574±0,061[c]	0,417±0,038[d]	1,308±0,099[e]
**DL-01**	5,257±0,610[a]	0,640±0,057 [b]	0,551±0,037[c]	0,410±0,039[d]	1,338±0,091[e]
**DL -08**	5,619±0,259[a]	0,646±0,032 [b]	0,412±0,033[c]	0,427±0,035[d]	1,355±0,062[e]
**NC**	4,959±0,191[a]	0,628±0,027 [b]	0,408±0,037[c]	0,409±0,040[d]	1,284±0,040[e]

(NC) Control vehicle. In the same category and treatment, the means followed by unequal letters, differ statistically from each other by the Student's T test ($p<0.05$), in relation to the treated and control groups.

Table S2. Feed, water consumption and weight gain in animals treated with compounds during acute toxicity study.

Physiological data	Food (g/day)	Water (mL/day)	Weight gain (g)
**CL-07**	14,57±1,78[a]	23,57±1,34[c]	1,33±0,27 [e]
**DL-01**	12,35±1,39[b]	23,71±1,26[c]	0,69±0,58[f]
**DL -08**	15,21±0,97[a]	25,28±0,46[d]	0,76±0,43[f]
**NC**	18,64±3,52[a]	23,14±1,51[c]	1,00±0[f]

(NC) Control vehicle. In the same category and treatment, the means followed by unequal letters, differ statistically from each other by the Student's T test ($p<0.05$), in relation to the treated and control groups.
